# Specific-cytokine associations with outcomes in knee osteoarthritis subgroups: breaking down disease heterogeneity with phenotyping

**DOI:** 10.1186/s13075-023-03244-y

**Published:** 2024-01-11

**Authors:** Joan Calvet, Antoni Berenguer-Llergo, Cristóbal Orellana, María García-Manrique, Menna Rusiñol, Silvia Garcia-Cirera, Maria Llop, Marta Arévalo, Alba Garcia-Pinilla, Carlos Galisteo, Cristina Aymerich, Rafael Gómez, Alejandra Serrano, Anna Carreras, Jordi Gratacós

**Affiliations:** 1 Department of Rheumatology, Parc Taulí Hospital Universitari, Institut d’Investigació i Innovació Parc Taulí (I3PT-CERCA), Universitat Autònoma de Barcelona, c/Parc Taulí s/n, edifici VII Centenari, 08208 Sabadell, Spain; 2 Department of Medicine, Universitat Autònoma de Barcelona (UAB), Barcelona, Spain

**Keywords:** Knee osteoarthritis, Cytokines, Phenotype, Inflammation, Clinical severity, Radiographic progression, Machine learning

## Abstract

**Background:**

Despite existing extensive literature, a comprehensive and clinically relevant classification system for osteoarthritis (OA) has yet to be established. In this study, we aimed to further characterize four knee OA (KOA) inflammatory phenotypes (KOIP) recently proposed by our group, by identifying the inflammatory factors associated with KOA severity and progression in a phenotype-specific manner.

**Methods:**

We performed an analysis within each of the previously defined four KOIP groups, to assess the association between KOA severity and progression and a panel of 13 cytokines evaluated in the plasma and synovial fluid of our cohort’s patients. The cohort included 168 symptomatic female KOA patients with persistent joint effusion.

**Results:**

Overall, our analyses showed that associations with KOA outcomes were of higher magnitude within the KOIP groups than for the overall patient series (all *p*-values < 1.30e−16) and that several of the cytokines showed a KOIP-specific behaviour regarding their associations with KOA outcomes.

**Conclusion:**

Our study adds further evidence supporting KOA as a multifaceted syndrome composed of multiple phenotypes with differing pathophysiological pathways, providing an explanation for inconsistencies between previous studies focussed on the role of cytokines in OA and the lack of translational results to date. Our findings also highlight the potential clinical benefits of accurately phenotyping KOA patients, including improved patient stratification, tailored therapies, and the discovery of novel treatments.

**Supplementary Information:**

The online version contains supplementary material available at 10.1186/s13075-023-03244-y.

## Background

Osteoarthritis (OA) is a prevalent musculoskeletal disease that affects millions of people worldwide [1], with knee OA (KOA) being the most affected location and the focus of extensive research in recent years [2, 3]. Patients with OA often experience high levels of pain and disability, which result in seeking healthcare assistance [1–3]. The disease is also associated with numerous comorbidities, particularly cardiovascular risk factors, contributing to high healthcare costs [3–5].

The pathophysiology of OA is not fully understood, but it is known that age, obesity, genetics, previous trauma, metabolic factors, some molecular determinants of cartilage degradation, and systemic and local inflammation contribute to its onset and progression [5–11]. Sex-related differences have also been identified in OA patients, including prevalence rates, metabolic conditions, inflammatory factors, and levels of pain and functional disability [12, 13]. OA is currently highly prevalent, and its socioeconomic impact is expected to increase in the coming years due to the ageing of the population and increasing rates of obesity in Western societies [5]. Despite extensive research conducted in the past decades, the development of targeted drugs capable of effectively alleviating pain or halting the structural deterioration in OA remains an unmet need [14].

OA is now understood to be a complex, multi-tissue disease that affects various joint structures including the articular cartilage, bone, subchondral bone, synovial membrane, capsule, ligaments, menisci, and periarticular muscles [1, 5]. Inflammation and metabolic factors are recognized as crucial factors in the development and progression of OA [9–11]. Previous studies have focused on identifying specific inflammatory markers, such as adipocytokines and cytokines found in the blood, synovial fluid, and synovial membrane of OA patients, which have been linked to pain, disability, and radiographic changes [15, 16]. However, much of this research has provided inconclusive or inconsistent results regarding the strength and nature of the association between these cytokines and the severity and progression of OA [11, 17–19].

One possible explanation for these inconsistencies and the lack of major translational research may be the heterogeneity of OA patients regarding clinical presentation, exhibit of risk factors and prognosis. In this regard, it has been suggested that OA may not be a single entity, but rather a complex and heterogeneous condition made up of different subgroups (phenotypes) with specific pathophysiological traits (endotypes) [20, 21]. The identification of these phenotypes could lead to better assessment of severity and prognosis biomarkers, resulting in significant clinical implications for patients’ stratification, therapy tailoring, and exploration of novel treatments [22]. In this regard, several groups have described distinct OA phenotypes characterized by the presentation of diverse features, such as clinical parameters [17, 23], transcriptomics [24, 25], metabolomic data [26, 27], and other biochemical markers [18, 28]. Despite these efforts, a comprehensive and clinically relevant classification system for OA has yet to be established.

In KOA, though, it is generally accepted the existence of an inflammatory clinical phenotype characterized by synovitis, increased levels of pain and disability, and a faster rate of disease progression [29, 30]. In the last years, our group has focussed on the metabolic and inflammatory profiles of KOA patients in this inflammatory phenotype [10, 19, 31–34]. In doing so, our objectives were to better understand the inflammatory mechanisms underlying KOA and to identify specific risk and prognostic factors associated with this inflammatory phenotype. As a result, we have recently identified four knee osteoarthritis inflammatory phenotypes (KOIP) using well-established statistical and machine learning methods applied to a cohort of 168 female patients with primary KOA and joint effusion [35]. The analysis included a comprehensive panel of 45 variables describing the patients’ anthropometric and metabolic status, as well as their inflammatory profile measured by a set of 13 cytokines in plasma and synovial fluid. These phenotypes showed marked differences in their anthropometric, metabolic, and inflammatory profiles and demonstrated significant differences in clinical severity and radiographic progression [35].

In this study, we aimed to further characterize the four KOA inflammatory phenotypes (KOIP) recently proposed by our group, by evaluating inflammatory factors linked with the severity and progression of KOA in a KOIP-specific manner. The identification of such factors is of relevance as they might point to different underlying inflammatory mechanisms for the onset and evolution of the disease across these phenotypes. To do so, we assessed the association between the panel of 13 cytokines available in our KOA cohort and the disease’s severity and radiographic progression within each KOIP separately, both in plasma and in synovial fluid.

## Methods

### Patients’ description

The study was carried out on a prospective cohort of 168 female patients with symptomatic primary knee osteoarthritis (KOA) and persistent joint effusion [35]. Plasma and joint fluid samples were available for all patients. We focussed the analysis on female patients to homogenize the study sample, as numerous sex-related differences have been previously reported in KOA regarding metabolic conditions, inflammatory factors, and levels of pain and function disability [12, 13]. Subjects’ inclusion required the presence of symptomatic primary KOA according to the American College of Rheumatology (ACR) criteria, with a defined diagnosis in the outpatient rheumatology visits, aged between 50 and 85 years old, and with joint effusion observed during the physical examination at the recruitment visit and confirmed by ultrasound (≥ 4 mm on midline suprapatellar line). Symptomatic KOA was defined as the presence of pain greater than or equal to 4 on a 10-cm visual analogue scale, despite the use of prescribed analgesic drugs for at least 3 months. The exclusion criteria comprised secondary osteoarthritis, either due to a history of trauma, menisci injury, or previous inflammatory rheumatism; a history of knee surgery; any disease which, in the investigator’s opinion, could interfere with the assessment of pain such as, but not limited to, fibromyalgia or polyneuropathies; systemic glucocorticoid intake in the last 6 months; and intra-articular glucocorticoid or hyaluronic acid injection in the last 3 or 6 months before recruitment, respectively. The recruitment period was from October 2013 to April 2018.

### Samples

Samples from the plasma and joint fluid were extracted at the patient’s recruitment. Collected samples were appropriately processed and stored at − 80 °C, until their use for quantification of cytokines by enzyme-linked immunosorbent assay (ELISA). ELISA assays were conducted according to the manufacturer’s recommendations. Synovial and plasma samples were evaluated for the following cytokines: C-reactive protein (CRP, mg/L), interleukin 6 (IL-6, pg/mL), interleukin 8 (IL-8, pg/mL), tumour necrosis factor alpha (TNF-alpha, pg/mL), nerve growth factor (NGF, pg/mL), calprotectin (ng/mL), leptin (pg/mL), irisin (ng/mL), visfatin (ng/mL), resistin (pg/mL), osteopontin (ng/mL), adiponectin (ng/mL), and omentin (pg/mL). Due to technical reasons related to the ELISA technology (configuration of plates used), these markers could not be assessed at the same time for all patients. To account for potential effects induced by this technical source, and as described previously [35], we corrected the ELISA values previously to any formal statistical analysis (two-step correction), after applying a transformation using the Tukey ladder of powers to symmetrize their distribution and meet the assumptions of the linear model (Additional file 2: Table S2).

### Data collection

Baseline information regarding demographics and anthropometric and metabolic factors was collected for these patients as described previously [35]. Baseline clinical severity is available for these patients as measured by the Knee injury and Osteoarthritis Outcome Scores (KOOS; pain, functional disability, and symptoms) [36], which were used in reversed order to facilitate the interpretation of the results. Ultrasound measurements were collected regarding joint effusion and synovial tissue thickness (mm). The assessments were performed by a single experienced examiner (JC), using Siemens Acuson Antares with a 5–13-MHz linear array transducer and a standardized protocol based on current guidelines and definitions [37–39]. Radiographic severity was measured by the Kellgren-Lawrence (KL) scale [40] and following the OARSI atlas lecture [41], which includes an assessment of osteophytes and joint space narrowing (JSN). This evaluation involved an anteroposterior knee X-ray conducted with the patient in a standing position, performed within the last 18 months before recruitment. The follow-up radiographic evaluation was blind to the results at baseline. Two different clinicians independently conducted readings for a subset of patients. Concordance between the readers was assessed using unweighted Cohen’s kappa, yielding values of 0.884 for KL (135 patients, 95% confidence interval 0.816 to 0.953), 0.931 for osteophytes evaluation (135 patients, 95% confidence interval 0.885 to 0.977), and 0.782 for JSN (30 patients, 95% confidence interval 0.608 to 0.956). Most of the patients (*n* = 143, 85%) underwent a radiographic evaluation during the follow-up to assess their radiographic progression at 2 years. To assess the radiographic progression at 2 years, the majority of patients (85%) underwent a radiographic evaluation after their initial radiography with a median interval of 26 months (over 18 months for 90% and over 24 months for 69% of the patients in the study). Radiographic progression was defined by comparing the follow-up and baseline radiographs, using each of the three different measures available: an increase in the radiographic Kellgren-Lawrence (KL) stage in the follow-up evaluation (KL progression), the appearance of new osteophytes (osteophyte progression), and a reduction in the space between joint bones (JSN progression). More details about patients, samples, and data collection are available in our previous work [35].

### Statistical analysis

Continuous parameters were described by their medians, median absolute deviations, and ranges, while categorical variables were summarized using absolute frequencies and percentages. Associations with KOIP groups and outcomes were assessed using non-parametric methods, namely the Kruskal-Wallis and Mann-Whitney tests for continuous variables and Fisher’s tests for categorical variables.

Univariate associations between cytokines and KOA outcomes were assessed for the overall series and within each KOIP independently. Given the low sample size available, we deliberately opted for non-parametric methods for their robustness against bias due to the extremely high influential values used. These methods included Spearman correlation (SC) [42] (continuous or ordinal outcomes) and Glass rank biserial correlation (GRBCorr) [43] (binary outcomes). Baseline Kellgren-Lawrence (KL) staging and joint space narrowing were treated as ordinal in these analyses. Asymptotic 95% confidence intervals (CI) were computed for SC, while bootstrap intervals were computed for GRBCorr coefficients (1.000 resamples). For binary outcomes, fold changes (FC) of the median groups were also calculated to quantify the magnitude of the cytokines differences between the patient groups, along with their bootstrap 95% confidence intervals. To aid interpretation, FCs below one were inverted and prefixed with a minus sign, so that a negative FC indicates a higher level of the cytokine in the reference group. In each case, statistical significance was assessed with non-parametric asymptotic methods (SC test for continuous or ordinal outcomes; Mann-Whitney test for binary outcomes). No adjustment by multiple contrasts was performed for these analyses, since they are considered as exploratory when examined individually.

These results were graphically represented in a heatmap, where red colour indicated positive correlation, blue represented negative correlation, and colour intensity expressed more extreme values of the correlation coefficients. Colour intensities were saturated to 0.5 and − 0.5 for positive and negative correlation, respectively. For graphical representation, we also used scatter plots (KOOS scores and ultrasound joint effusion) and boxplots and stripcharts (radiographic progression) where the cytokines were displayed in their transformed scale (Additional file 2: Table S2, see the “Samples” section).

To further examine the associations between cytokines and KOA outcomes, we conducted statistical analyses while controlling for age, disease evolution time, and body mass index (BMI). For continuous KOA outcomes (KOOS-pain, KOOS-functional disability, ultrasound joint effusion, and synovial tissue thickness), we computed adjusted Spearman correlations using probability-scale residuals and cumulative probability models as previously described and implemented [44], along with their corresponding 95% confidence intervals and *p*-values. For binary KOA outcomes (radiographic progression based on KL, osteophytes, and joint space narrowing), each cytokine was individually fitted to a linear model. In these models, patients’ status (progressors or not-progressors) and the confounding variables were included as explanatory factors. To ensure the assumptions of the linear models, we incorporated the cytokines values in their transformed scale (Additional file 2: Table S2) and, when necessary, applied transformations to the confounding variables (Tukey ladder of powers: *g* = 0.25 for disease evolution time; *g* = − 0.75 for BMI; no transformation for age). These methodologies were selected for their robustness to avoid or, at the very least, attenuate biases induced by extreme values. *p*-values were calculated using the Wald test to assess the significance of differences in cytokine levels between the two patient groups. To quantify the association, we extracted the means from the model for each patient group, transformed them back to the original scale of the cytokine, and used these values to estimate a FC between patients with and without radiographic progression. This FC can be interpreted as the ratio of cytokine medians across the patient groups in the original scale of the cytokine [45], assuming that the transformation applied to the response variable allows it to meet the linear model’s assumptions. Confidence intervals for these FCs were computed through simulation from the linear model, following a previously described approach [46].

The magnitudes of the associations observed within each KOIP were compared with those obtained from the whole female patients’ series. To do so, absolute values of the correlation coefficients were computed and compared pair-wise using a Wilcoxon test. The results of these analyses were graphically displayed in a boxplot and a stripchart. The threshold for statistical significance was set at 5%. All analyses were conducted with R [47].

## Results

**Fig. 1:**
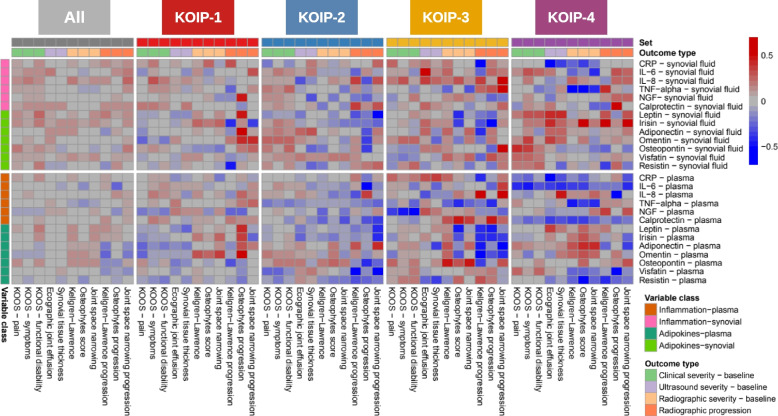
Association of cytokines with knee osteoarthritis (KOA) severity and progression stratified by KOA inflammatory phenotypes (KOIP). The heatmap colours represent non-parametric correlation-like measurements to assess the association of the cytokines evaluated in our study with KOA outcomes, including clinical, radiographic and ultrasound severity at baseline, and radiographic progression at 2 years. Associations were assessed for the overall series and within each KOIP independently using Spearman correlations (continuous or ordinal outcomes) and Glass rank biserial correlations (binary outcomes). Baseline Kellgren-Lawrence staging and joint space narrowing were treated as ordinal in these analyses. Red indicates positive, blue represents negative, and colour intensity expresses more extreme values of the correlation coefficients. Colour intensities were saturated to 0.5 and − 0.5 for positive and negative correlation, respectively. IL-6, interleukin 6; IL-8, interleukin 8; TNF-alpha, tumour necrosis factor alpha; NGF, nerve growth factor; CRP, C-reactive protein; KOOS, Knee injury and Osteoarthritis Outcome Scores (reversed scores); KOA, knee osteoarthritis; KOIP, knee osteoarthritis inflammatory phenotype

Recently, our group identified four distinct inflammatory KOA phenotypes (KOIP) using data from 168 female subjects included in a cohort of primary KOA patients with joint effusion [35]. These phenotypes drastically differed in their anthropometric, metabolic, and inflammatory profiles and exhibited substantial differences in clinical severity and radiographic progression [35]. To gain further insight into these phenotypes and their underlying inflammatory mechanisms, we used the same series (Table 1) to assess, in each of these phenotypes, the association between KOA severity and progression and the panel of 13 cytokines evaluated in the plasma and synovial fluid of our cohort’s patients (Additional file 2: Tables S1 and S2). A global view of these results showed that associations with KOA outcomes were of higher magnitude within the KOIP groups than for the overall patients’ series (all *p*-values < 1.30e−16) and that some of the cytokines showed a KOIP-specific behaviour regarding these associations (Fig. 1, Additional file 1: Fig. S1 and Additional file 2: Tables S3–S7).

**Table 1:** The main baseline patients’ characteristics. Demographic, anthropometric, metabolic, and radiographic factors for all the KOA patients included in the study and stratified by knee osteoarthritis inflammatory phenotypes (KOIP). All subjects are female patients diagnosed with symptomatic primary knee osteoarthritis (KOA) with persistent joint effusion. Continuous parameters are described with their median and ranges (minimum and maximum values), while absolute frequencies and percentages are displayed for categorical variables

		All, 168 (100%)	KOIP-1, 55 (32.7%)	KOIP-2, 51 (30.4%)	KOIP-3, 27 (16.1%)	KOIP-4, 35 (20.8%)	*p*-value
Age at recruitment		69.1 [50.9, 83.0]	70.4 [50.9, 81.4]	68.2 [51.4, 83.0]	66.9 [54.4, 80.8]	70.4 [51.1, 80.5]	0.5195
Kellgren-Lawrence radiographic grade	Grade 1	19 (11.3%)	5 (9.1%)	4 (7.8%)	3 (11.1%)	7 (20.0%)	0.1261
	Grade 2	65 (38.7%)	19 (34.5%)	25 (49.0%)	7 (25.9%)	14 (40.0%)	
	Grade 3	78 (46.4%)	26 (47.3%)	22 (43.1%)	16 (59.3%)	14 (40.0%)	
	Grade 4	6 (3.6%)	5 (9.1%)	0 (0.0%)	1 (3.7%)	0 (0.0%)	
Disease evolution time (months)		48 [4, 200]	45 [4, 150]	48 [4, 200]	36 [6, 125]	60 [6, 172]	0.7291
Obesity		94 (56.0%)	51 (92.7%)	5 (9.8%)	16 (59.3%)	22 (62.9%)	< 0.0001
Physical exercise	None	61 (36.3%)	29 (52.7%)	12 (23.5%)	6 (22.2%)	14 (40.0%)	0.0123
	Sporadic	51 (30.4%)	18 (32.7%)	16 (31.4%)	8 (29.6%)	9 (25.7%)	
	Moderate	46 (27.4%)	6 (10.9%)	18 (35.3%)	11 (40.7%)	11 (31.4%)	
	Vigorous	10 (6.0%)	2 (3.6%)	5 (9.8%)	2 (7.4%)	1 (2.9%)	
Diabetes mellitus		18 (10.7%)	10 (18.2%)	2 (3.9%)	2 (7.4%)	4 (11.4%)	0.1101
Arterial hypertension		92 (54.8%)	37 (67.3%)	18 (35.3%)	16 (59.3%)	21 (60.0%)	0.008
Dyslipidaemia		68 (40.5%)	26 (47.3%)	16 (31.4%)	9 (33.3%)	17 (48.6%)	0.2321
ATP III metabolic syndrome		61 (36.3%)	33 (60.0%)	2 (3.9%)	9 (33.3%)	17 (48.6%)	< 0.0001

*ATP III* Adult Treatment Panel III

**Fig. 2:**
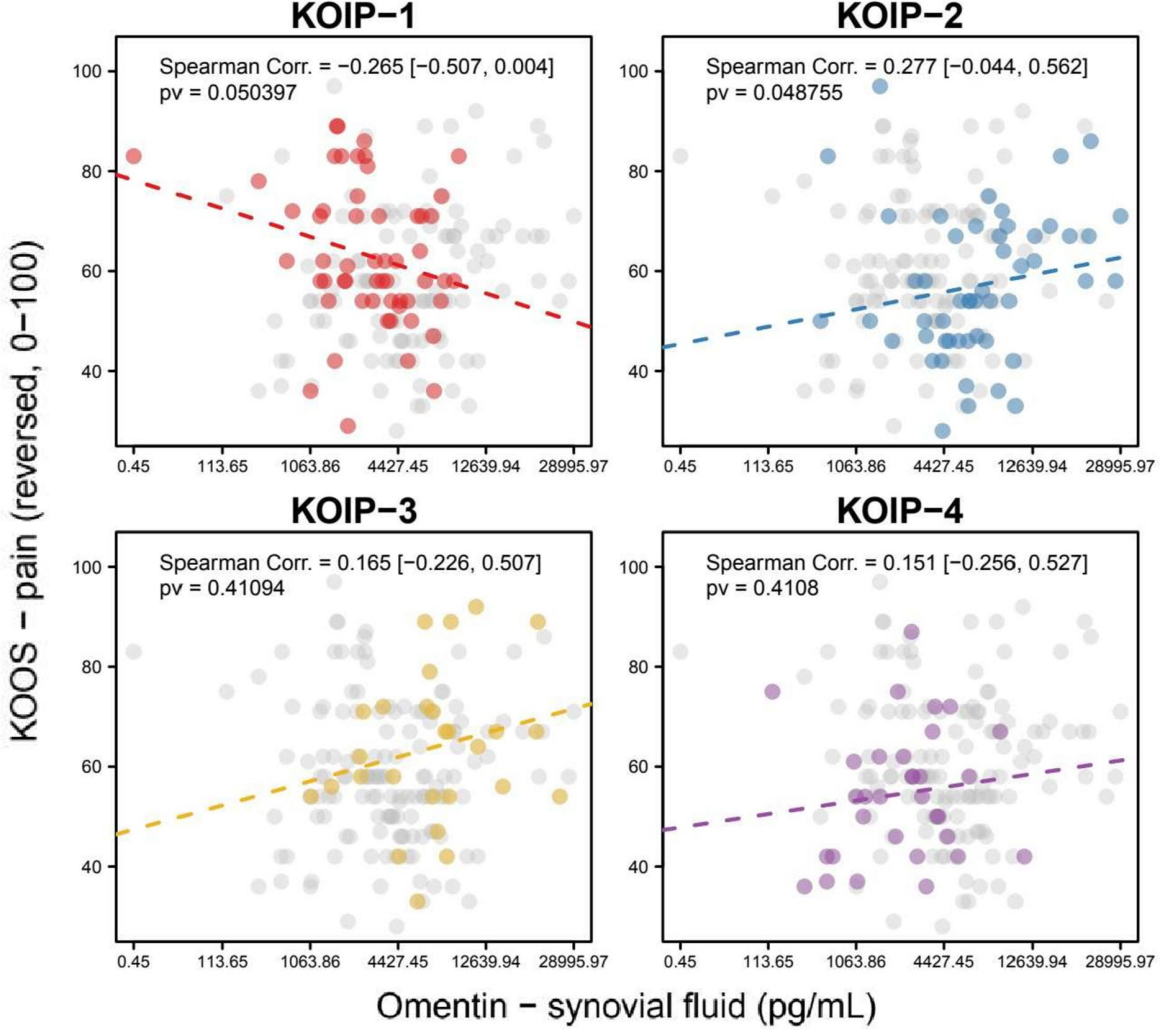
Association of synovial omentin with baseline pain measured by Knee injury and Osteoarthritis Outcome Score (KOOS, reversed score) within each Knee Osteoarthritis Inflammatory Phenotype (KOIP). The panels show the scatter plots for omentin and the KOOS scores in each KOIP group separately, the Spearman correlation coefficient, and its corresponding asymptotic 95% confidence interval (between brackets) and *p*-value. Omentin values are represented in a transformed scale according to Tukey’s ladder of powers, to symmetrize their distribution and make them more suitable for graphical representation (transformation parameter, *g* = 0.25); *x*-axis labels are shown in the original scale. Values from patients not belonging to the indicated KOIP group are represented in grey. Corr., correlation; pv, *p*-value; KOOS, Knee injury and Osteoarthritis Outcome Scores (reversed scores); KOIP, knee osteoarthritis inflammatory phenotype

To illustrate that, we point out some results observed for the clinical severity parameters. In KOIP-1 subjects, a negative correlation was observed for synovial omentin with baseline KOOS pain (Spearman correlation, SC = − 0.265, *p*-value, pv = 0.050) and functional disability (SC = − 0.218; pv = 0.110). In contrast, roughly the same magnitude of positive correlation was found in the KOIP-2 group for both pain (SC = 0.277, pv = 0.049) and functional disability (SC = 0.228, pv = 0.1074) (Fig. 2, Additional file 1: Fig. S2). Other notable findings were the negative association between plasma IL-6 and both pain (SC = − 0.405, pv = 0.016) and function disability (SC = − 0.305, pv = 0.075) in the KOIP-4 group, where synovial osteopontin was also positively correlated with KOOS pain (SC = 0.411, pv = 0.014) (Additional file 1: Figs. S3–S5).

**Fig. 3:**
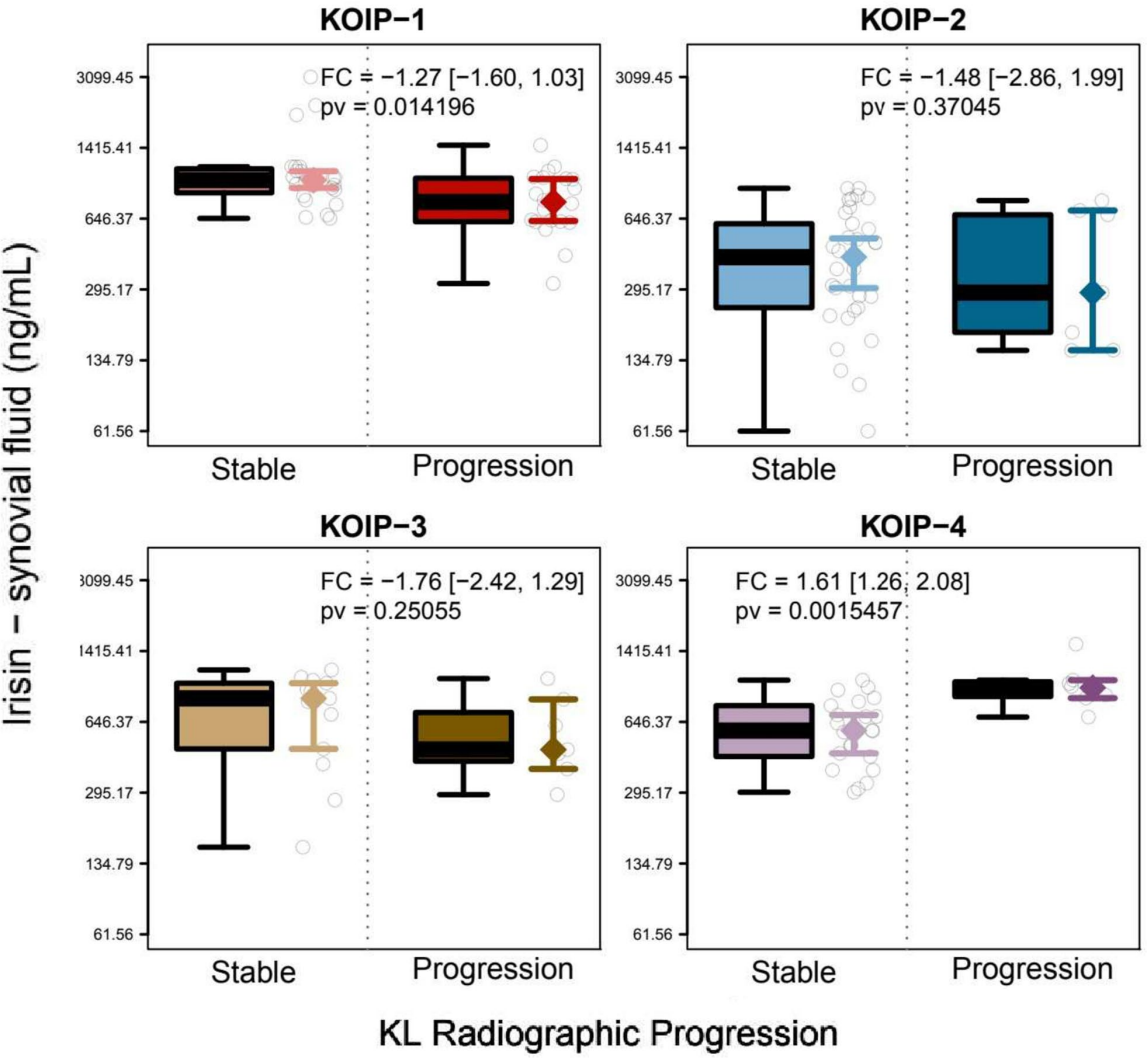
Association of synovial irisin with radiographic progression according to the Kellgren-Lawrence (KL) criteria within each knee osteoarthritis inflammatory phenotype (KOIP). The panels show the boxplots and stripcharts for irisin by patient groups in each KOIP group separately according to the patients’ progression status, as well as the group medians and their corresponding 95% confidence intervals. The legends display the fold changes (FC) between the groups, their 95% confidence intervals (between brackets), and the *p*-value for group comparisons derived from a Mann-Whitney test. A positive FC indicates higher average levels of irisin in progressors while negative FCs represent higher irisin levels in stable patients. Irisin values are represented in a transformed scale according to Tukey’s ladder of powers to symmetrize their distribution and make them more suitable for graphical representation (transformation parameter, *g* = 0); *y*-axis labels are shown in the original scale. FC, fold change; pv, *p*-value; KL, Kellgren-Lawrence; KOIP, knee osteoarthritis inflammatory phenotype

The results stratified by KOIP also showed phenotype-specific associations with radiographic evolution. According to the KL and the JSN criteria, progression was associated with high synovial irisin in KOIP-4 (FC = 1.61, pv = 0.002; FC = 1.53, pv = 0.016, respectively), but also with low levels of this cytokine in KOIP-1 (FC = − 1.27, pv = 0.014; FC = − 1.27, pv = 0.033) (Fig. 3 and Additional file 1: Fig. S20). Based on the KL criteria only, progression was associated with low levels of synovial resistin in KOIP-1 (fold change (FC) = − 1.70, pv = 0.008) and low values of synovial CRP (FC = − 2.32, pv = 0.036) and plasma omentin (FC = − 2.59, pv = 0.029) in KOIP-3 (Additional file 1: Figs. S6–S8).

Synovial omentin and adiponectin showed statistically significant associations with osteophytes progression in KOIP-1 (FC = 1.73, *p*-value = 0.004; FC = 1.83, *p*-value = 0.005, respectively), which were of opposite direction in the KOIP-2 group (FC = − 1.26, *p*-value = 0.059; FC = − 1.27, *p*-value = 0.107, respectively). Osteophyte progression also showed associations of opposite directions for leptin in KOIP-1 (FC = 1.35, pv = 0.025 in the synovial fluid; FC = 1.43, pv = 0.063 in the plasma) and KOIP-3 (FC = − 1.62, pv = 0.360 in the synovial fluid; FC = − 1.67, pv = 0.057 in the plasma), and for plasma IL-8 in KOIP-2 (FC = 1.48, pv = 0.055) and KOIP-4 (FC = − 1.45, pv = 0.022). Other phenotype-specific associations with osteophytes progression were found for osteopontin in KOIP-2 (FC = − 1.22, pv = 0.049 in the synovial fluid; FC = − 1.42, pv = 0.036 in plasma), irisin in KOIP-3 (FC = − 1.95, pv = 0.024 in the synovial fluid; FC = − 1.46, pv = 0.091 in the plasma), and synovial calprotectin in KOIP-4 (FC = 1.80, pv = 0.015) phenotypes (Additional file 1: Figs. S9–S19).

According to the JSN criteria, radiographic progression was also associated with high synovial osteopontin in KOIP-3 (FC = 5.72, pv = 0.009), high synovial omentin in KOIP-1 (FC = 1.81, pv = 0.009), low levels of leptin (FC = − 1.52, pv = 0.007 in synovial fluid; FC = − 1.61, pv = 0.041 in plasma), and low plasma irisin in KOIP-2 patients (FC = − 1.83, pv = 0.038) (Additional file 1: Figs. S20–S25).

**Fig. 4:**
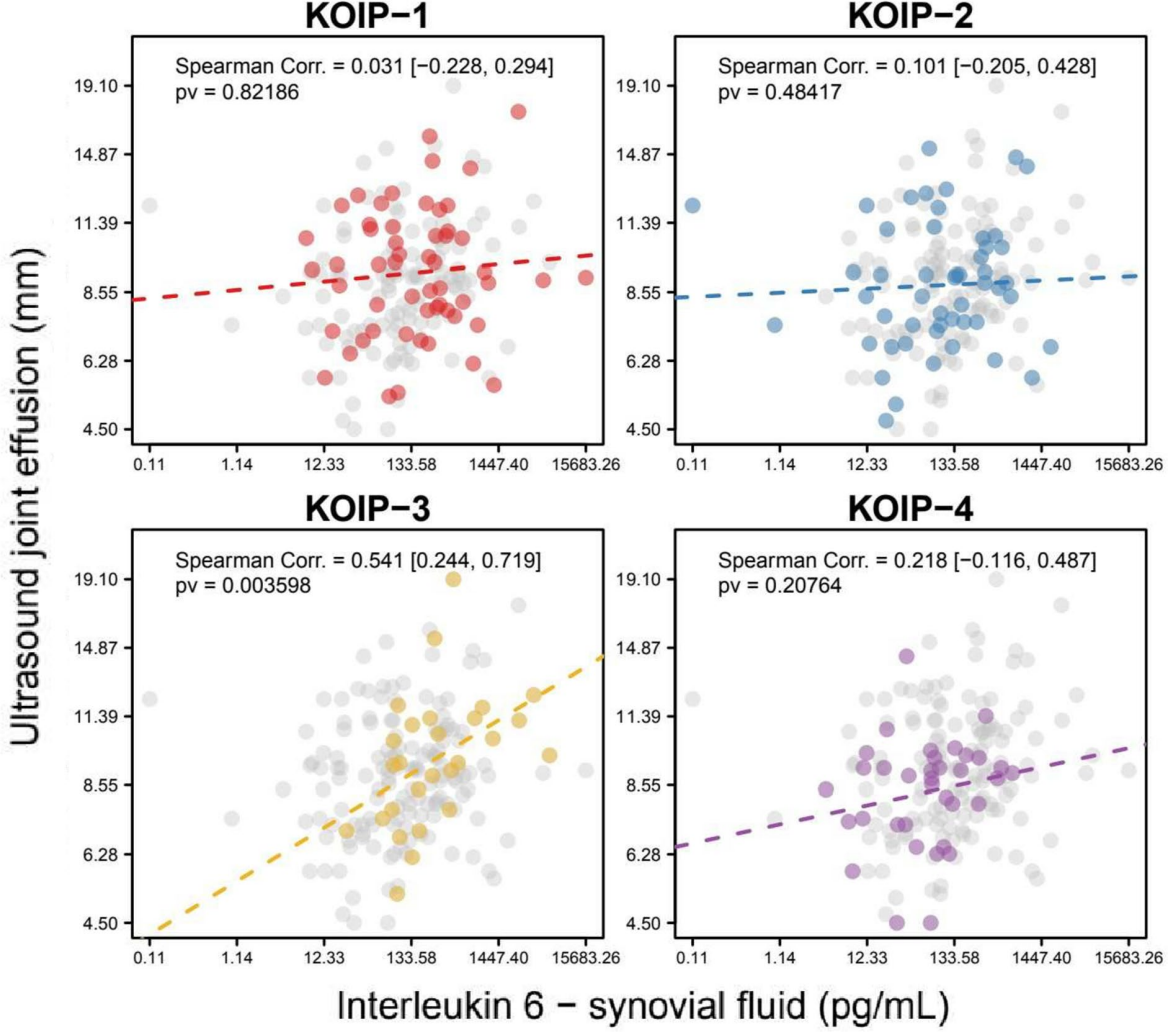
Association of synovial interleukin 6 with joint effusion within each knee osteoarthritis inflammatory phenotype (KOIP). The panels show the scatter plots for interleukin 6 protein and joint effusion (mm) measured by ultrasound in each KOIP group separately, the Spearman correlation coefficient, and its corresponding asymptotic 95% confidence interval (between brackets) and *p*-value. Interleukin 6 protein values are represented in a transformed scale according to Tukey’s ladder of powers, to symmetrize their distribution and make them more suitable for graphical representation (transformation parameter, *g* = 0.25); *x*-axis labels are shown in the original scale. Values from patients not belonging to the indicated KOIP group are represented in grey. Corr., correlation; pv, *p*-value; KOOS, Knee injury and Osteoarthritis Outcome Scores (reversed scores); KOIP, knee osteoarthritis inflammatory phenotype

As other interesting examples, and despite no association was found between the KOIP groups and ultrasound severity in our previous work, some cytokines showed a phenotype-specific correlation with joint effusion, such as synovial IL-6 in KOIP-3 (SC = 0.541, *p*-value = 0.004; Fig. 4). Interestingly, several cytokines were associated with joint effusion in KOIP-4, including positive correlations with leptin (SC = 0.405, pv = 0.016 in the synovial fluid; SC = 0.344, pv = 0.043 in the plasma), synovial irisin (SC = 0.405, pv = 0.016), and omentin (SC = 0.363, pv = 0.041) and negative correlations with CPR (SC = − 0.590, pv = 0.0002 in the synovial fluid: SC = − 0.512, pv = 0.002 in the plasma) and plasma IL-6 (SC = − 0.368, pv = 0.030) and calprotectin (SC = − 0.336, pv = 0.049) (Additional file 1: Figs. S26–S33).

Finally, it is noteworthy that, despite the relatively small sample sizes within each KOIP group (ranging from 27 to 55 patients), the strength and statistical significance of the associations described above (summarized in Additional file 1: Figs. S2–S33) remained largely unchanged in most of the cases after adjusting for age, disease evolution time, and BMI (Additional file 2: Tables S8 and S9).

## Discussion

In this study, we identified a set of cytokines that are differentially associated with severity and radiographic progression across a recently proposed classification of inflammatory phenotypes in KOA (KOIP). In the last years, extensive research has been conducted on the role of several markers on OA severity and progression, including some of the ones evaluated in the present work [10, 11, 15, 17–19, 48]. Although these studies have provided valuable information about the pathophysiology of the disease, none of their results has been transferred to the clinical practice, either to improve the diagnosis or prognosis of their patients or to develop new therapeutic targets with a disease-modifying effect [14]. On the contrary, many of them have provided inconclusive or inconsistent results which, together with the heterogeneity of the disease, has given rise to the hypothesis of the existence of multiple phenotypes in OA [20]. However, despite great efforts have been invested in this line of research, there is still no consensus on a comprehensive classification of OA with clinical relevance.

In our previous work, we identified four different phenotypes of inflammatory KOA that exhibited differential profiles of anthropometric, metabolic, and inflammatory factors and displayed substantial differences in clinical severity and radiographic progression [35]. In the present study, we used this classification as a framework to shed light on the inconsistencies and lack of translational results of previous research. To accomplish this, we assessed the association with severity and progression of a panel of 13 cytokines quantified in the plasma and the synovial fluid of patients with inflammatory KOA, separately for each KOIP group in our cohort. When comparing these results globally, associations with KOA outcomes were significantly of higher magnitude within the KOIP groups than for the overall patients’ series, and a KOIP-specific behaviour was observed for some of the analysed cytokines. In our opinion, the primary significance of these results does not lay in the results of these specific cytokines themselves, whose interpretation is limited by the sample size, but rather in underscoring the crucial role of phenotyping in advancing our comprehension of the disease.

For purely illustrative purposes, we point to the case of omentin, which has been studied by us and others with mixed results [10, 35, 49, 50]. In our previous study, we showed that extreme values of these cytokines contributed to characterize phenotypes in agreement with their metabolic profile (high for KOIP-2 and KOIP-3 and low for KOIP-1 and KOIP-4), but with different levels of clinical severity (more severe in KOIP-1 and KOIP-3 than in KOIP-2 and KOIP-4) [35]. In this work, we also showed that this cytokine displays an association with severity and radiographic progression of opposite sign depending on the KOIP considered (positive in KOIP-2 but negative in KOIP-1). These stratified analyses revealed several other examples of such KOIP-specific associations with KOA outcomes for the evaluated cytokines, both in the plasma and the synovial fluid, which are provided in the “Results” section and the supplementary material of this work. Importantly, most of these associations retained their magnitude and statistical significance after adjustment by age, disease evolution time, and BMI. While the analyses in this study were based on a small number of patients (ranging from 27 to 55) and should be interpreted with caution, their results provide further evidence of specific risk and prognosis factors across these KOIP phenotypes and divergent pathophysiological pathways and disease evolution (endotypes). These results also suggest that differential inflammatory mechanisms may be responsible for the inconsistencies observed in previous research on OA biomarkers and, therefore, the current lack of translational results, likely due to variations in the distribution of KOA phenotypes among the subjects selected for these studies [21, 51].

Importantly, OA pathophysiology is probably too complex to be attributed to a few numbers of cytokines [20, 52–54]. Hence, the purpose of our study was not to point the relevance of a specific set of cytokines or inflammatory factors regarding the clinical or radiographic severity in KOA, an objective for which a larger sample size would be required. Rather, we aimed to illustrate the potential of a precise phenotyping in identifying the inflammatory and metabolic pathways of the disease (endotypes). In contrast to other rheumatic conditions, the inflammatory profile of OA patients is characterized by a relatively lower number of markers that are highly altered in its clinical presentation, and the interaction and modulatory effects may play a significant role in this context of a low-grade, persistent inflammation state [16, 55, 56]. It is noteworthy that correlations exceeding 0.25 (in absolute value) between cytokines and outcomes were observed in varying proportions among KOIP groups, ranging from 12% (KOIP-1) to 24% (KOIP-4). When we raised this threshold to 0.40, the percentages ranged from 3% (KOIP-1 and KOIP-2) to 9% (KOIP-3). While these proportions are considerably higher compared to those observed in the entire patient series (1% for > 0.25; 0% for > 0.40), we acknowledge that the effect sizes are not exceptionally large, even within the identified phenotypes. For this reason, the identification of specific biomarkers remains a critical challenge for improving patient classification and elucidating the molecular mechanisms underlying each phenotype. The identification of such biomarkers would have significant implications for research and clinical practice, as they may facilitate tailored treatments and the discovery of new therapeutic targets [22]. In this regard, the use of Omics technologies holds great potential for making advances in this objective, and our group is currently pursuing this line of research.

Our study was conducted on a prospective cohort of female KOA patients with joint effusion, which constitutes a highly homogeneous group of subjects. Our study was focused on female patients, as they were the majority in our cohort (84%), and several sex-specific differences exist in terms of prevalence, metabolic and inflammatory conditions, and pain and disability levels [12, 13]. Although this homogeneity can provide an advantage for identifying disease biomarkers, we acknowledge that it might also limit the generalization of our results. Hence, further studies are needed in independent series of patients from other centres, with a sufficient sample size and different characteristics and clinical presentations, including males and non-inflammatory presentations, in order to evaluate the generalization of these findings. In addition, and as highlighted earlier in this section, our study’s primary objective was not to emphasize the relevance of specific cytokines in relation to clinical or radiographic severity in KOA, an objective that would require a larger sample size. Beyond the general picture represented in Fig. 1 and Additional file 1: Fig. S1, the results for each cytokine are considered exploratory when considered individually and that was the reason for not adjusting them for the large number of comparisons performed. Hence, the interpretation of these results at the cytokine level should be approached with caution, as they also require further validation in future studies specifically designed for this purpose. Together with the moderate effect sizes found in these analyses and the scarce knowledge in the literature on KOA phenotypes and their specific pathophysiology, our study does not allow for strong interpretations at this level, as they would be too speculative. Another limitation in our study is the absence of pure quantitative measurements for radiographic severity, such as the minimum or fixed joint-space width measurements in millimetres over time. These measurements could have provided better resolution, enhance statistical power, and, possibly, reveal additional associations not identified in our current analyses. Unfortunately, this kind of quantification is not currently available in our patients’ series and constitutes a relevant area of research for future studies. On the other hand, our study is distinguished from previously published works by its exhaustive availability of data, including a panel of 13 cytokines quantified in the plasma and synovial fluid of 168 patients. These samples and data were systematically collected within the protocols of a prospective cohort specifically designed to study the factors associated with KOA severity and progression, which is a remarkable strength of the study.

## Conclusion

Overall, our study provides further evidence to support the notion that KOA is a multifaceted syndrome composed of multiple phenotypes with differing pathophysiological pathways, which provide a possible explanation for the inconsistencies observed across previous studies on the role of cytokines in OA and the lack of translational results to the date. Our findings also highlight the potential benefits of accurate phenotyping of KOA patients for both research and clinical practice, including patient stratification, personalized therapy design, patient selection for clinical trials, and the discovery of novel treatments. Moving forward, large-scale studies using Omics-based biomarker technologies are needed to confirm and allow the reproducibility of the KOIP classification, evaluate its generalizability to other patient populations, and precisely determine its clinical relevance.

### Supplementary Information

**Additional file 1.** 

** Additional file 2.**

## Data Availability

All data and code used in this study are available upon reasonable request.
