# Supplementary material for: Specific-cytokine associations with outcomes in knee osteoarthritis subgroups: breaking down disease heterogeneity with phenotyping
Source: Arthritis Res Ther. 2024 Jan 11;26:19. doi: 10.1186/s13075-023-03244-y (PMC10782658; doi:10.1186/s13075-023-03244-y)
Supplement: Supplementary file 1 — Additional file 1. [file 13075_2023_3244_MOESM1_ESM.pdf]

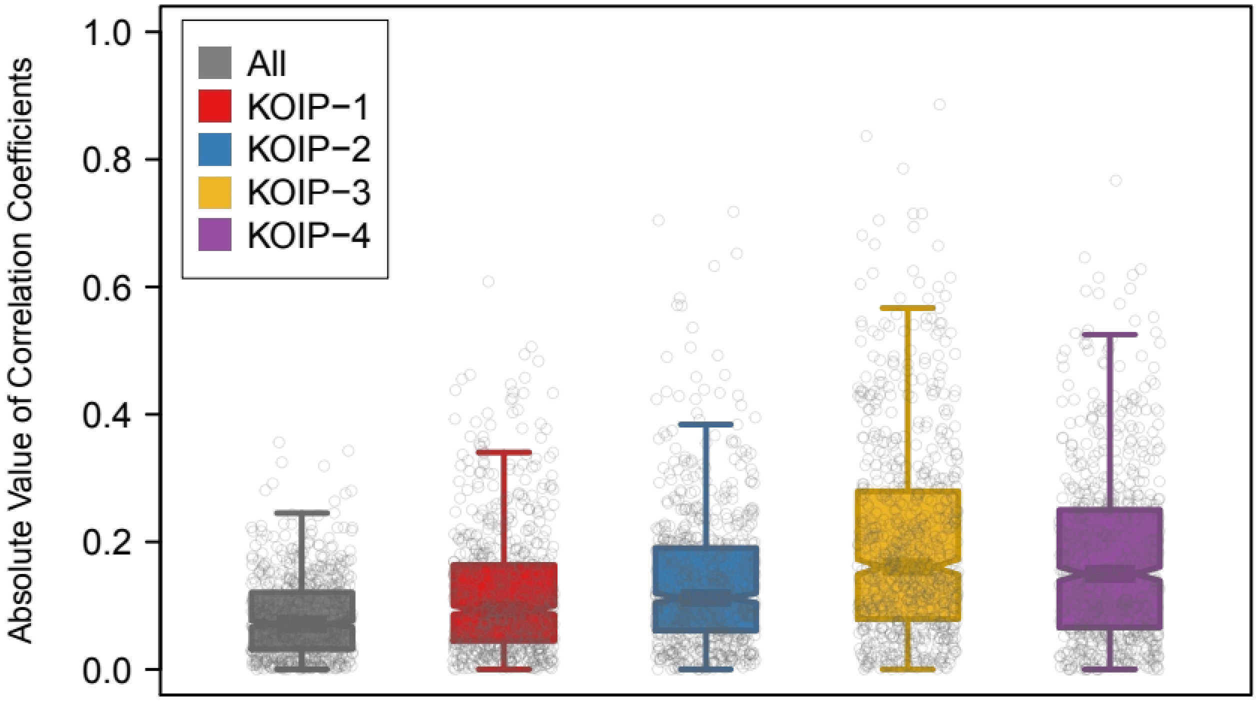

Suplementary Figure S1. Magnitudes of the associations between cytokines and KOA outcomes stratified by Knee Osteoarthritis Inflammatory Phenotypes (**KOIP**). The boxplot shows the absolute value of the correlation-like measurements used for assess the associations between the cytokines evaluated in the study and baseline clinical (KOOS pain, functional disability and symptoms), ultrasound (effusion and synovial tissue thickness), radiographic severity (Kellgren-Lawrence, osteophytes and joint space narrowing), and progression at two years of follow-up according to the same radiographic criteria. Correlation-like measurements were: Spearman correlations (continuous or ordinal outcomes) and Glass rank biserial correlations (binary outcomes). Kellgren-Lawrence staging and joint space marrowing at baseline were treated as ordinal in these analyses. Differences between each KOIP and the whole dataset (*All*) were statistically significant according to a Wilcoxon test (all p-values < 1.30e-16). **KOIP:** Knee Osteoarthritis Inflammatory Phenotype.

Suplementary Figures S2-S33. Association of synovial and plasma **cytokines** with **clinical severity and radiographic progression** within each Knee Osteoarthritis Inflammatory Phenotype (**KOIP**). En each graphic, the panels show scatter plots, the Spearman correlation coefficient and its corresponding asymptotic 95% confidence interval (between brackets) and p-value (baseline pain, functional disability and joint effusion); or boxplots, stripcharts, group medians, Fold-Change (**FC**) between groups, their corresponding 95% confidence interval and the p-value derived from a Mann-Whitney test (radiographic progression). A positive FC indicates higher average levels of Irisin in progressors while negative FCs represents higher Irisin levels in stable patients. Cytokine's values are represented in a transformed scale according to a Tukey's ladder of powers, to symmetrize their distribution and make them more suitable for graphical representation (see Supplementary Table S2); axis value labels are showed in the original scale. **Corr.:** Correlation; **FC:** Fold-Change; **pv:** p-value; **KOOS:** Knee injury and Osteoarthritis Outcome Scores (reversed scores); **KL:** Kellgren-Lawrence; **JSN:** Joint Space Narrowing; **KOIP:** Knee Osteoarthritis Inflammatory Phenotype.

KOOS functional disability
- S2: synovial Omentin
- S3: plasma Interleukin-6

KOOS pain
- S4: synovial Osteopontin
- S5: plasma Interleukin-6

KL radiographic progression
- S6: synovial Resistin
- S7: synovial C-reactive protein
- S8: plasma Omentin

Osteophytes radiographic progression
- S9: synovial Irisin
- S10: synovial Leptin
- S11: synovial Adiponectin
- S12: synovial Omentin
- S13: synovial Osteopontin
- S14: synovial Calprotectin
- S15: plasma Irisin
- S16: plasma Leptin
- S17: plasma Omentin
- S18: plasma Osteopontin
- S19: plasma Interleukin-8

JSN radiographic progression
- S20: synovial Irisin
- S21: synovial Leptin
- S22: synovial Omentin
- S23: synovial Osteopontin
- S24: plasma Irisin
- S25: plasma Leptin

Ultrasound Joint Effusion
- S26: synovial Irisin
- S27: synovial Leptin
- S28: synovial Omentin
- S29: synovial C-readtive protein
- S30: plasma Leptin
- S31: plasma Calprotectin
- S32: plasma Interleukin-6
- S33: plasma C-reactive protein

S2: KOOS functional disability - synovial Omentin

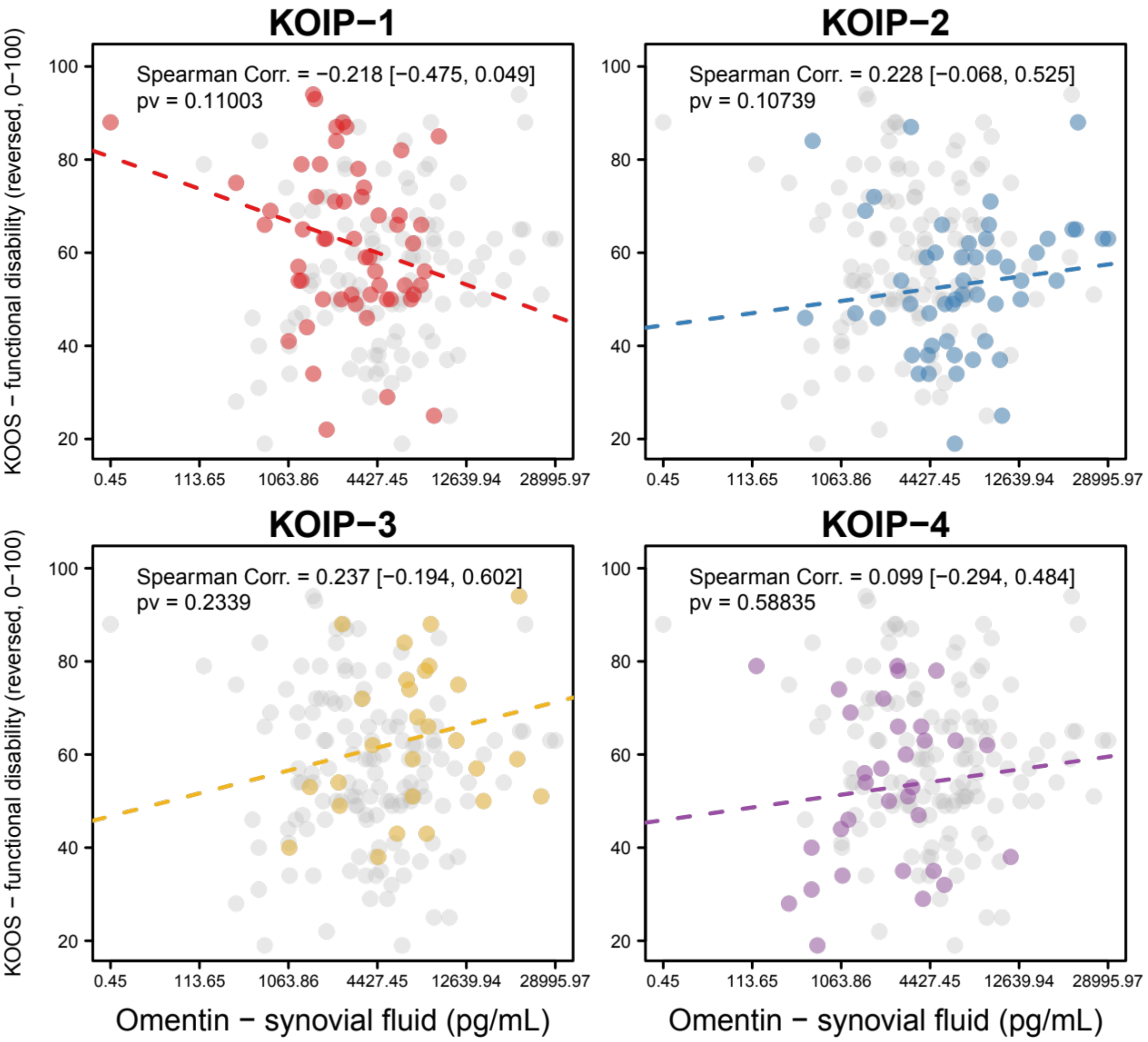

[Back to top](#)

S3: KOOS functional disability - plasma Interleukin-6

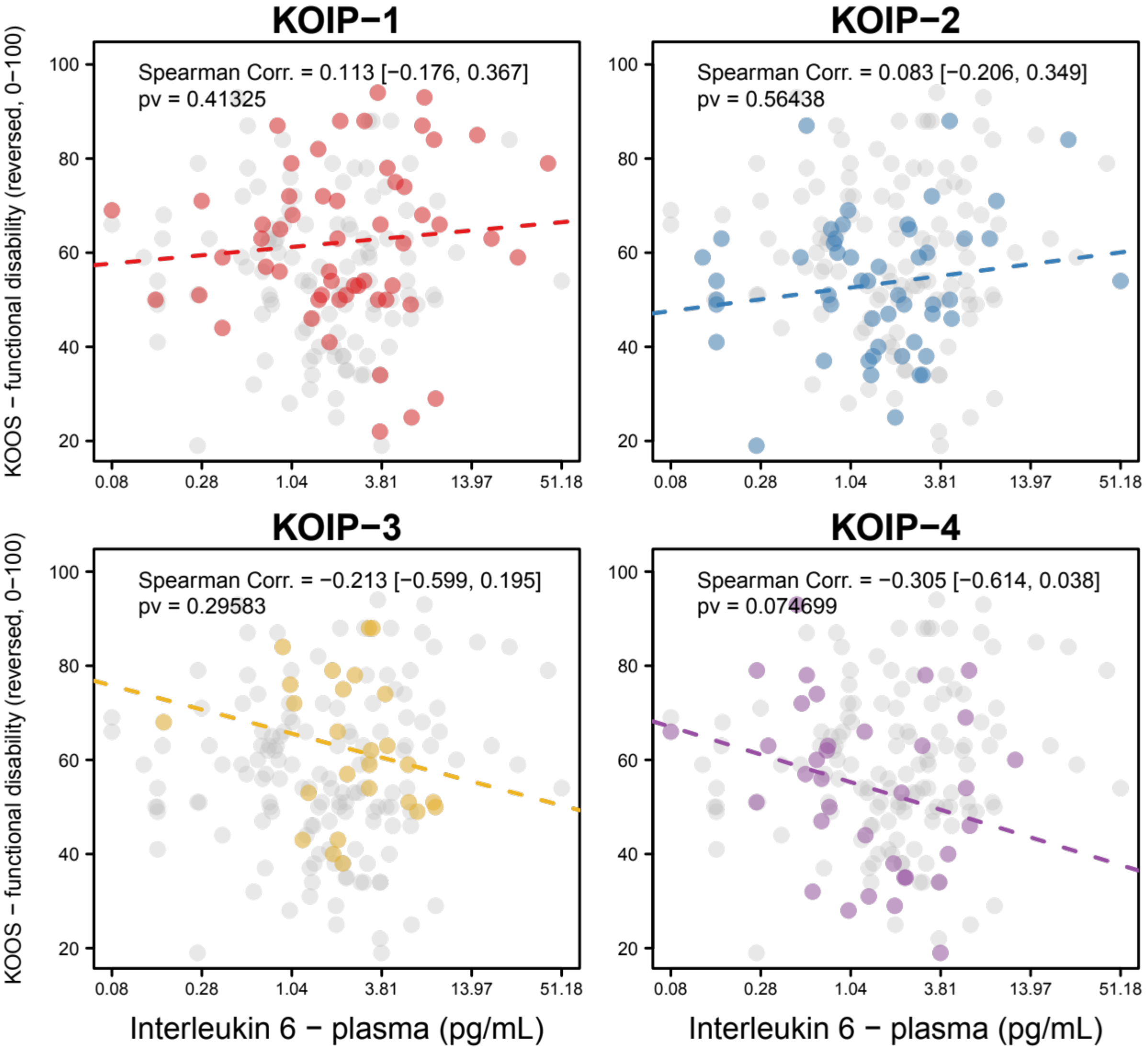

Back to top

S4: KOOS pain - synovial Osteopontin

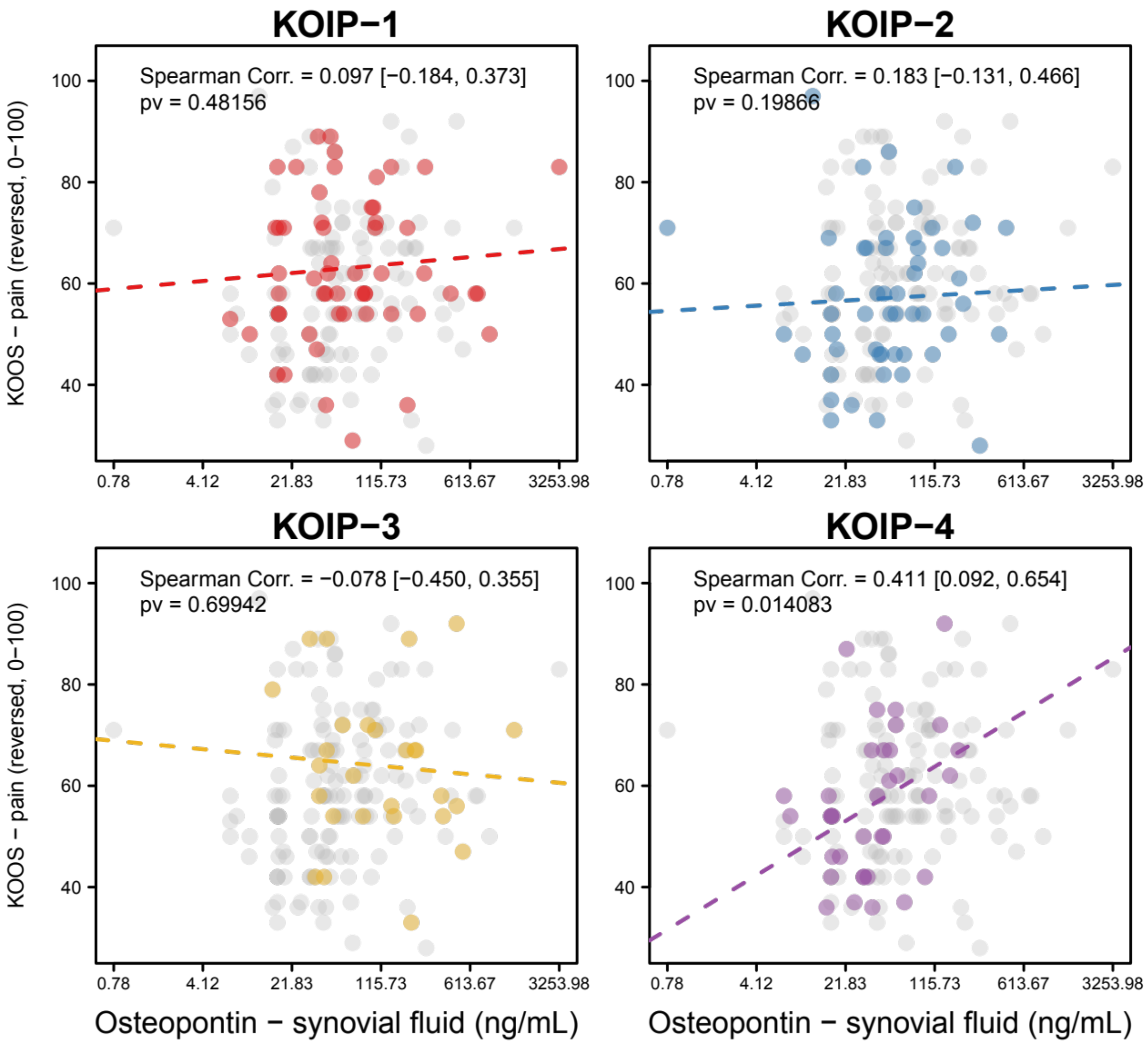

[Back to top](#)

S5: KOOS pain - plasma Interleukin-6

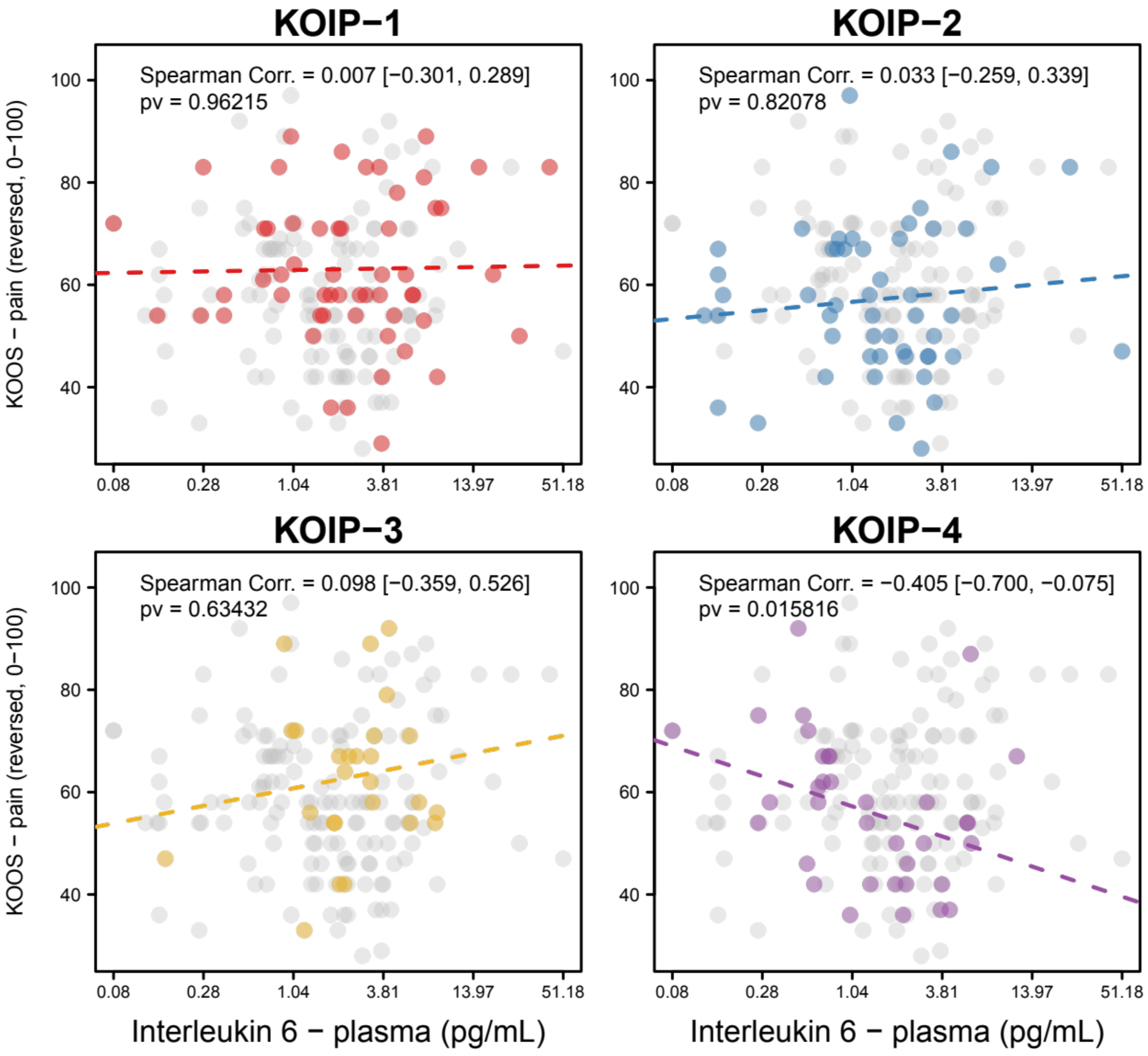

[Back to top](#)

S6: KL radiographic progression - synovial Resistin

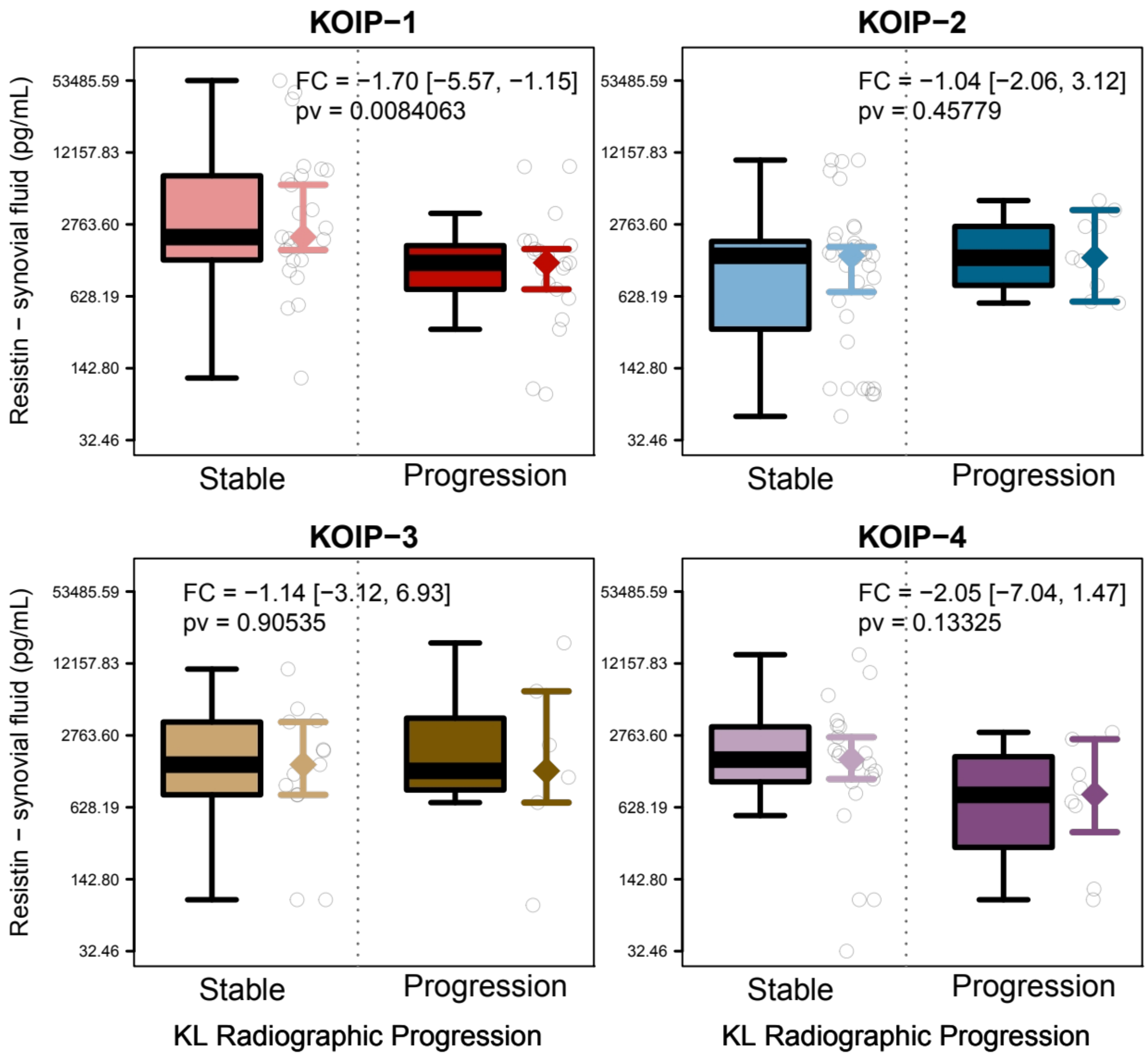

[Back to top](#)

S7: KL radiographic progression - synovial C-reactive protein

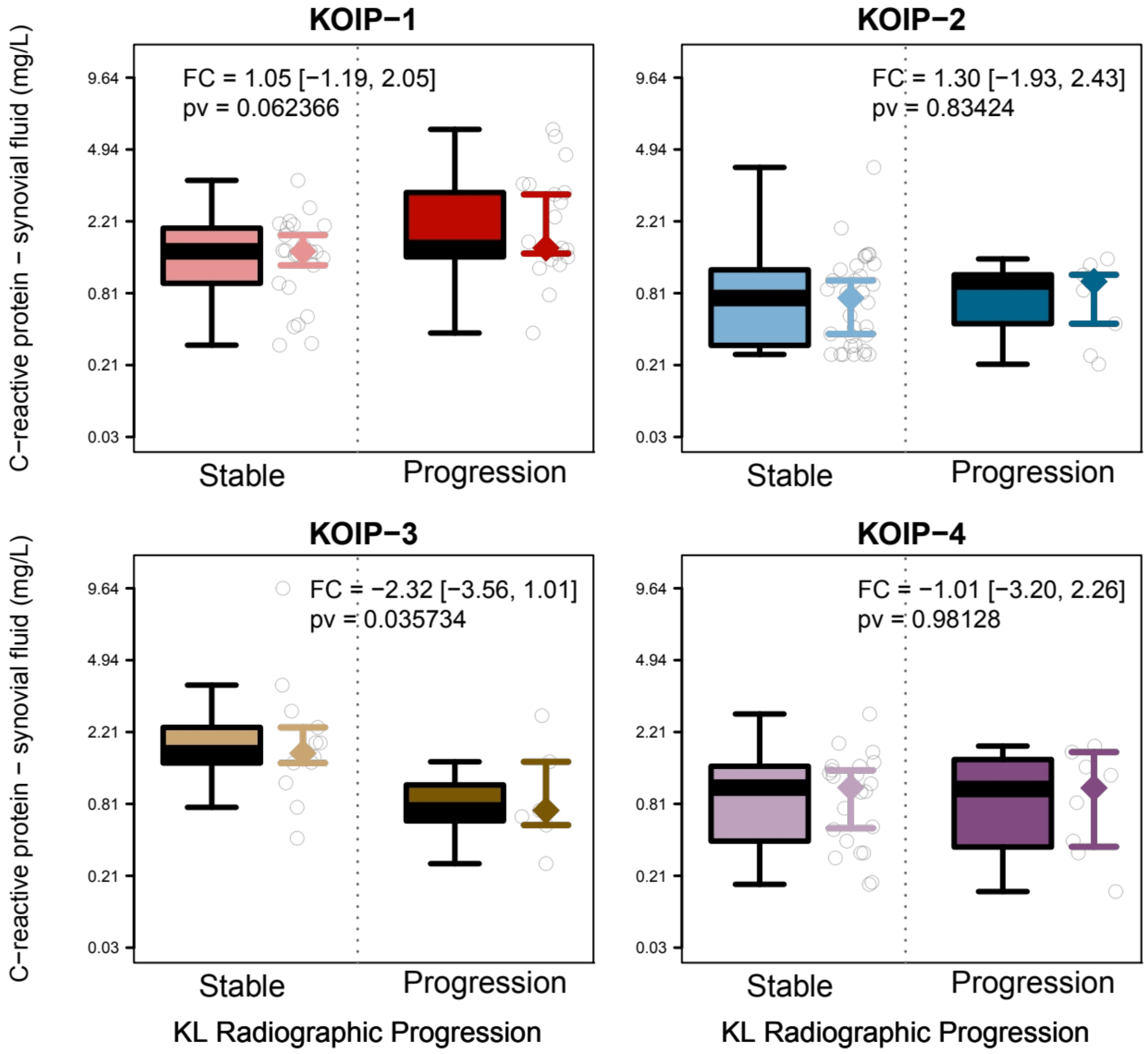

[Back to top](#)

S8: KL radiographic progression - plasma Omentin

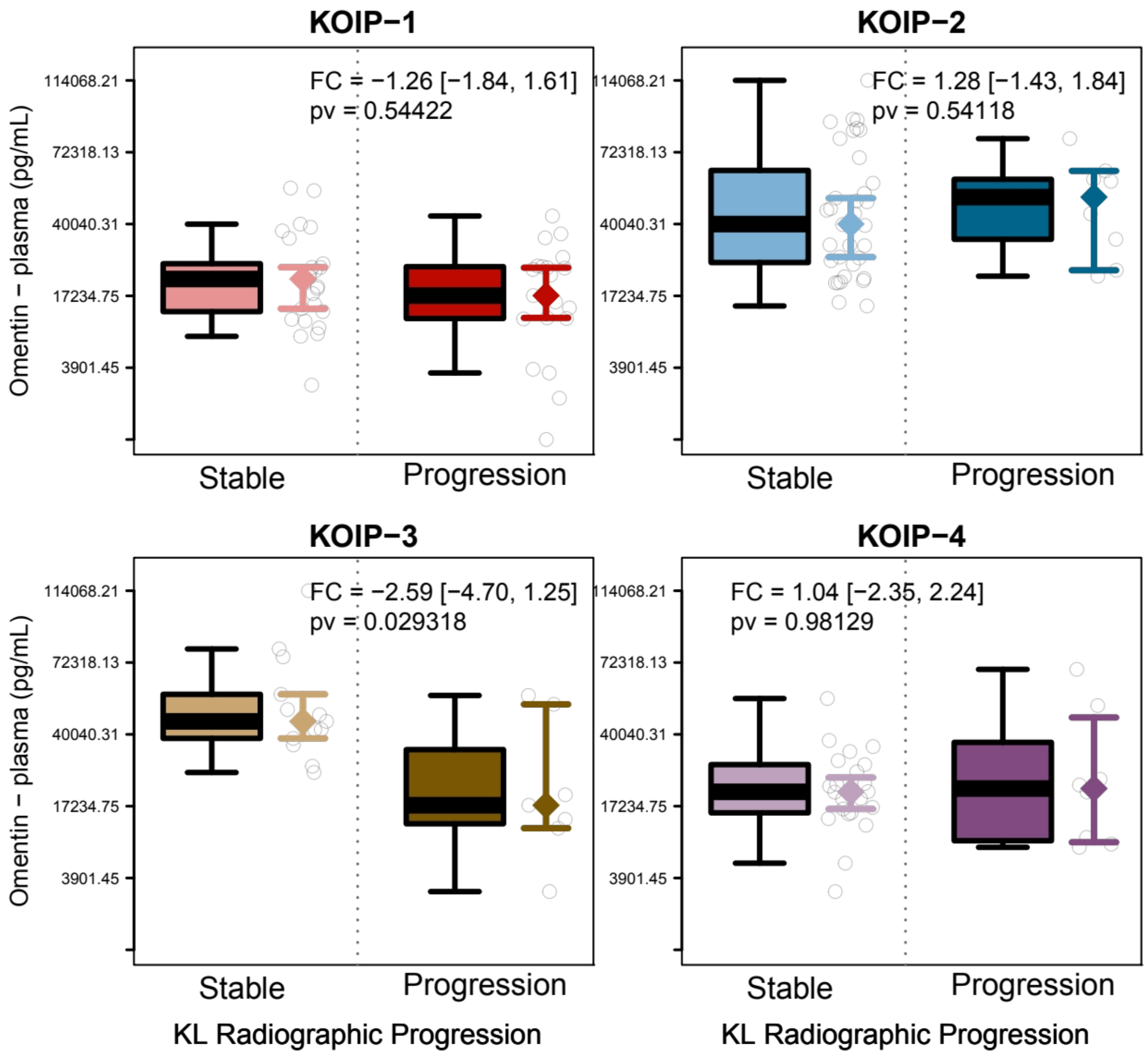

[Back to top]

S9: Osteophytes radiographic progression - synovial Irisin

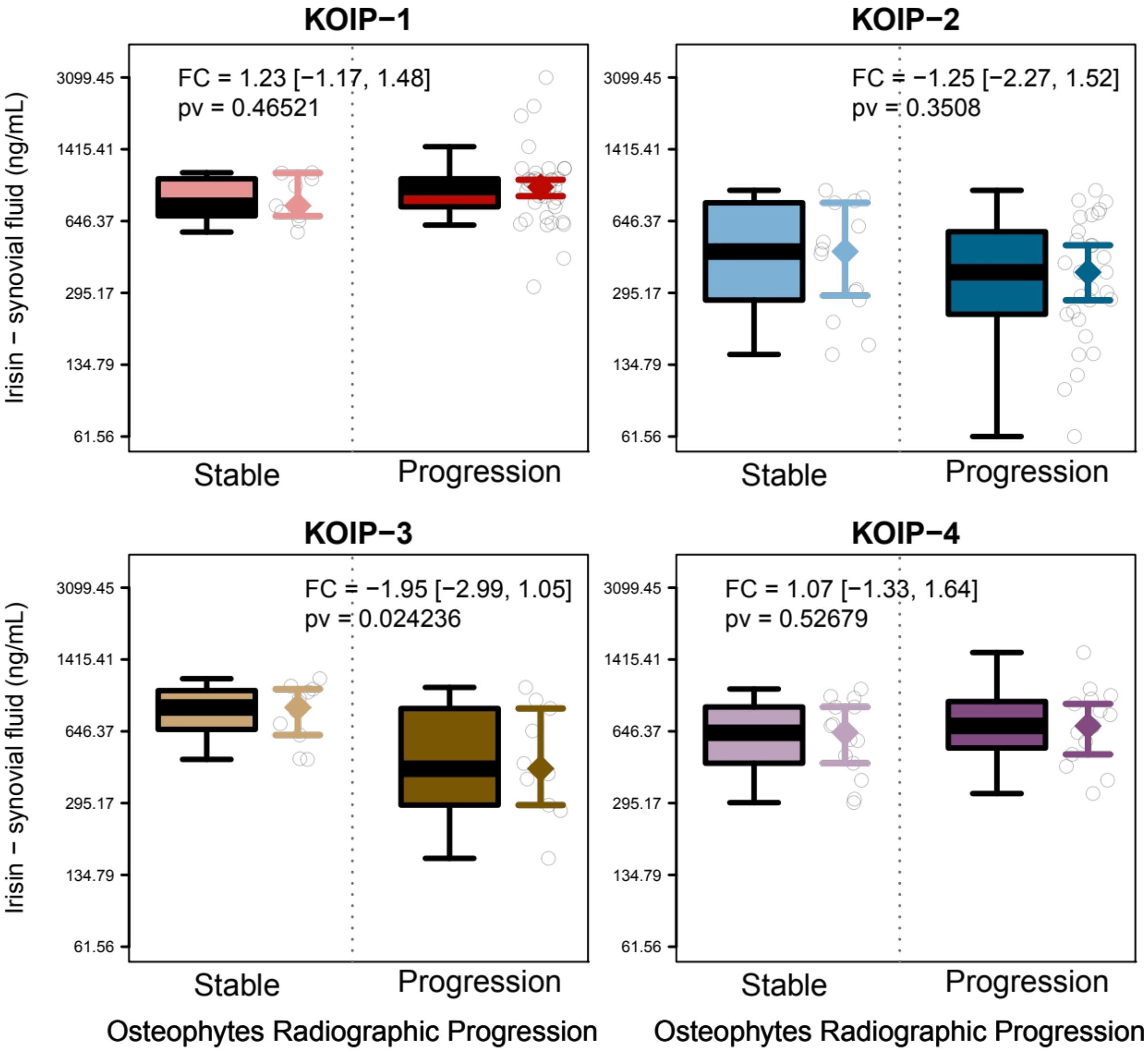

Back to top

S10: Osteophytes radiographic progression - synovial Leptin

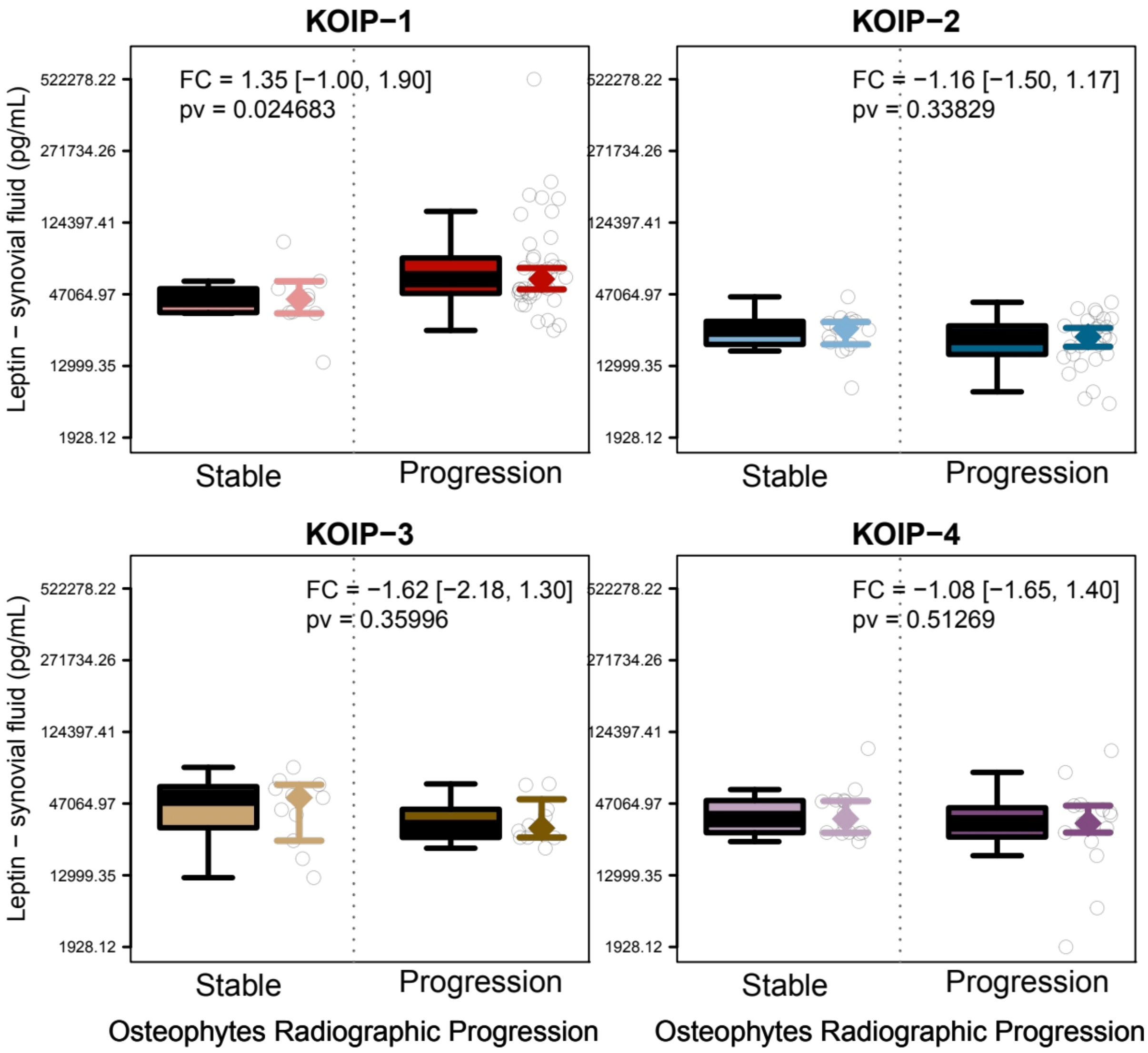

[Back to top](#)

S11: Osteophytes radiographic progression - synovial Adiponectin

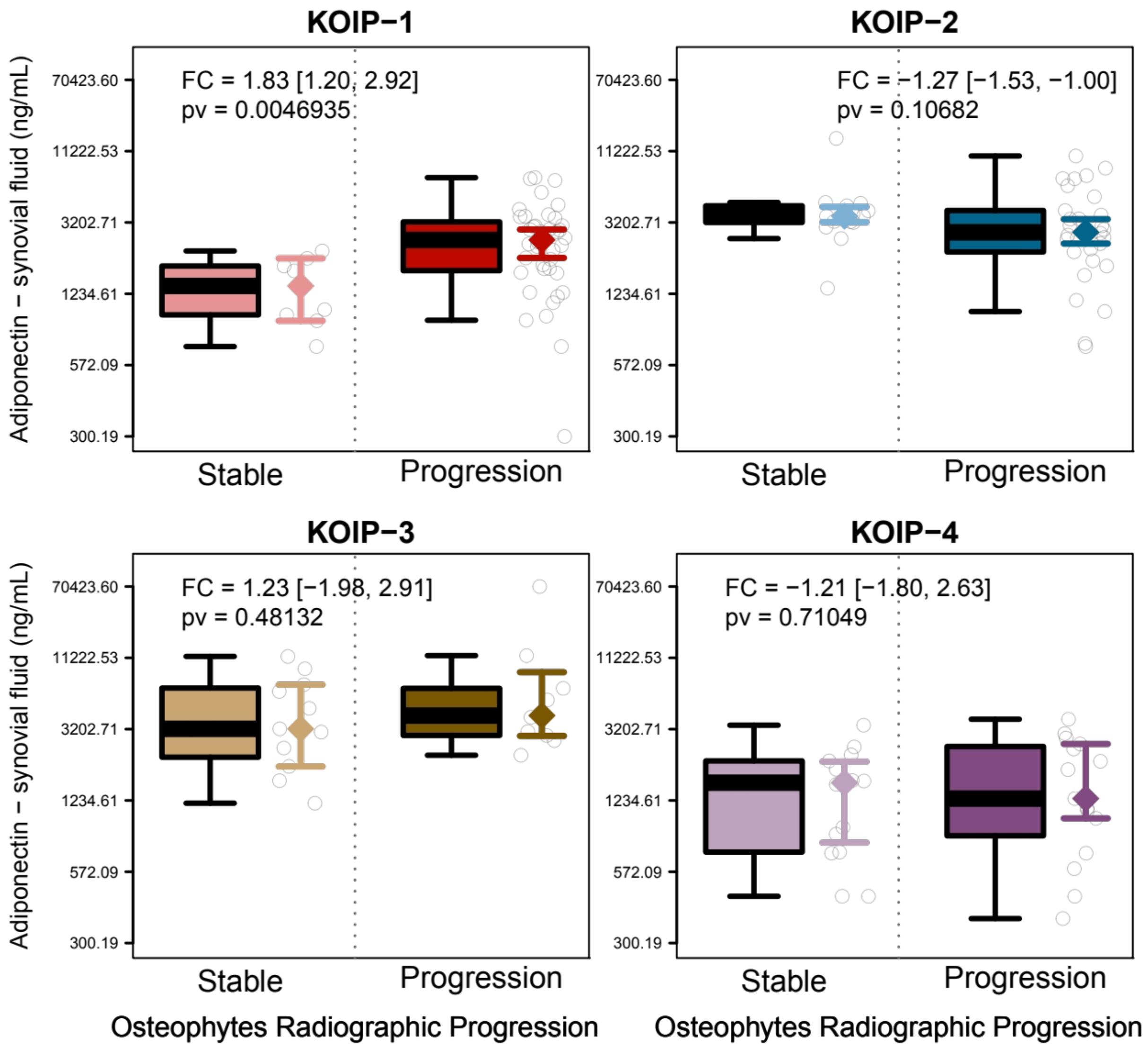

Back to top

S12: Osteophytes radiographic progression - synovial Omentin

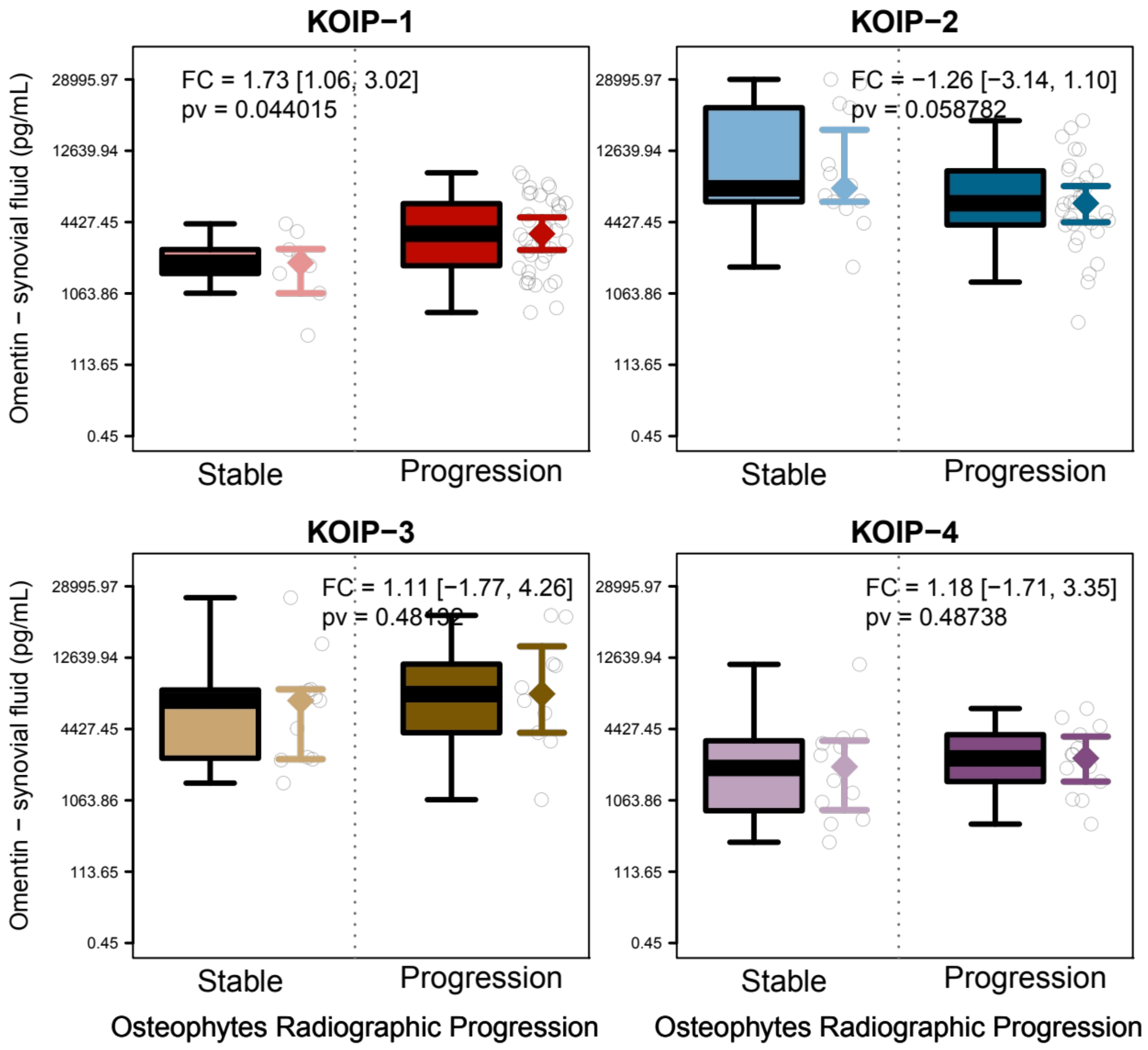

Back to top

S13: Osteophytes radiographic progression - synovial Osteopontin

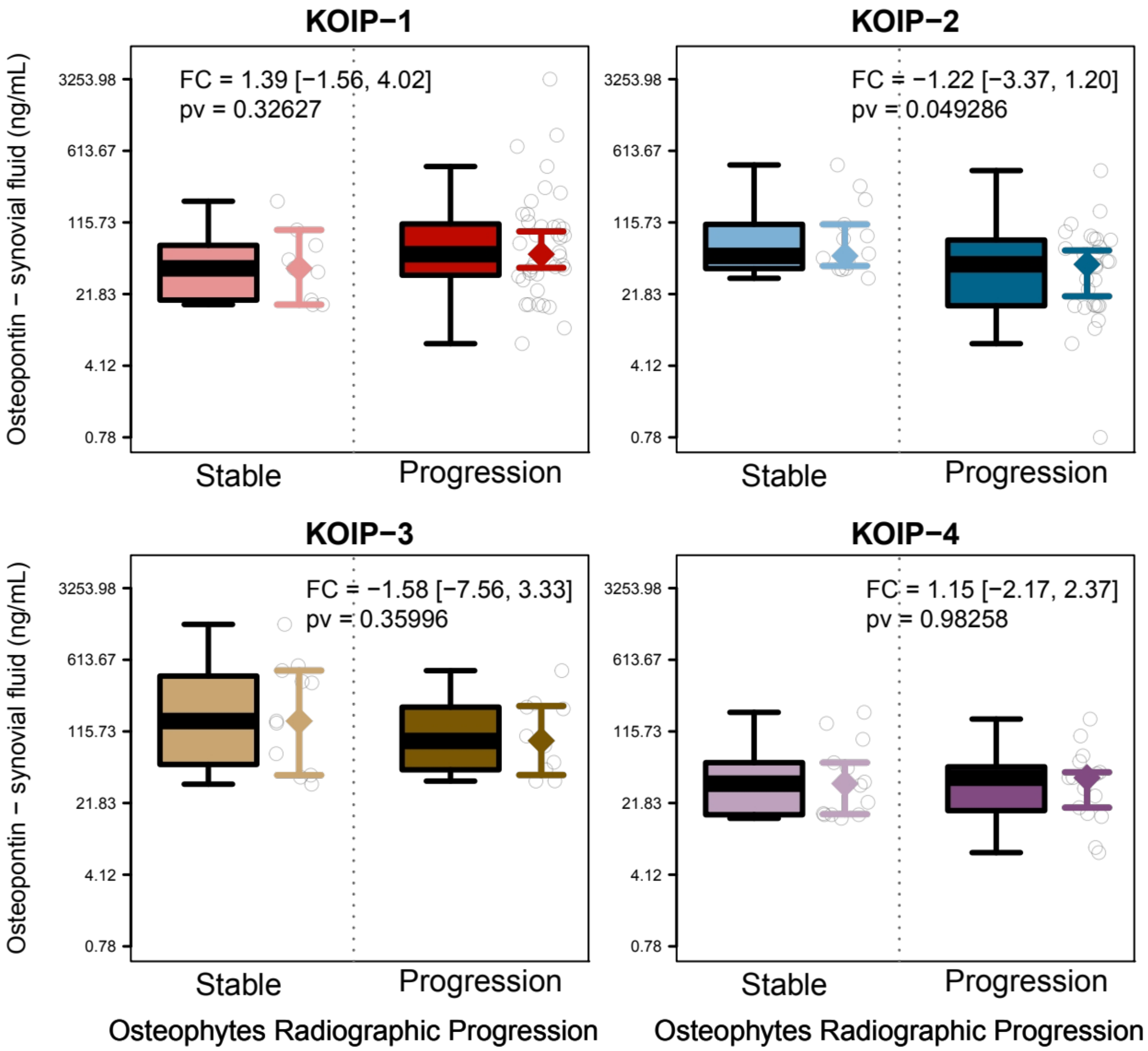

[Back to top](#)

S14: Osteophytes radiographic progression - synovial Calprotectin

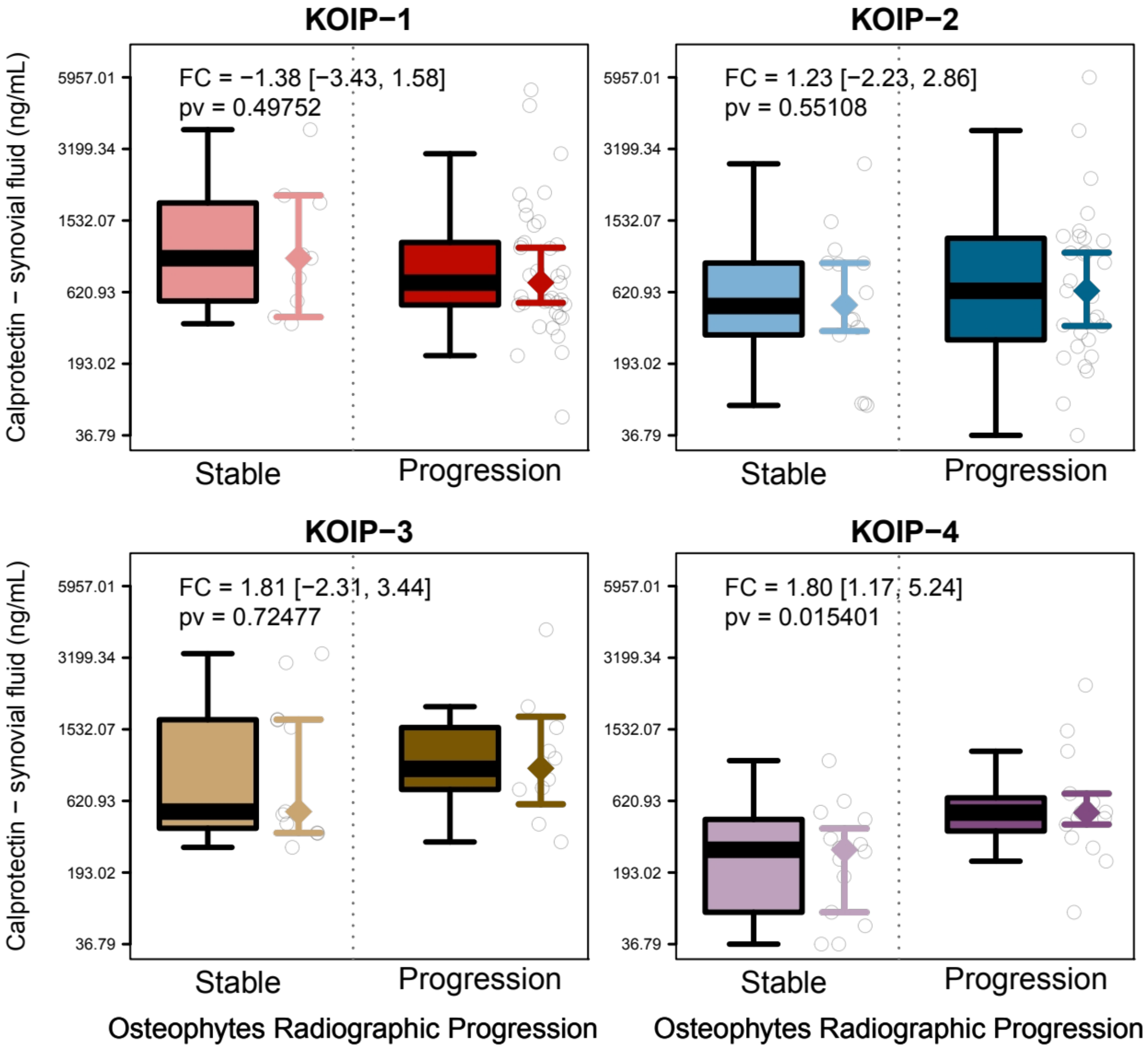

Back to top

S15: Osteophytes radiographic progression - plasma Irisin

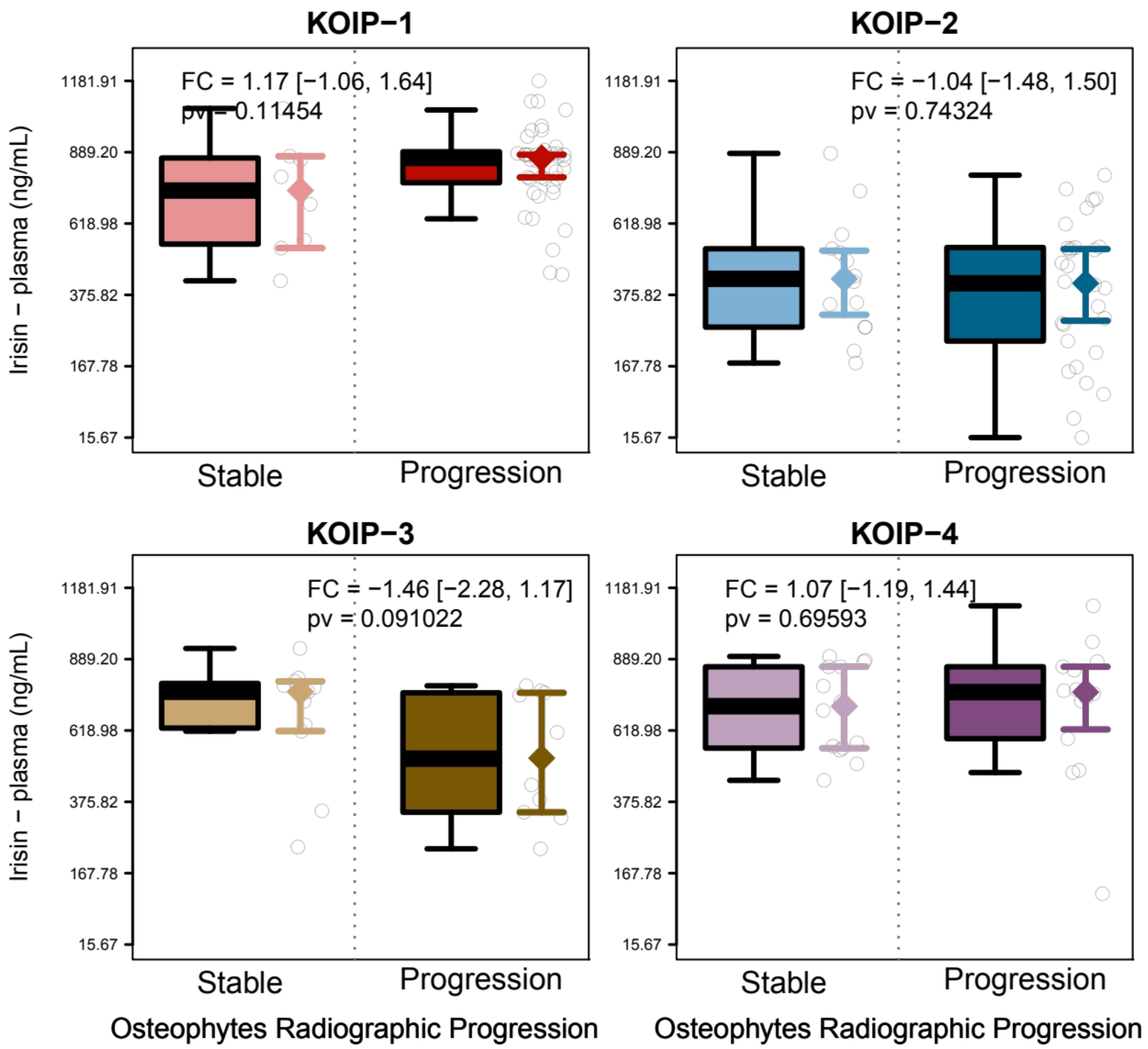

[Back to top](#)

S16: Osteophytes radiographic progression - plasma Leptin

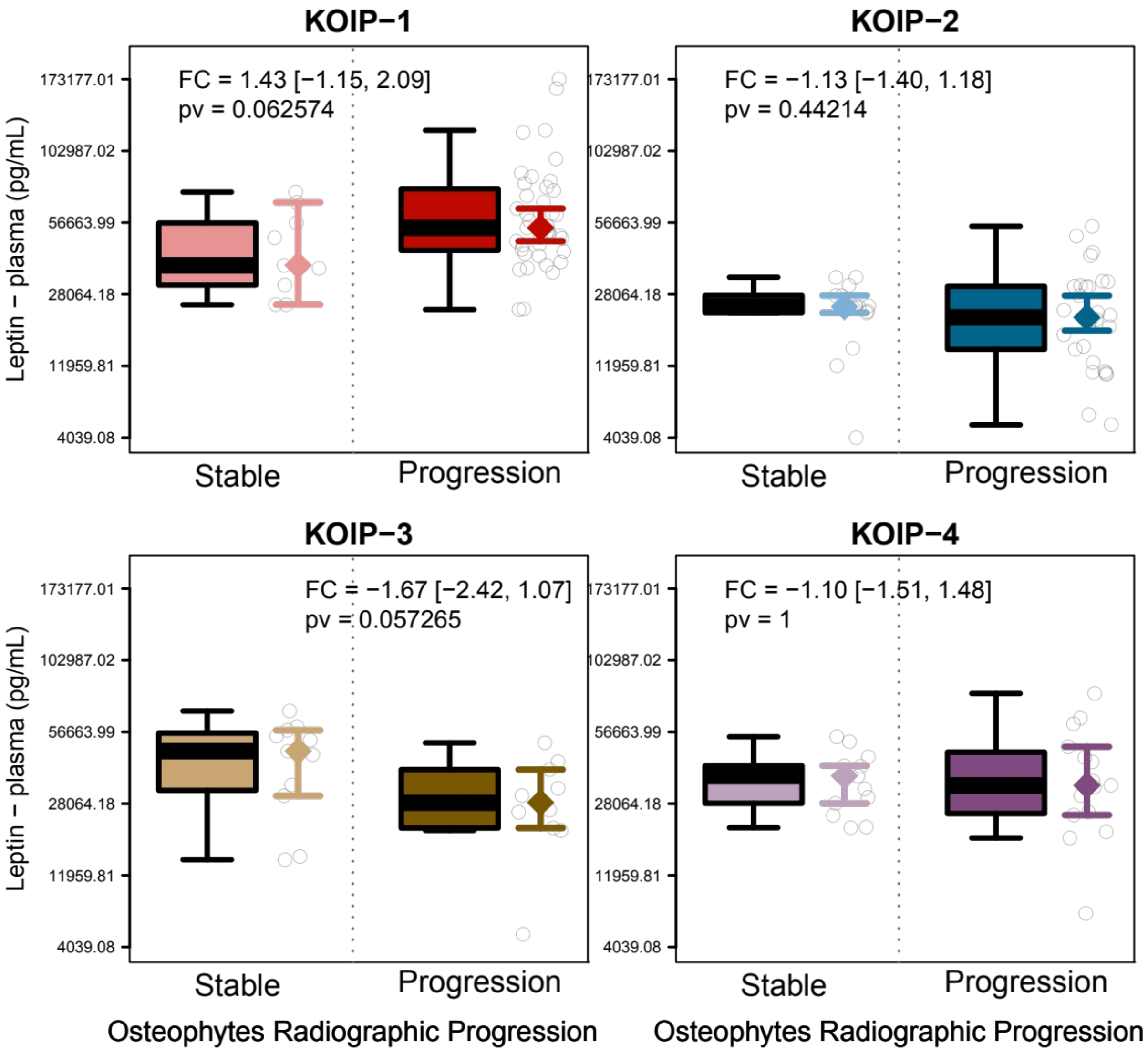

Back to top

S17: Osteophytes radiographic progression - plasma Omentin

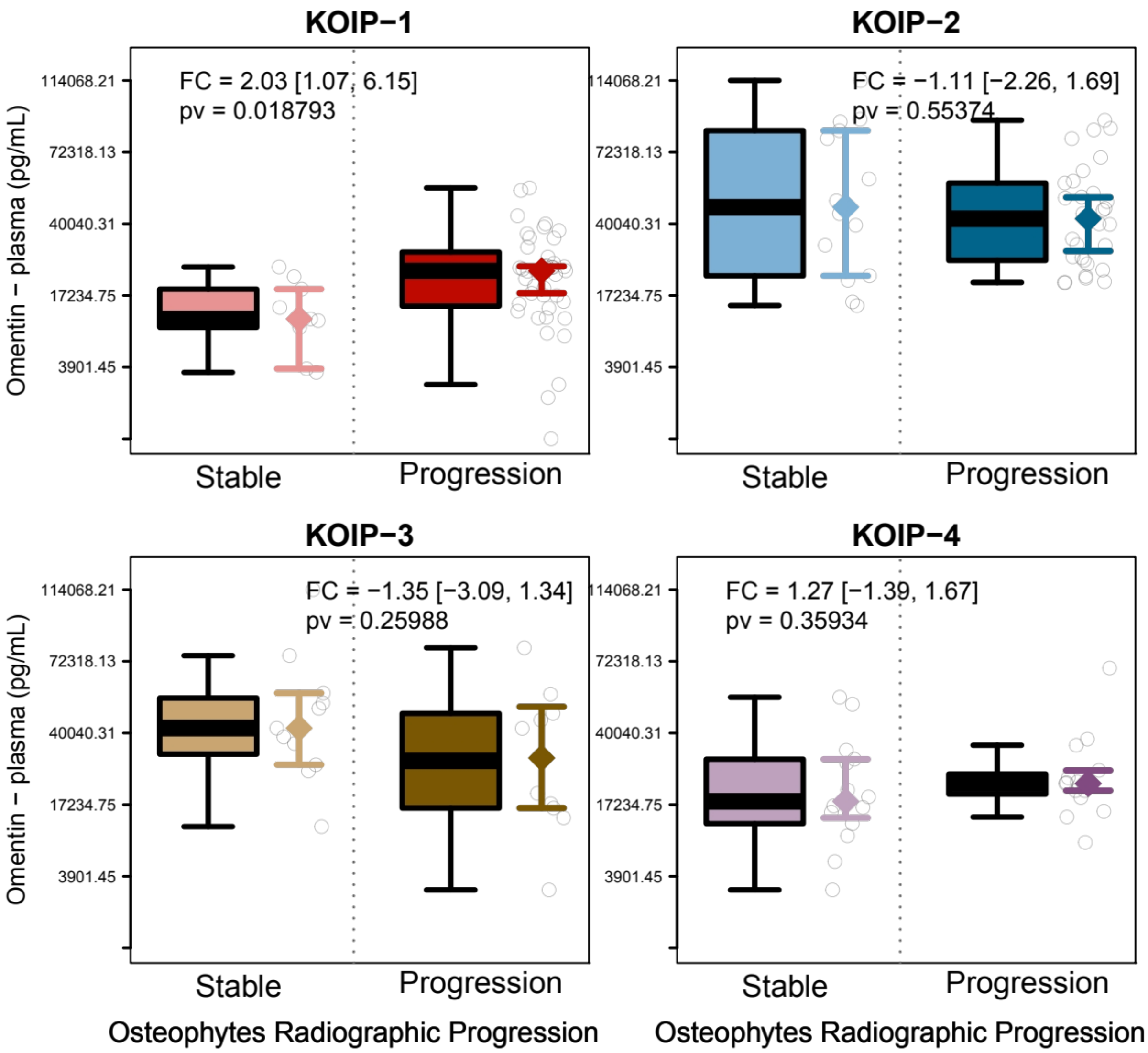

[Back to top](#)

S18: Osteophytes radiographic progression - plasma Osteopontin

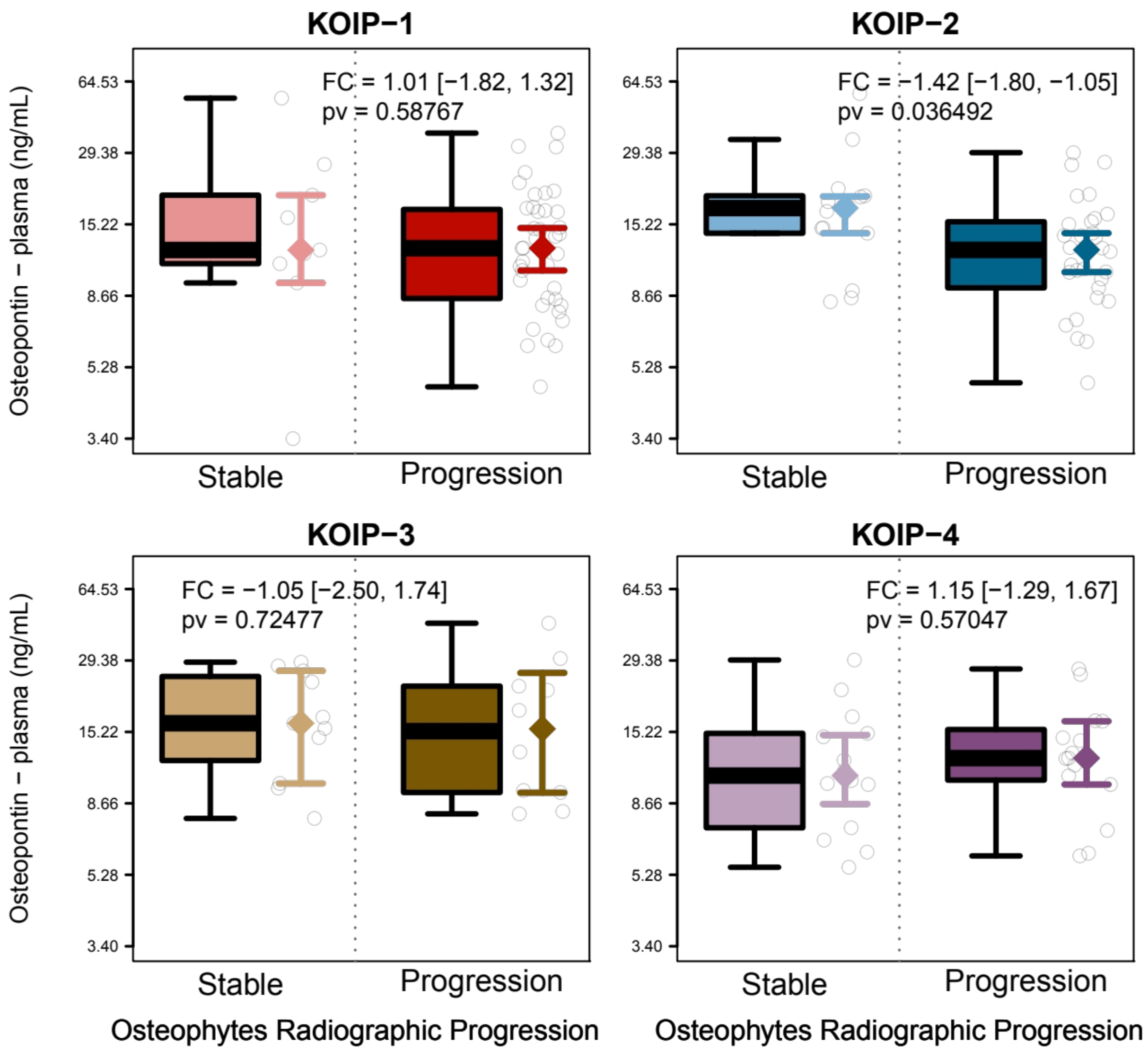

[Back to top](#)

S19: Osteophytes radiographic progression - plasma Interleukin-8

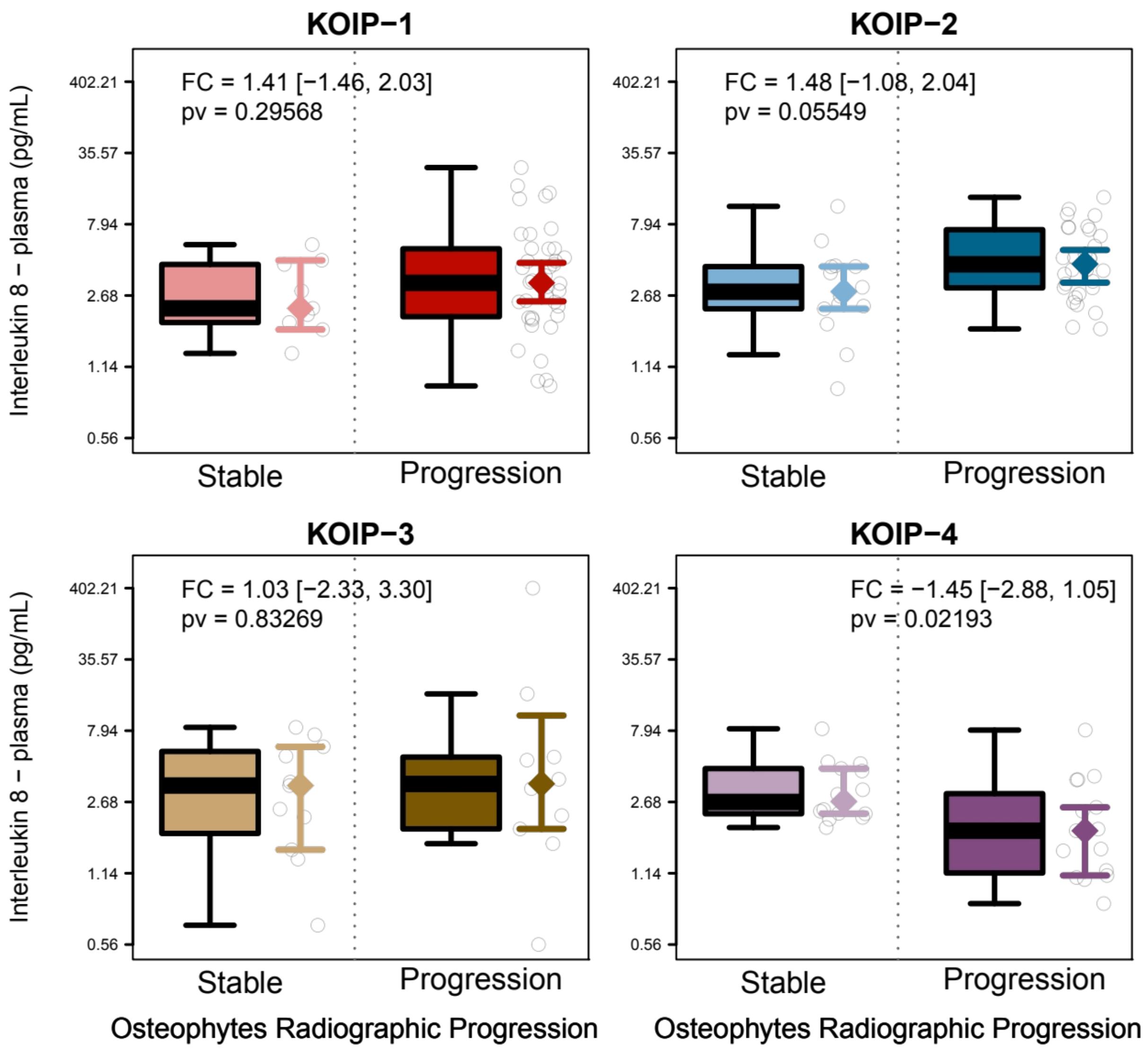

Back to top

S20: JSN radiographic progression - synovial Irisin

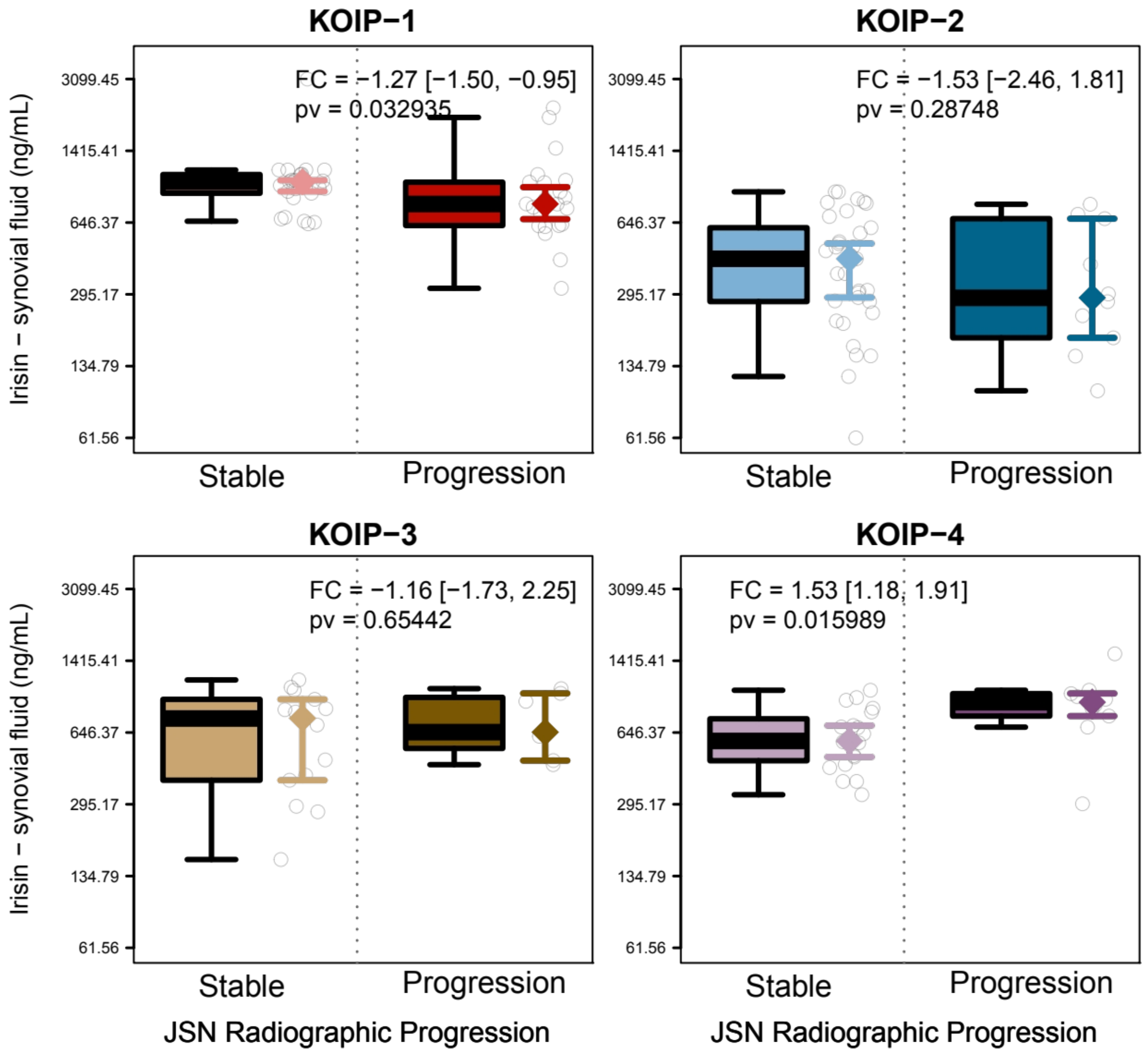

[Back to top](#)

S21: JSN radiographic progression - synovial Leptin

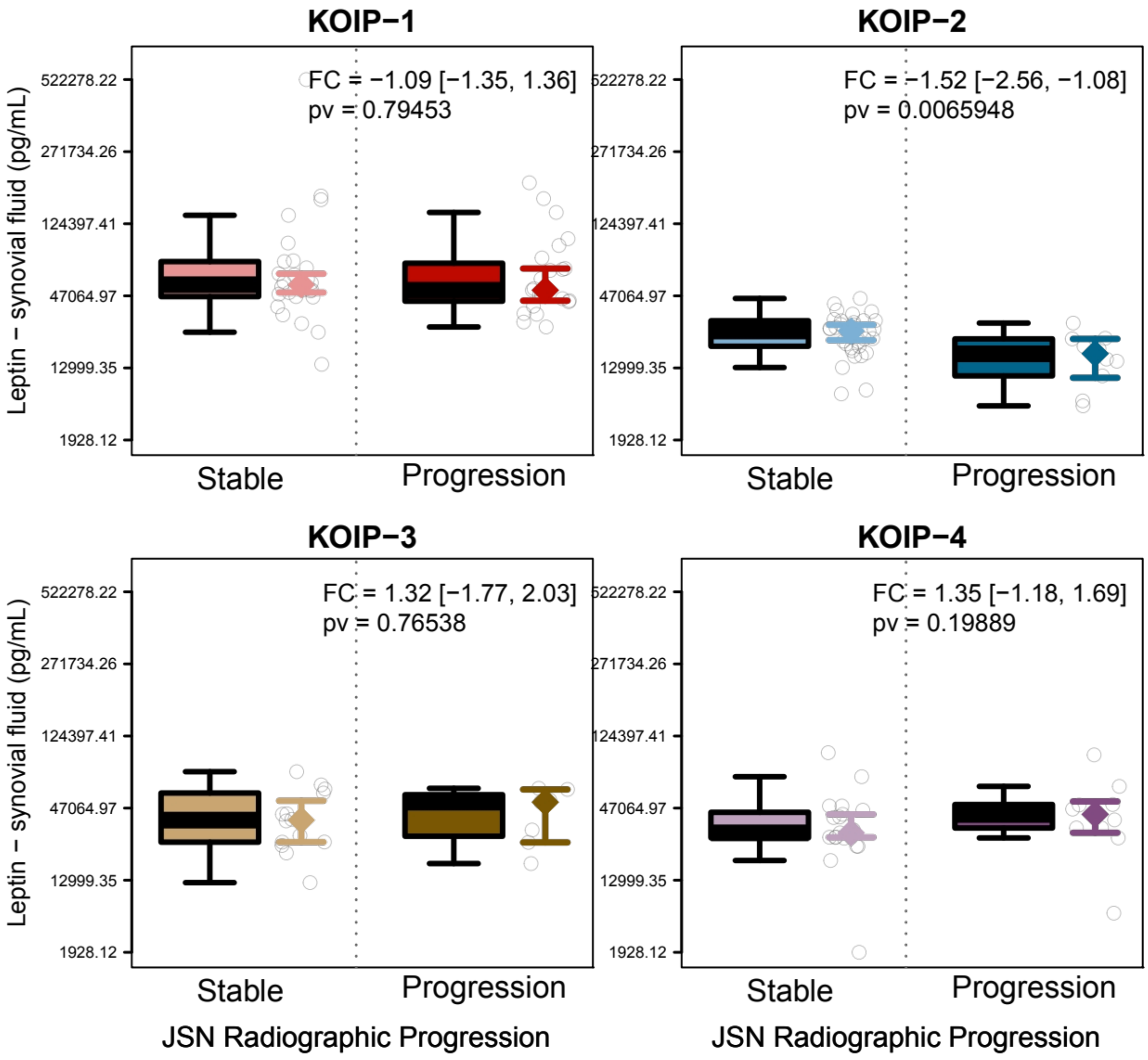

Back to top

S22: JSN radiographic progression - synovial Omentin

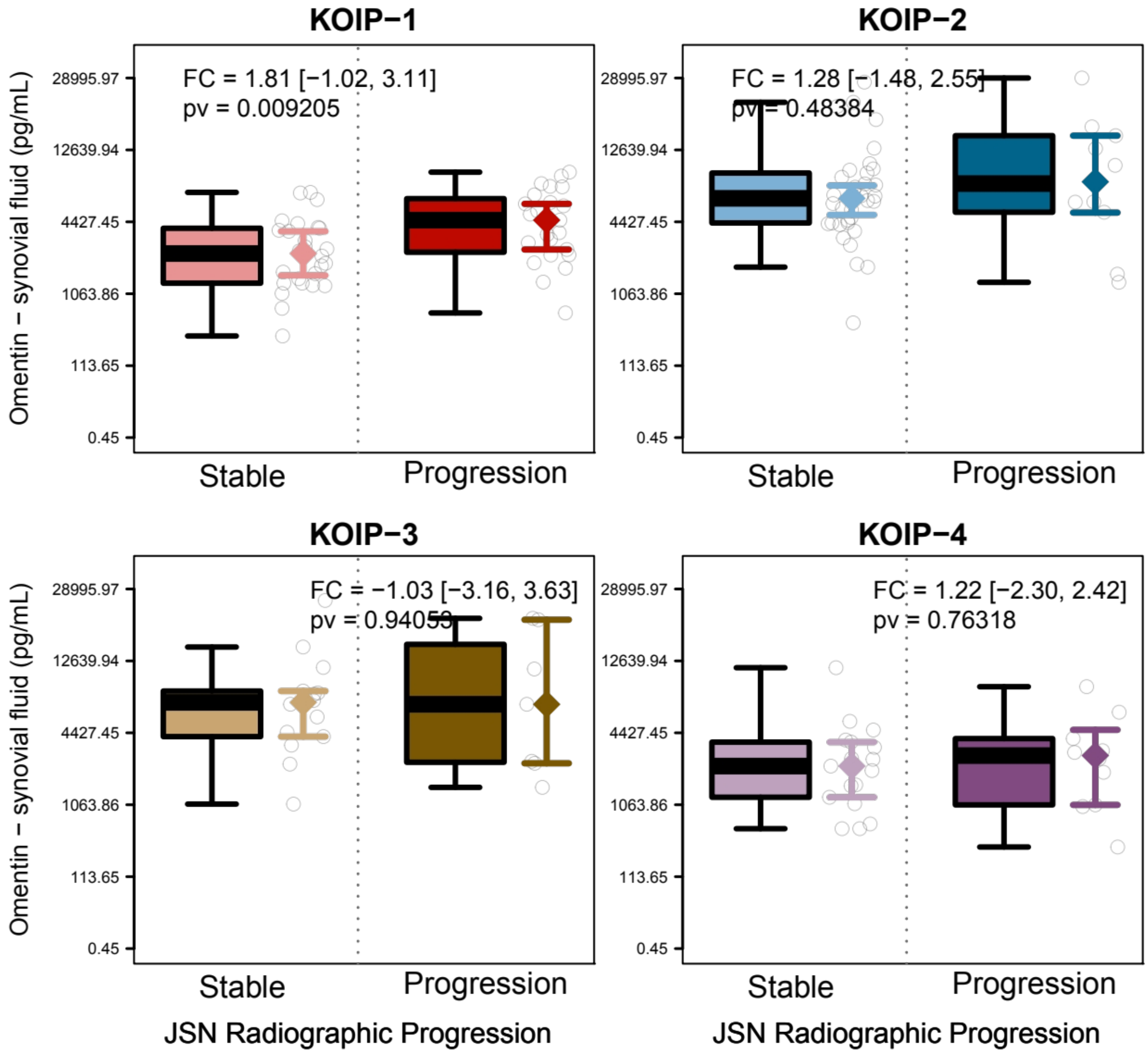

[Back to top](#)

S23: JSN radiographic progression - synovial Osteopontin

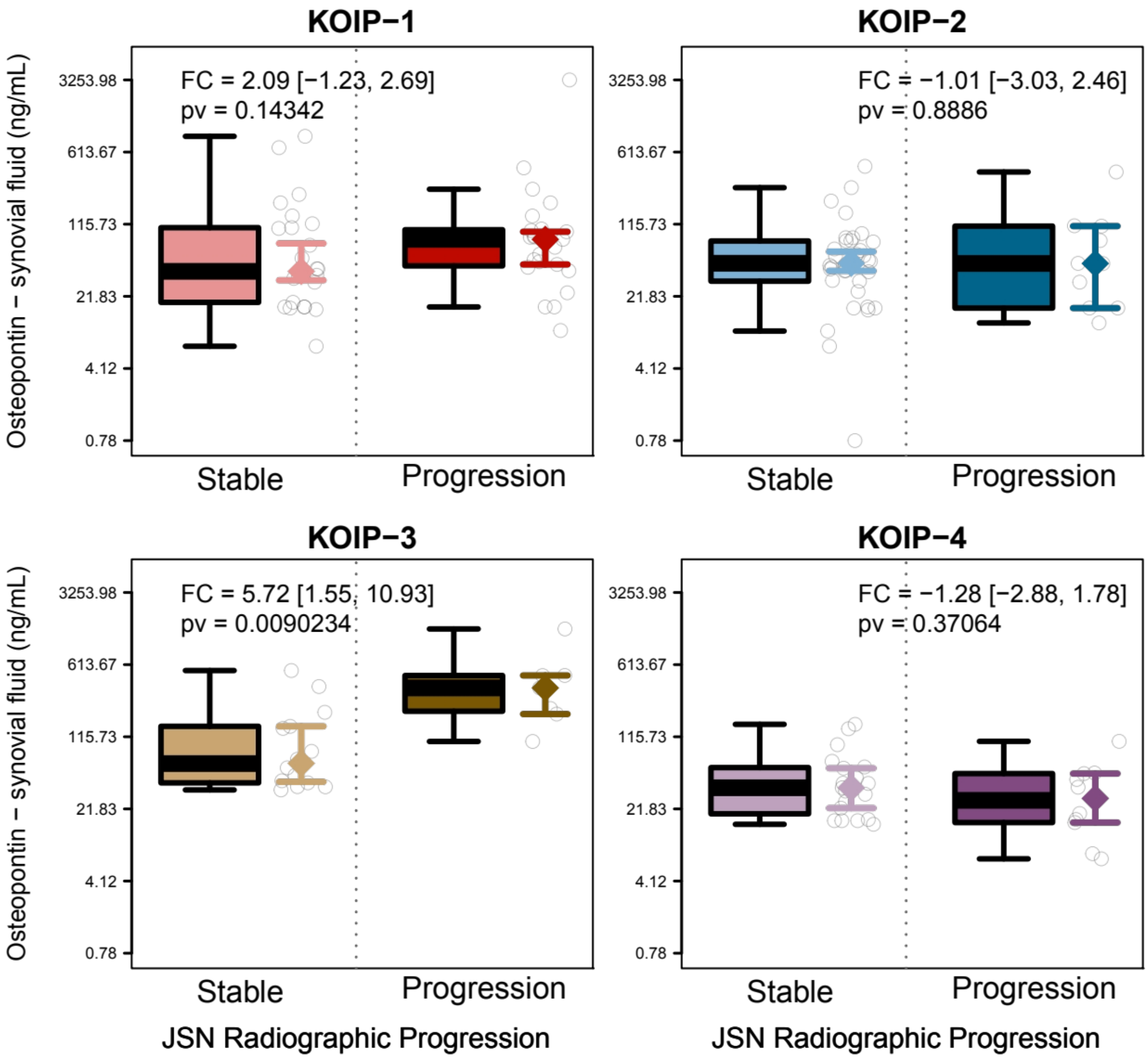

Back to top

S24: JSN radiographic progression - plasma Irisin

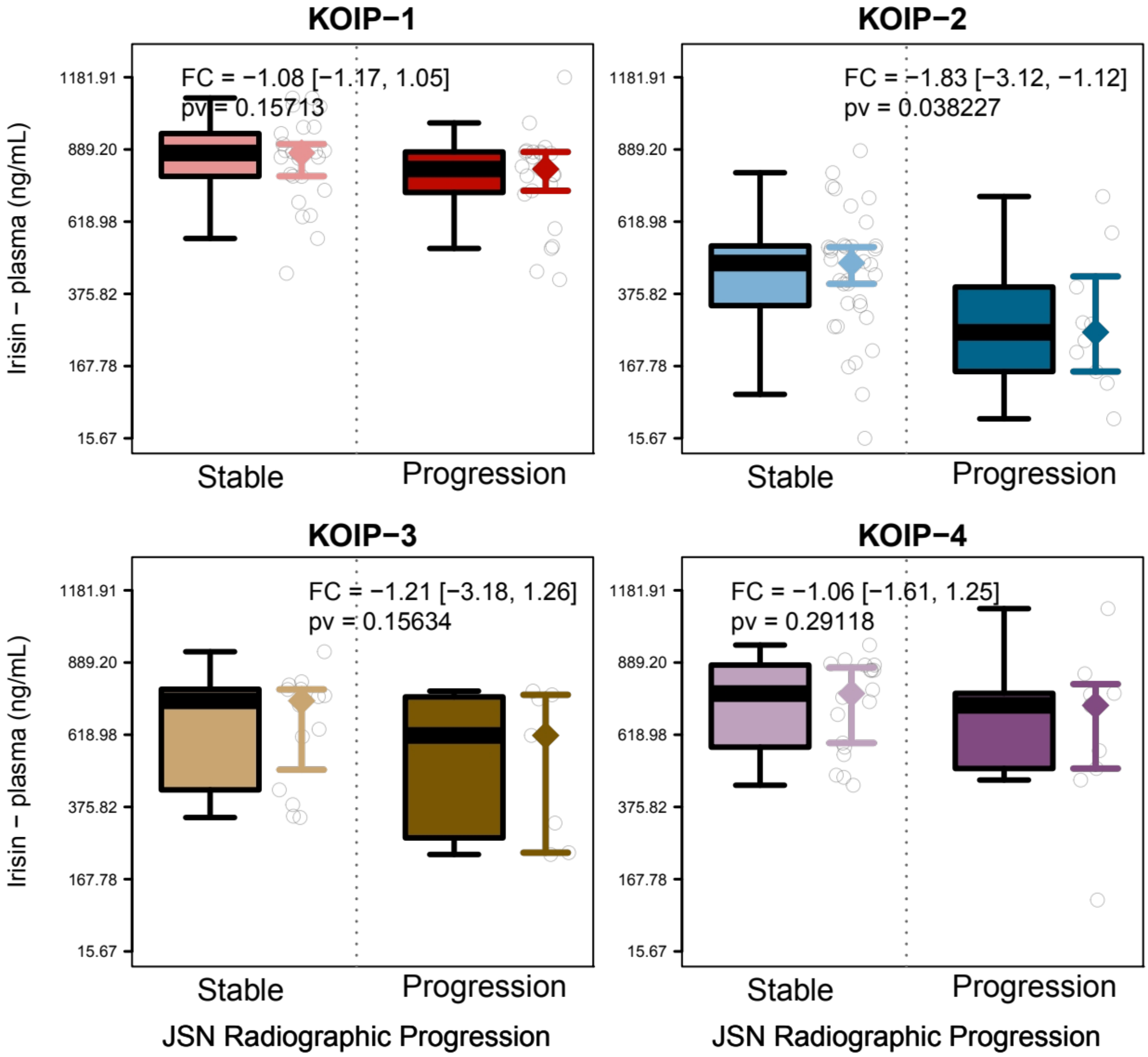

[Back to top](#)

S25: JSN radiographic progression - plasma Leptin

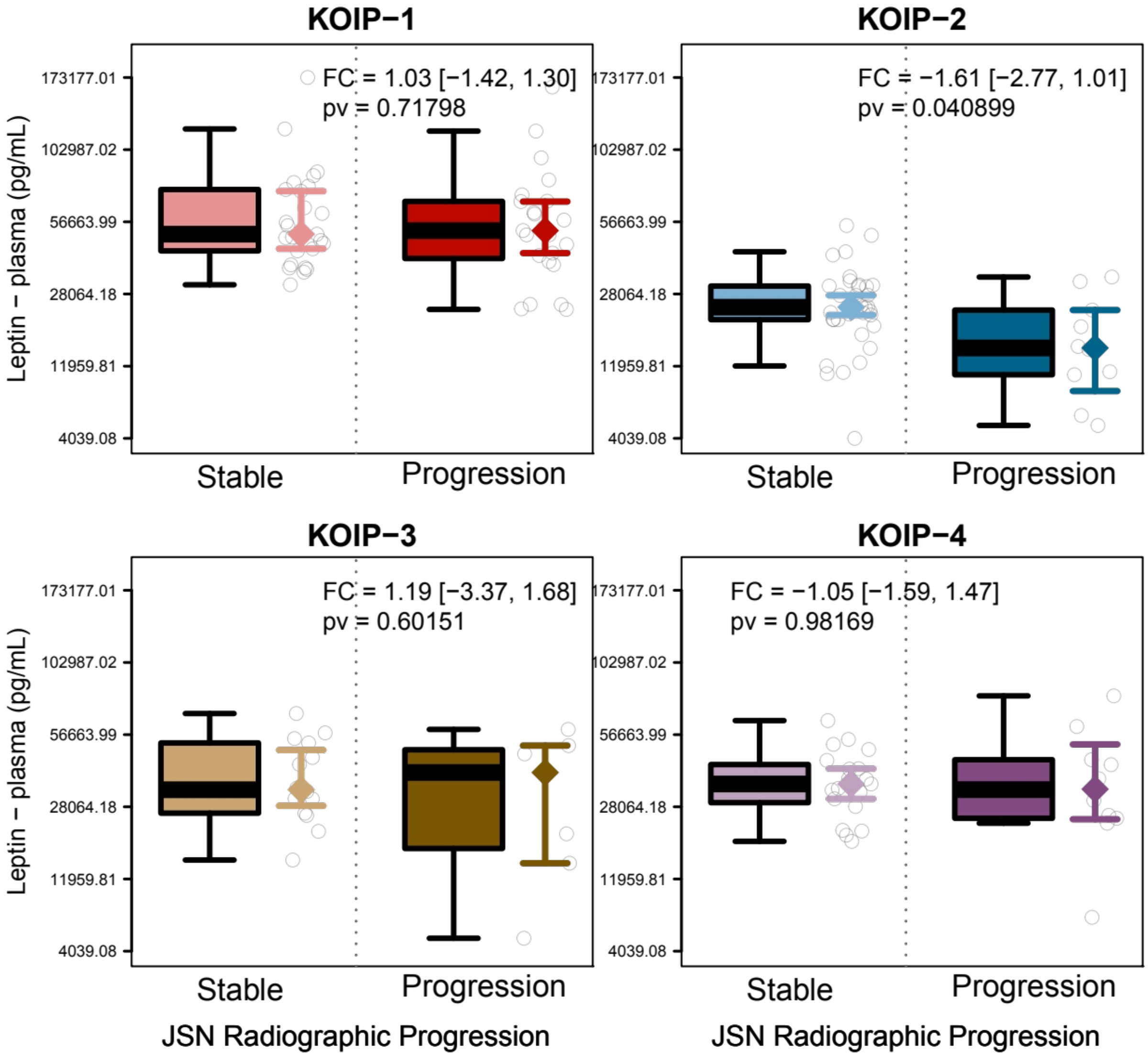

[Back to top](#)

S26: Ultrasound Joint Effusion - synovial Irisin

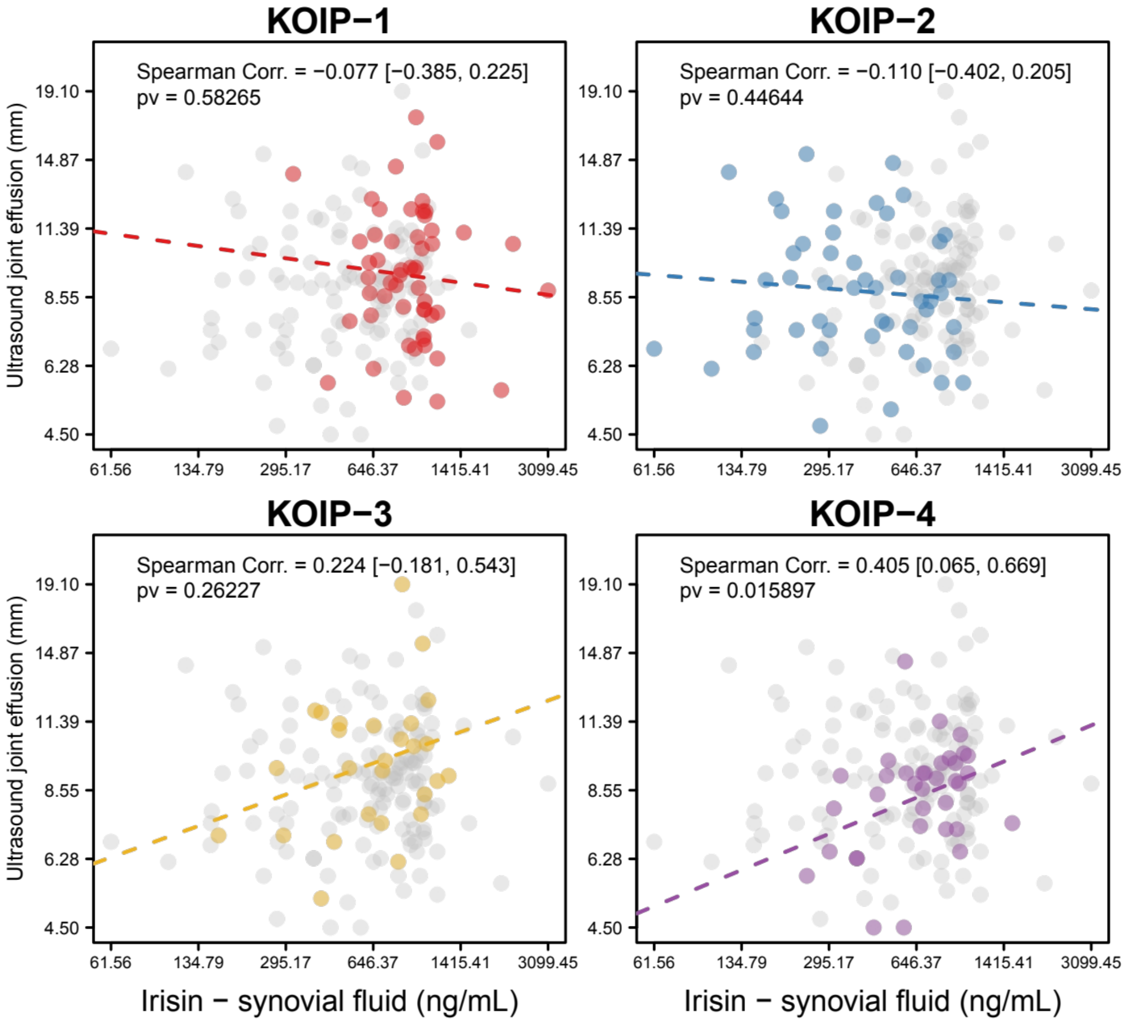

Back to top

S27: Ultrasound Joint Effusion - synovial Leptin

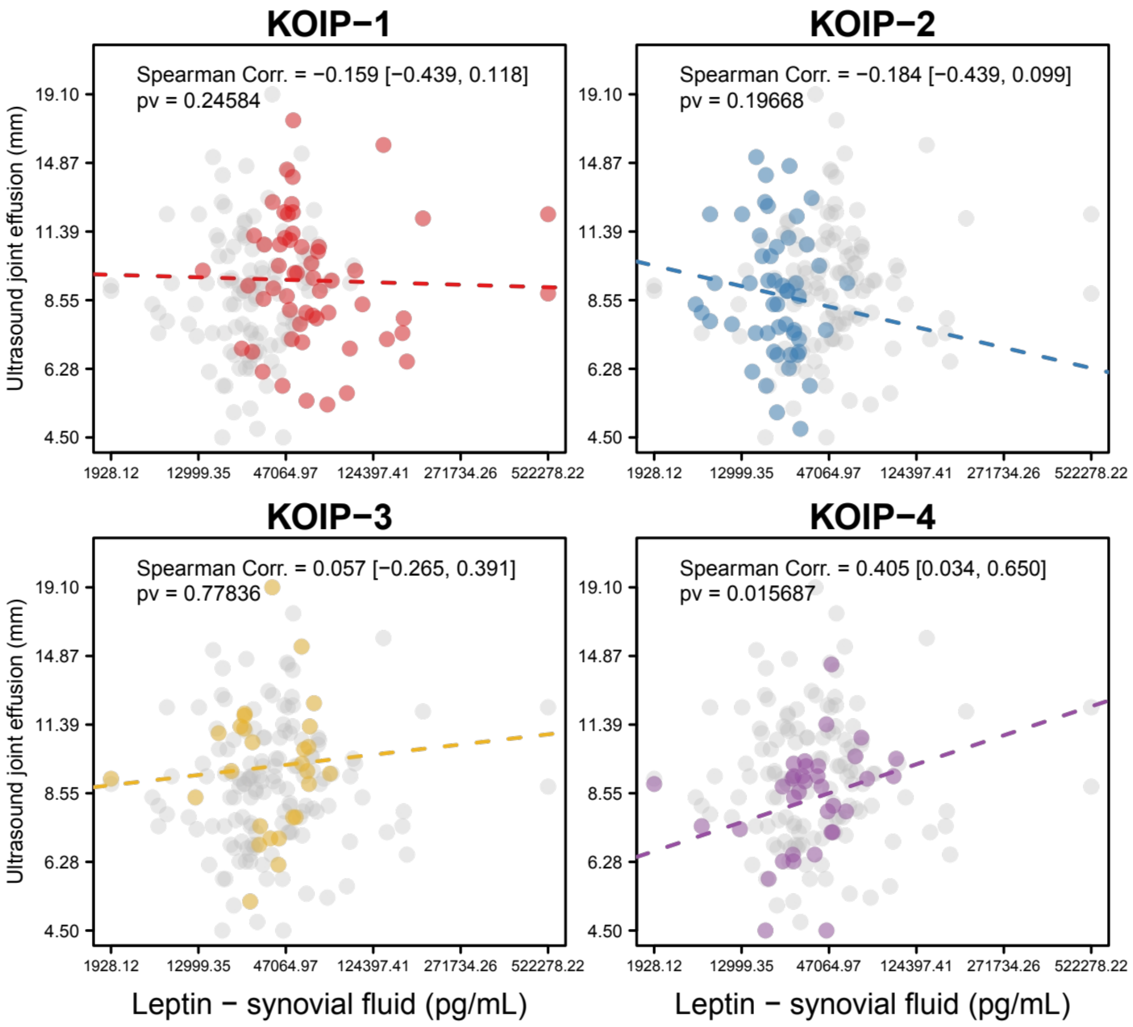

Back to top

S28: Ultrasound Joint Effusion - synovial Omentin

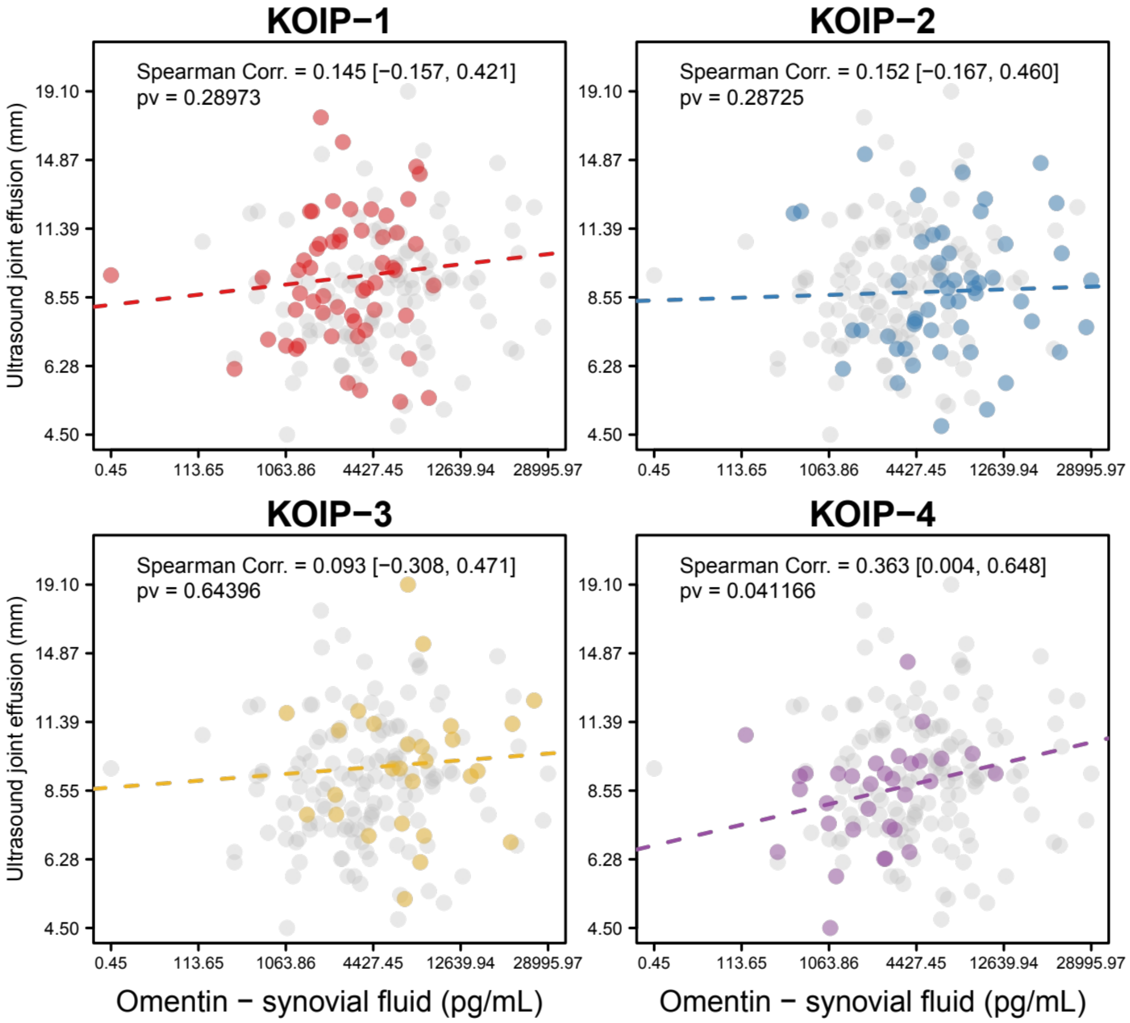

Back to top

S29: Ultrasound Joint Effusion - synovial C-Reactive Protein

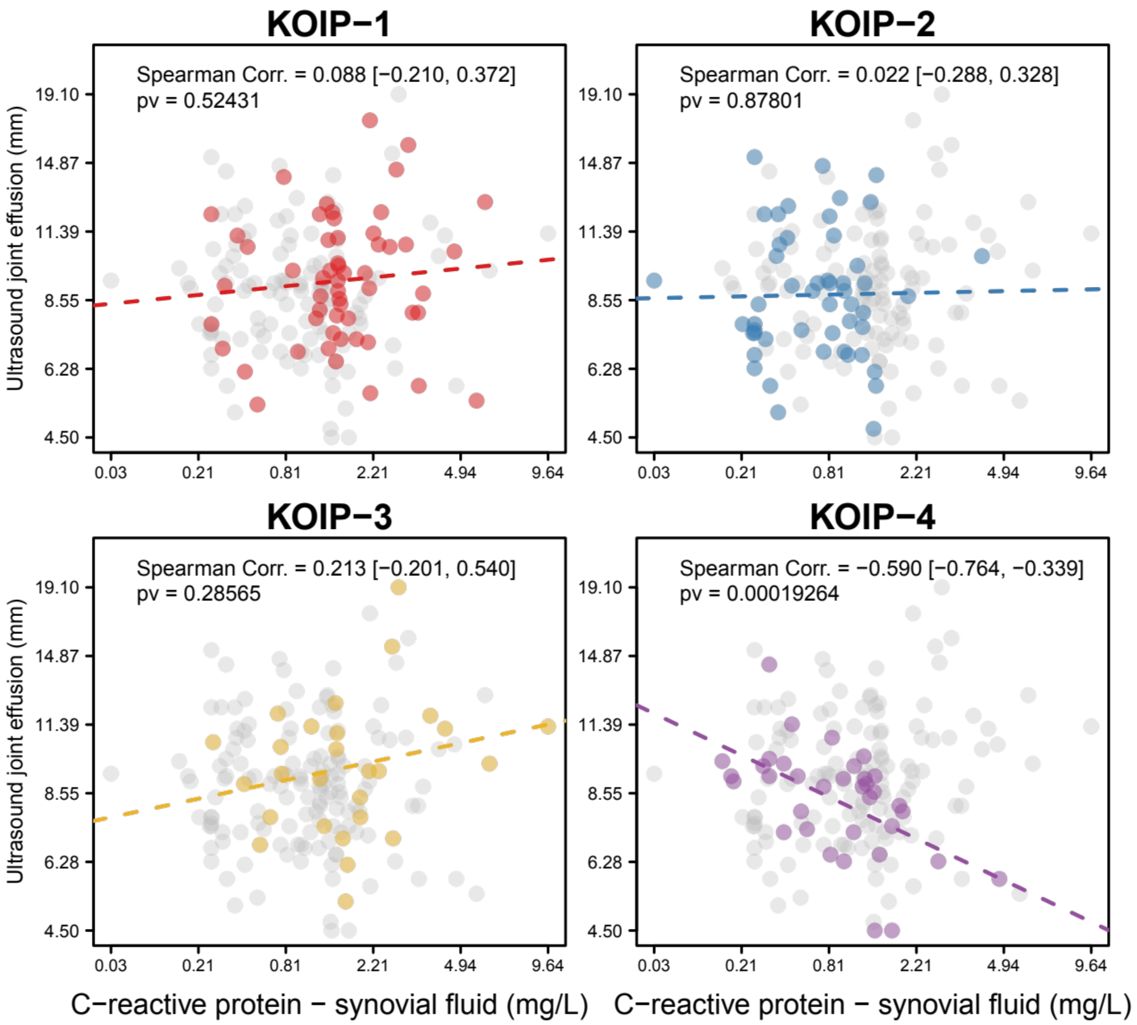

[Back to top](#)

S30: Ultrasound Joint Effusion - plasma Leptin

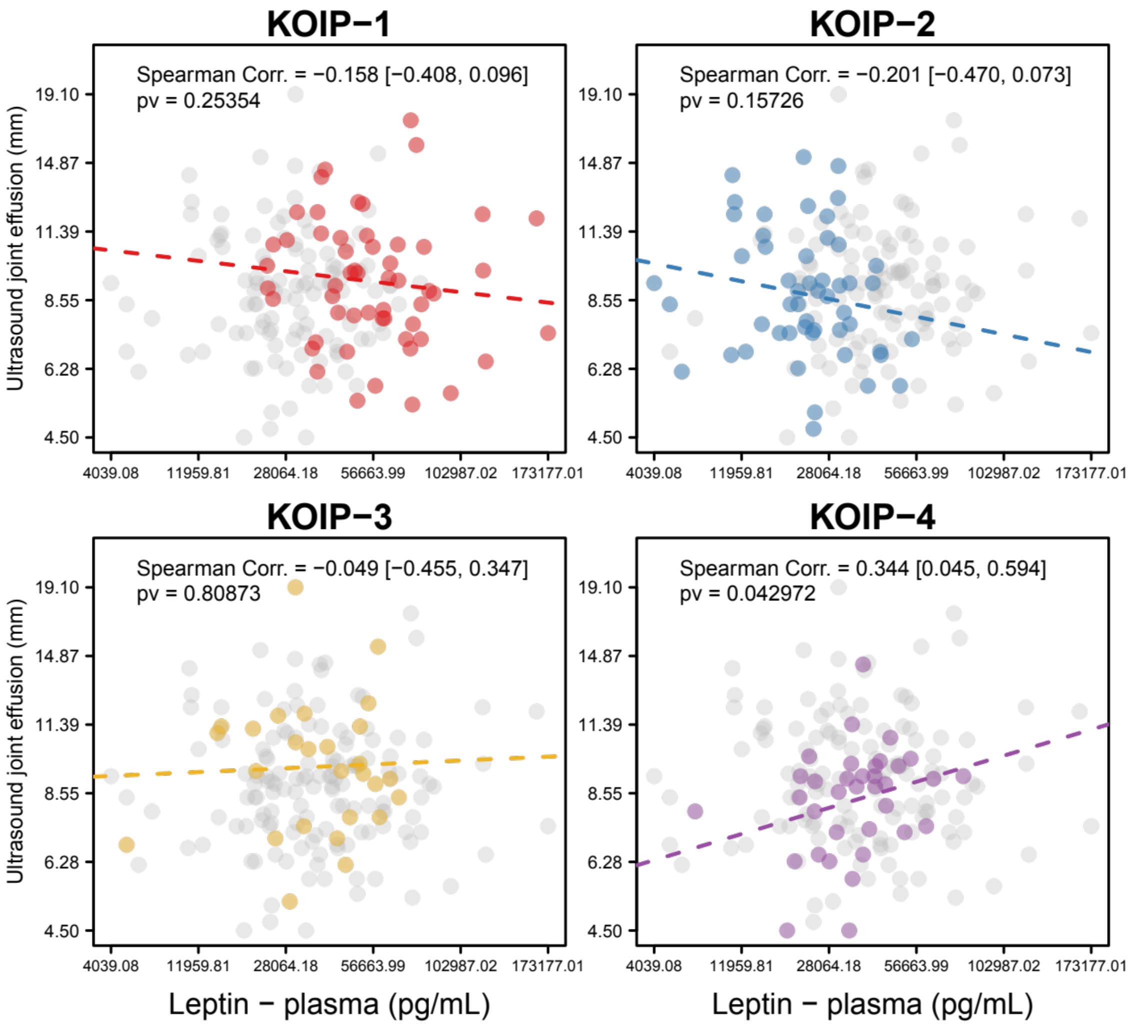

Back to top

S31: Ultrasound Joint Effusion - plasma Calprotectin

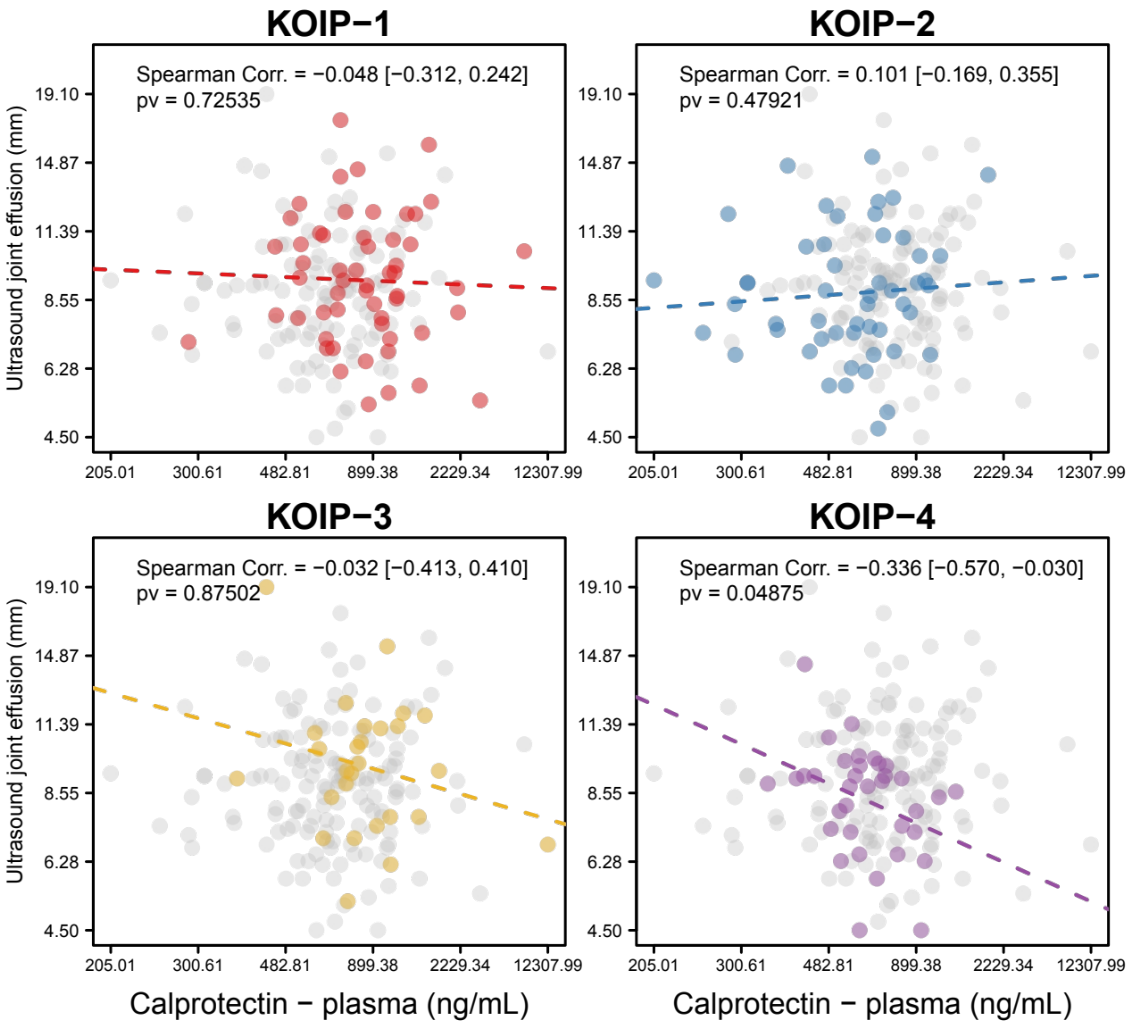

Back to top

S32: Ultrasound Joint Effusion - plasma Interleukin-6

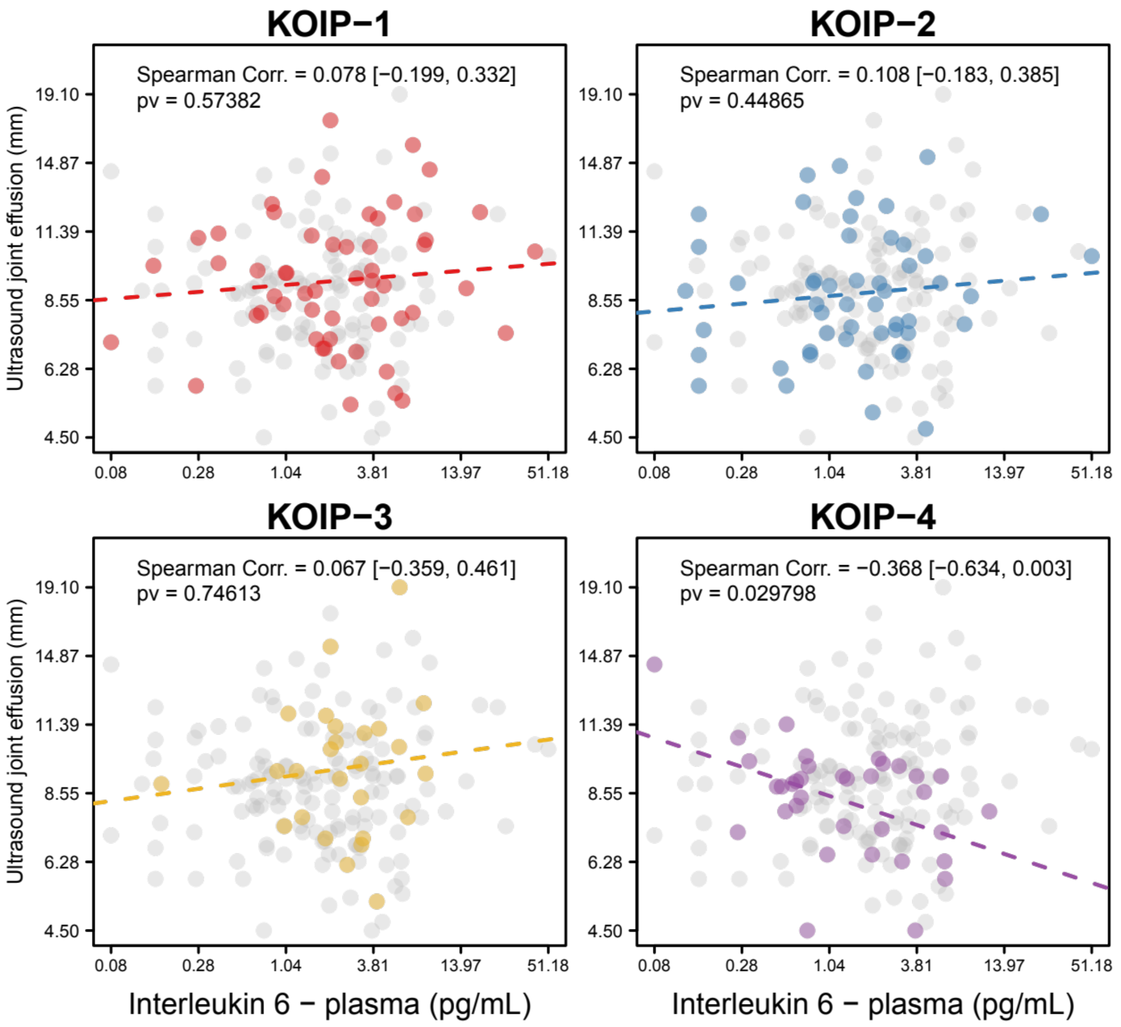

Back to top

S33: Ultrasound Joint Effusion - plasma C-reactive protein

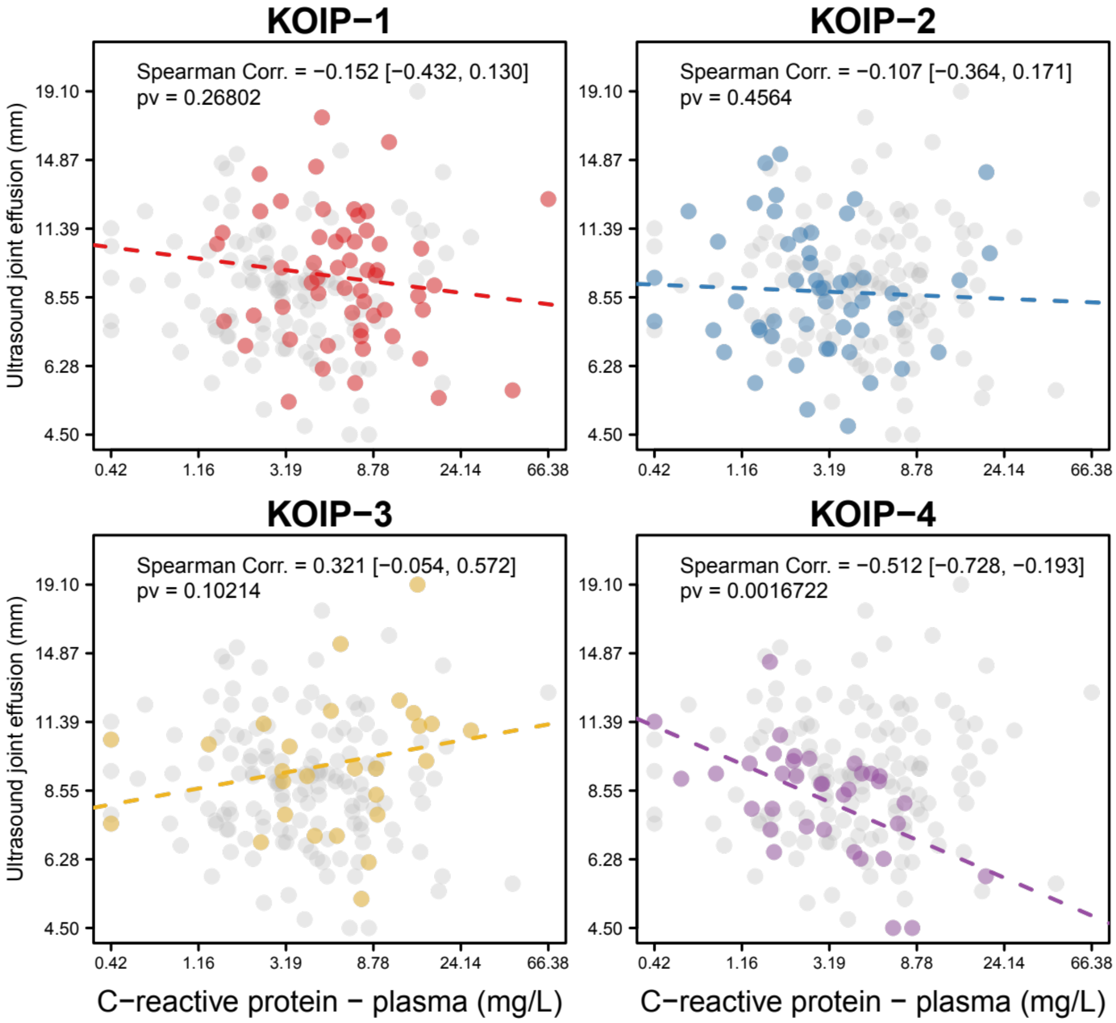

[Back to top](#)
