# Supplementary material for: Specific-cytokine associations with outcomes in knee osteoarthritis subgroups: breaking down disease heterogeneity with phenotyping
Source: Arthritis Res Ther. 2024 Jan 11;26:19. doi: 10.1186/s13075-023-03244-y (PMC10782658; doi:10.1186/s13075-023-03244-y)
Supplement: Supplementary file 2 — Additional file 2. [file 13075_2023_3244_MOESM2_ESM.pdf]

Supplementary Table S1. Cytokines quantifications in the patients series by Knee Osteoarthritis Inflammatory Phenotypes (**KOIP**). Cells show medians and median absolute deviations (continuous) and absolute frequencies and percentages (categorical) for the 13 cytokines evaluated in plasma and synovial fluid samples of the patients' cohort. Statistical significance was assessed using a Kruskal-Wallis (continuous) or a Fisher's test for contingency tables (categorical variables). **KOIP:** Knee Osteoarthritis Inflammatory Phenotype.

		**All 168 (100%)**	**KOIP-1 55 (32.7%)**	**KOIP-2 51 (30.4%)**	**KOIP-3 27 (16.1%)**	**KOIP-4 35 (20.8%)**	**P-value**
**SYNOVIAL FLUID**	**C-reactive protein (mg/L)**	1.23 [0.03, 9.64]	1.52 [0.27, 6.02]	0.78 [0.03, 4.10]	1.61 [0.27, 9.64]	0.98 [0.15, 4.76]	< 0.0001
	**Tumor Necrosis Factor alpha (pg/mL)**	9.02 [1.19, 127.49]	9.09 [1.19, 127.49]	8.14 [2.00, 18.17]	9.90 [2.19, 22.16]	8.90 [1.78, 17.98]	0.1239
	**Interleukin 6 (pg/mL)**	116.33 [0.11, 15683.26]	208.03 [7.55, 15683.26]	91.67 [0.11, 1857.83]	222.45 [22.77, 5820.23]	71.56 [4.01, 652.84]	0.0012
	**Interleukin 8 (pg/mL)**	6.21 [0.04, 881.40]	8.00 [0.44, 881.40]	6.21 [0.11, 278.44]	8.21 [0.04, 145.52]	3.92 [0.44, 29.02]	0.0012
	**Nerve Growth Factor (pg/mL)**	2.26 [0.01, 21.63]	2.26 [0.01, 21.63]	2.26 [0.46, 8.25]	2.20 [0.46, 6.90]	2.20 [0.72, 12.17]	0.038
	**Calprotectin (ng/mL)**	576.06 [36.79, 5957.01]	763.04 [60.31, 5376.84]	599.99 [36.79, 5957.01]	759.60 [197.72, 4129.89]	420.07 [36.79, 2447.75]	0.0006
	**Leptin (pg/mL)**	36160.71 [1928.12, 522278.22]	54016.11 [14014.43, 522278.22]	23126.79 [5335.19, 58808.60]	39506.85 [1928.12, 79207.03]	34888.07 [1928.12, 101330.36]	< 0.0001
	**Irisin (ng/mL)**	695.95 [61.56, 3099.45]	938.23 [315.26, 3099.45]	422.07 [61.56, 983.08]	701.88 [161.47, 1270.77]	686.28 [242.12, 1527.24]	< 0.0001
	**Adiponectin (ng/mL)**	2420.01 [300.19, 70423.60]	1937.73 [300.19, 6721.39]	3047.42 [689.12, 14901.06]	3372.91 [1194.76, 70423.60]	1501.12 [370.24, 3730.72]	< 0.0001
	**Omentin (pg/mL)**	4292.68 [0.45, 28995.97]	3073.42 [0.45, 9401.45]	6648.21 [496.86, 28995.97]	6943.02 [1081.05, 25688.53]	2787.84 [130.78, 11575.06]	< 0.0001
	**Osteopontin (ng/mL)**	47.95 [0.78, 3253.98]	48.55 [6.88, 3253.98]	46.75 [0.78, 441.11]	103.82 [15.18, 1400.55]	36.02 [6.88, 180.41]	0.0013
	**Visfatin (ng/mL)**	2.07 [0.13, 553.81]	2.28 [0.49, 70.61]	1.88 [0.13, 553.81]	2.11 [0.42, 7.53]	1.90 [0.53, 12.96]	0.5075
	**Resistin (pg/mL)**	1480.20 [32.46, 53485.59]	1692.08 [83.70, 53485.59]	1390.64 [52.92, 10386.82]	1242.23 [83.70, 18547.89]	1329.84 [32.46, 14574.82]	0.421
**PLASMA**	**C-reactive protein (mg/L)**	4.26 [0.42, 66.38]	6.87 [1.44, 66.38]	2.59 [0.42, 20.38]	6.00 [0.42, 27.13]	2.90 [0.42, 19.50]	< 0.0001
	**Interleukin 6 (pg/mL)**	2.01 [0.08, 51.18]	2.28 [0.08, 42.02]	1.55 [0.12, 51.18]	2.45 [0.16, 8.28]	1.27 [0.08, 11.19]	0.0149
	**Interleukin 8 (pg/mL)**	3.19 [0.56, 402.21]	3.21 [0.93, 24.91]	3.79 [0.91, 13.13]	3.52 [0.56, 402.21]	2.30 [0.83, 8.22]	0.0152
	**Tumor Necrosis Factor alpha (pg/mL)**	6.37 [1.77, 22.13]	7.18 [1.77, 22.13]	6.18 [2.94, 14.66]	6.70 [2.59, 10.74]	6.08 [2.63, 8.86]	0.0997
	**Nerve Growth Factor (pg/mL)**	1.52 [0.22, 14.81]	1.52 [0.40, 14.81]	1.52 [0.22, 5.19]	1.69 [0.40, 8.31]	1.52 [0.44, 6.52]	0.2699
	**Calprotectin (ng/mL)**	694.27 [205.01, 12307.99]	851.11 [287.17, 6607.95]	613.63 [205.01, 1837.92]	800.12 [365.98, 12307.99]	588.54 [342.92, 1291.50]	< 0.0001
	**Leptin (pg/mL)**	36688.11 [4039.08, 173177.01]	54392.71 [23739.58, 173177.01]	23566.59 [4039.08, 54817.52]	39809.19 [5017.56, 68130.76]	35530.72 [6972.63, 78971.55]	< 0.0001
	**Irisin (ng/mL)**	707.78 [15.67, 1181.91]	859.25 [421.34, 1181.91]	437.22 [15.67, 884.41]	727.20 [234.19, 1059.97]	726.02 [116.43, 1105.78]	< 0.0001
	**Adiponectin (ng/mL)**	16406.09 [1450.48, 59716.25]	11544.55 [1450.48, 52203.90]	22357.78 [10162.63, 59716.25]	21707.37 [7771.73, 40371.01]	11562.39 [4997.58, 42515.20]	< 0.0001
	**Omentin (pg/mL)**	26859.10 [40.42, 114068.21]	22317.93 [40.42, 55037.25]	44069.15 [2444.37, 114068.21]	41837.49 [2444.37, 114068.21]	22449.96 [2444.37, 68850.68]	< 0.0001
	**Osteopontin (ng/mL)**	13.71 [3.40, 64.53]	12.52 [3.40, 64.53]	14.12 [4.78, 55.83]	16.40 [4.91, 43.44]	12.10 [5.55, 29.52]	0.1872
	**Visfatin (ng/mL)**	4.01 [2.20, 12.58]	4.02 [2.20, 12.58]	4.23 [2.42, 8.37]	4.01 [2.59, 6.04]	3.74 [2.26, 6.93]	0.281
	**Resistin (pg/mL)**	2115.23 [791.02, 10079.62]	2389.10 [791.02, 8829.96]	1891.16 [958.55, 4435.30]	2657.56 [1045.95, 10079.62]	1874.89 [1194.29, 3812.08]	0.0001

Supplementary Table S2. Cytokines and Knee Osteoarthritis (**KOA**) outcomes evaluated in the patients series. g: parameter value for a Tukey ladder of powers transformation applied to continuous variables (when necessary) to symmetrize their distribution and make them more suitable for graphical representation and linear models for ELISA batch correction.

Set	Variable type	Variable (units / values)	Type (transformation parameter)
Cytokines	Inflammation markers - synovial fluid	C-reactive protein - synovial fluid (mg/L)	Continuous (g = 0.25)
		Interleukin 6 - synovial fluid (pg/mL)	Continuous (g = 0)
		Interleukin 8 - synovial fluid (pg/mL)	Continuous (g = 0)
		Tumor Necrosis Factor alpha - synovial fluid (pg/mL)	Continuous (g = 0)
		Nerve Growth Factor - synovial fluid (pg/mL)	Continuous (g = 0.25)
		Calprotectin - synovial fluid (ng/mL)	Continuous (g = 0.25)
	Adipocytokines / Myokines - synovial fluid	Leptin - synovial fluid (pg/mL)	Continuous (g = 0.25)
		Irisin - synovial fluid (ng/mL)	Continuous (g = 0)
		Adiponectin - synovial fluid (ng/mL)	Continuous (g = -0.25)
		Omentin - synovial fluid (pg/mL)	Continuous (g = 0.25)
		Osteopontin - synovial fluid (ng/mL)	Continuous (g = 0)
		Visfatin - synovial fluid (ng/mL)	Continuous (g = 0)
		Resistin - synovial fluid (pg/mL)	Continuous (g = 0)
	Inflammation markers - plasma	C-reactive protein - plasma (mg/L)	Continuous (g = 0)
		Interleukin 6 - plasma (pg/mL)	Continuous (g = 0)
		Interleukin 8 - plasma (pg/mL)	Continuous (g = -0.25)
		Tumor Necrosis Factor alpha - plasma (pg/mL)	Continuous (g = 0)
		Nerve Growth Factor - plasma (pg/mL)	Continuous (g = 0.25)
		Calprotectin - plasma (ng/mL)	Continuous (g = -0.50)
	Adipocytokines / Myokines - plasma	Leptin - plasma (pg/mL)	Continuous (g = 0.25)
		Irisin - plasma (ng/mL)	Continuous (g = 0.75)
		Adiponectin - plasma (ng/mL)	Continuous (g = 0)
		Omentin - plasma (pg/mL)	Continuous (g = 0.50)
		Osteopontin - plasma (ng/mL)	Continuous (g = -0.25)
		Visfatin - plasma (ng/mL)	Continuous (g = -0.25)
		Resistin - plasma (pg/mL)	Continuous (g = -0.50)
KOA severity and progression	Radiography - baseline	Kellgren-Lawrence radiographic grade (1-4)	Categorical - four categories
		Osteophytes score (0-10)	Continuous
		Joint space narrowing (0-4)	Categorical - five categories
	Clinical severity - baseline	KOOS - pain (reversed, 0-100)	Continuous
		KOOS - symptoms (reversed, 0-100)	Continuous
		KOOS - functional disability (reversed, 0-100)	Continuous
	Ultrasound - baseline	Joint effusion (mm)	Continuous
		Synovial tissue thickness (mm)	Continuous
	Radiographic progression (2 years follow-up)	Kellgren-Lawrence radiographic progression (Yes / No)	Binary
		Osteophytes radiographic progression (Yes / No)	Binary
		Joint space narrowing radiographic progression (Yes / No)	Binary

Supplementary Table S3. Association between cytokines and Knee Osteoarthritis (**KOA**) outcomes in the **complete female KOA patients series**. Table cells show non-parametric correlation-like measures and 95% Confidence Intervals (95%CI) betwen cytokines and clinical, radiographic and ultrasound severity at baseline and radiographic progression. Correlation-like measures are: Spearman correlation (continuous or ordinal outcomes) and Glass rank biserial correlation (binary outcomes). Radiographic progression according to Kellgren-Lawrence staging and joint space narrowing were treated as ordinal in these analyses. Red color indicates positive correlation, blue represents negative correlation, and color intensity expresses more extreme values of the correlation coefficients. Color intensities were saturated to 0.5 and -0.5 for positive and negative correlation, respectively. **IL-6:** Interleukin 6; **IL-8:** Interleukin 8; **TNF-alpha:** Tumor Necrosis Factor alpha; **NGF:** Nerve Growth Factor; **CRP:** C-Reactive Protein; **KOA:** Knee Osteoarthritis. **KOIP:** Knee Osteoarthritis Inflammatory Phenotype.

	KOOS pain	KOOS symptoms	KOOS functional disability	Joint effusion	Synovial tissue thickness	Kellgren-Lawrence Baseline	Osteophytes Baseline	Joint space narrowing Baseline	Kellgren-Lawrence Progression	Osteophytes Progression	Joint space narrowing Progression
**CRP - synovial fluid**	0.106 [-0.065, 0.273] 0.172647	0.055 [-0.109, 0.218] 0.480493	0.121 [-0.035, 0.272] 0.1183501	0.037 [-0.123, 0.192] 0.631780	0.027 [-0.142, 0.178] 0.737452	0.079 [-0.066, 0.229] 0.308226	-0.015 [-0.157, 0.142] 0.848850	0.069 [-0.093, 0.231] 0.3730562	0.143 [-0.064, 0.343] 0.1718505	0.073 [-0.130, 0.271] 0.476703	0.161 [-0.039, 0.364] 0.115263
**CRP - plasma**	0.092 [-0.068, 0.250] 0.233347	0.008 [-0.163, 0.171] 0.917722	0.138 [-0.028, 0.297] 0.0746962	-0.033 [-0.187, 0.126] 0.675439	0.003 [-0.159, 0.157] 0.974419	0.068 [-0.076, 0.211] 0.384565	0.003 [-0.151, 0.162] 0.970962	0.019 [-0.131, 0.186] 0.8021990	0.068 [-0.129, 0.283] 0.5162111	0.023 [-0.166, 0.218] 0.824084	0.032 [-0.179, 0.252] 0.755686
**IL-6 - synovial fluid**	0.097 [-0.053, 0.252] 0.213718	0.135 [-0.010, 0.280] 0.082567	0.125 [-0.022, 0.273] 0.1064135	0.193 [0.036, 0.323] 0.012246	0.051 [-0.102, 0.195] 0.517584	0.052 [-0.093, 0.204] 0.501415	-0.106 [-0.255, 0.051] 0.172672	-0.015 [-0.163, 0.138] 0.8470682	-0.018 [-0.230, 0.196] 0.8606411	0.162 [-0.036, 0.369] 0.117072	0.200 [0.007, 0.410] 0.049446
**IL-6 - plasma**	0.005 [-0.154, 0.159] 0.943831	-0.003 [-0.149, 0.151] 0.973303	0.036 [-0.111, 0.176] 0.6409276	0.038 [-0.107, 0.200] 0.622594	-0.083 [-0.269, 0.077] 0.290938	0.006 [-0.157, 0.166] 0.942834	-0.114 [-0.267, 0.052 0.145264	-0.017 [-0.173, 0.137] 0.8284591	0.056 [-0.164, 0.240] 0.5968512	-0.228 [-0.412, -0.032] 0.026496	-0.004 [-0.215, 0.219] 0.965771
**IL-8 - synovial fluid**	0.130 [-0.036, 0.286] 0.096622	0.196 [0.034, 0.338] 0.011760	0.145 [-0.019, 0.303] 0.0636918	0.009 [-0.139, 0.161] 0.908944	0.054 [-0.094, 0.216] 0.498515	0.176 [0.038, 0.313] 0.023867	0.089 [-0.074, 0.240] 0.256650	0.139 [-0.010, 0.293] 0.0760077	0.117 [-0.083, 0.321] 0.2662429	0.055 [-0.158, 0.259] 0.593650	0.241 [0.053, 0.444] 0.018825
**IL-8 - plasma**	0.033 [-0.109, 0.192] 0.671038	0.060 [-0.072, 0.210] 0.442820	0.021 [-0.137, 0.156] 0.7876756	0.030 [-0.131, 0.191] 0.699391	0.051 [-0.123, 0.213] 0.518432	0.043 [-0.093, 0.196] 0.578107	0.009 [-0.137, 0.150] 0.906812	0.045 [-0.104, 0.203] 0.5629462	0.004 [-0.186, 0.212] 0.9733046	0.114 [-0.087, 0.307] 0.266360	0.089 [-0.110, 0.258] 0.379714
**TNF-alpha - synovial fluid**	0.061 [-0.091, 0.233] 0.452481	0.064 [-0.098, 0.221] 0.425905	0.094 [-0.076, 0.263] 0.2471124	0.009 [-0.154, 0.179] 0.907790	-0.031 [-0.192, 0.127] 0.710042	-0.003 [-0.159, 0.162] 0.969022	-0.078 [-0.223, 0.083] 0.337730	-0.084 [-0.265, 0.069] 0.2958517	0.052 [-0.163, 0.257] 0.6384822	-0.002 [-0.209, 0.205] 0.984160	0.148 [-0.078, 0.355] 0.159725
**TNF-alpha - plasma**	-0.019 [-0.180, 0.133] 0.802994	-0.004 [-0.164, 0.162] 0.962353	-0.026 [-0.183, 0.142] 0.7378056	0.080 [-0.088, 0.233] 0.302833	0.012 [-0.144, 0.163] 0.884367	-0.087 [-0.241, 0.068] 0.261137	-0.154 [-0.306, -0.008] 0.046935	-0.088 [-0.248, 0.065] 0.2563597	0.036 [-0.167, 0.233] 0.7339591	-0.012 [-0.220, 0.183] 0.910722	0.101 [-0.103, 0.307] 0.325756
**NGF- synovial fluid**	0.048 [-0.106, 0.198] 0.545127	0.064 [-0.094, 0.225] 0.414522	0.106 [-0.051, 0.257] 0.1789914	-0.013 [-0.175, 0.155] 0.868187	-0.050 [-0.216, 0.110] 0.529795	0.047 [-0.116, 0.196] 0.554938	-0.017 [-0.164, 0.140] 0.829499	0.007 [-0.144, 0.155] 0.9299662	-0.046 [-0.246, 0.160] 0.6589828	0.111 [-0.110, 0.305] 0.282672	0.054 [-0.151, 0.280] 0.602053
**NGF - plasma**	-0.078 [-0.224, 0.071] 0.313855	-0.091 [-0.246, 0.062] 0.241721	-0.099 [-0.260, 0.047] 0.2034301	0.077 [-0.079, 0.211] 0.324298	0.087 [-0.054, 0.224] 0.272677	0.023 [-0.129, 0.174] 0.768014	-0.027 [-0.178, 0.125] 0.727082	0.070 [-0.087, 0.213] 0.3653970	-0.006 [-0.206, 0.194] 0.9567602	-0.125 [-0.321, 0.077] 0.223902	0.062 [-0.127, 0.250] 0.538461
**Calprotectin - synovial fluid**	0.088 [-0.066, 0.247] 0.262794	0.142 [-0.014, 0.300] 0.070438	0.129 [-0.032, 0.283] 0.0989424	0.116 [-0.038, 0.265] 0.140374	0.135 [-0.031, 0.295] 0.090812	-0.017 [-0.163, 0.135] 0.831931	-0.016 [-0.171, 0.146] 0.835308	-0.053 [-0.214, 0.109] 0.4966793	0.124 [-0.070, 0.325] 0.2420335	0.164 [-0.038, 0.375] 0.112762	0.164 [-0.024, 0.347] 0.113266
**Calprotectin - plasma**	0.050 [-0.107, 0.211] 0.519412	-0.006 [-0.152, 0.134] 0.938829	0.067 [-0.086, 0.223] 0.3912433	0.023 [-0.135, 0.177] 0.768682	-0.047 [-0.195, 0.102] 0.549679	-0.002 [-0.181, 0.157] 0.984388	-0.013 [-0.169, 0.135] 0.864959	0.001 [-0.158, 0.155] 0.9862902	0.153 [-0.062, 0.361] 0.1439470	-0.007 [-0.185, 0.194] 0.948872	0.063 [-0.164, 0.279] 0.533744
**Leptin - synovial fluid**	0.135 [-0.013, 0.279] 0.081013	0.088 [-0.060, 0.242] 0.254356	0.225 [0.072, 0.366] 0.0033520	0.090 [-0.056, 0.226] 0.245064	0.043 [-0.116, 0.190] 0.583333	0.105 [-0.045, 0.254] 0.175552	-0.042 [-0.198, 0.096] 0.586816	0.121 [-0.031, 0.275] 0.1179575	0.172 [-0.026, 0.383] 0.1001374	0.067 [-0.120, 0.250] 0.513072	0.157 [-0.050, 0.361] 0.123332
**Leptin - plasma**	0.106 [-0.040, 0.244] 0.173251	0.029 [-0.115, 0.195] 0.705549	0.176 [0.040, 0.317] 0.0227427	-0.001 [-0.152, 0.159] 0.984908	-0.040 [-0.209, 0.127] 0.611528	0.121 [-0.022, 0.268] 0.119502	-0.019 [-0.178, 0.131] 0.804121	0.137 [-0.022, 0.288] 0.0769468	0.113 [-0.102, 0.327] 0.2809827	0.095 [-0.117, 0.311] 0.357184	0.046 [-0.197, 0.253] 0.649196
**Irisin - synovial fluid**	0.115 [-0.034, 0.268] 0.141464	0.105 [-0.056, 0.264] 0.177834	0.172 [0.019, 0.317] 0.0273393	0.090 [-0.078, 0.251] 0.251526	0.133 [-0.029, 0.286] 0.093369	0.166 [0.017, 0.306] 0.032891	0.078 [-0.088, 0.213] 0.320132	0.150 [-0.011, 0.297] 0.0552518	0.076 [-0.128, 0.307] 0.4720448	0.001 [-0.214, 0.202] 0.989492	0.111 [-0.103, 0.298] 0.277614
**Irisin - plasma**	0.020 [-0.139, 0.189] 0.799931	0.008 [-0.151, 0.163] 0.918325	0.144 [-0.018, 0.292] 0.0635070	-0.006 [-0.165, 0.155] 0.943256	-0.026 [-0.183, 0.137] 0.744818	0.134 [-0.024, 0.283] 0.085793	0.001 [-0.160, 0.151] 0.986551	0.159 [0.001, 0.301] 0.0407500	0.072 [-0.144, 0.275] 0.4913428	0.080 [-0.129, 0.273] 0.437514	-0.044 [-0.230, 0.175] 0.668025
**Adiponectin - synovial fluid**	0.027 [-0.120, 0.169] 0.725210	0.050 [-0.106, 0.211] 0.517209	-0.012 [-0.151, 0.135] 0.8735796	0.162 [0.013, 0.316] 0.035827	0.152 [0.009, 0.298] 0.052435	0.126 [-0.033, 0.287] 0.104630	0.005 [-0.145, 0.161] 0.950345	0.055 [-0.101, 0.207] 0.4777987	-0.128 [-0.330, 0.078] 0.2206489	0.080 [-0.108, 0.282] 0.436538	0.003 [-0.194, 0.202] 0.976359
**Adiponectin - plasma**	-0.051 [-0.210, 0.109] 0.510409	-0.052 [-0.206, 0.108] 0.505605	-0.079 [-0.239, 0.083] 0.3088593	-0.012 [-0.156, 0.139] 0.882101	-0.055 [-0.217, 0.105] 0.489390	0.084 [-0.060, 0.245] 0.278785	0.082 [-0.082, 0.238] 0.289479	0.072 [-0.089, 0.224] 0.3552915	-0.217 [-0.414, -0.016] 0.0382159	-0.042 [-0.261, 0.166] 0.679950	-0.160 [-0.360, 0.035] 0.114326
**Omentin - synovial fluid**	0.042 [-0.117, 0.205] 0.587873	0.095 [-0.076, 0.246] 0.225457	-0.003 [-0.167, 0.165] 0.9726616	0.126 [-0.029, 0.274] 0.107853	0.037 [-0.128, 0.195] 0.645883	0.004 [-0.164, 0.160] 0.958346	-0.043 [-0.204, 0.118] 0.586035	-0.042 [-0.194, 0.125] 0.5916123	-0.008 [-0.207, 0.202] 0.9415448	0.017 [-0.210, 0.238] 0.869613	0.056 [-0.148, 0.265] 0.584155
**Omentin - plasma**	-0.096 [-0.238, 0.055] 0.214799	-0.071 [-0.233, 0.093] 0.360105	-0.105 [-0.250, 0.051] 0.1764601	-0.037 [-0.183, 0.105] 0.634172	0.019 [-0.143, 0.190] 0.805377	0.143 [-0.011, 0.296] 0.064701	0.112 [-0.046, 0.271] 0.151278	0.078 [-0.079, 0.232] 0.3179424	-0.222 [-0.429, -0.009] 0.0338698	-0.006 [-0.232, 0.214] 0.952276	-0.187 [-0.387, 0.027] 0.065546
**Osteopontin - synovial fluid**	0.193 [0.050, 0.338] 0.012093	0.096 [-0.047, 0.243] 0.217753	0.163 [0.015, 0.304] 0.0349961	0.107 [-0.043, 0.254] 0.168054	-0.087 [-0.231, 0.058] 0.267754	-0.053 [-0.214, 0.086] 0.495447	-0.048 [-0.208, 0.115] 0.533669	-0.073 [-0.221, 0.078] 0.3493345	0.055 [-0.156, 0.263] 0.6000368	-0.100 [-0.303, 0.095] 0.331758	0.174 [-0.031, 0.377] 0.086380
**Osteopontin - plasma**	0.119 [-0.042, 0.277] 0.125627	0.046 [-0.097, 0.207] 0.555586	0.132 [-0.032, 0.277] 0.0875631	0.003 [-0.143, 0.158] 0.965672	-0.038 [-0.188, 0.110] 0.631939	0.119 [-0.029, 0.278] 0.124017	0.152 [0.006, 0.303] 0.050165	0.147 [-0.007, 0.301] 0.0569692	-0.155 [-0.351, 0.061] 0.1391145	-0.154 [-0.359, 0.051] 0.133487	0.078 [-0.117, 0.294] 0.441023
**Visfatin - synovial fluid**	0.071 [-0.085, 0.233] 0.361786	0.088 [-0.062, 0.247] 0.258380	0.092 [-0.061, 0.233] 0.2352516	0.036 [-0.119, 0.197] 0.645365	0.089 [-0.061, 0.259] 0.255956	0.151 [0.001, 0.295] 0.050630	0.092 [-0.061, 0.243] 0.239091	0.136 [-0.006, 0.270] 0.0794944	-0.019 [-0.235, 0.185] 0.8583501	0.069 [-0.130, 0.275] 0.499395	0.089 [-0.117, 0.298] 0.382014
**Visfatin - plasma**	0.025 [-0.123, 0.170] 0.749365	-0.012 [-0.157, 0.150] 0.879680	0.038 [-0.119, 0.194] 0.6260636	0.006 [-0.145, 0.159] 0.934747	0.087 [-0.072, 0.248] 0.271426	-0.003 [-0.152, 0.151] 0.968803	-0.051 [-0.208, 0.099] 0.513867	-0.041 [-0.198, 0.106] 0.5941026	-0.125 [-0.313, 0.072] 0.2326576	-0.106 [-0.310, 0.097] 0.300889	-0.166 [-0.364, 0.019] 0.103136
**Resistin - synovial fluid**	0.094 [-0.059, 0.252] 0.227636	0.049 [-0.106, 0.207] 0.527293	0.182 [0.036, 0.324] 0.0182285	-0.066 [-0.218, 0.093] 0.391851	-0.106 [-0.255, 0.051] 0.176498	-0.026 [-0.179, 0.120] 0.741997	0.003 [-0.155, 0.163] 0.972354	-0.023 [-0.169, 0.130] 0.7654880	-0.149 [-0.330, 0.053] 0.1564857	0.114 [-0.089, 0.315] 0.268060	0.051 [-0.141, 0.239] 0.617296
**Resistin - plasma**	0.033 [-0.118, 0.182] 0.670034	-0.076 [-0.222, 0.078] 0.325401	-0.008 [-0.183, 0.150] 0.9175449	0.013 [-0.145, 0.171] 0.864109	-0.077 [-0.235, 0.087] 0.325624	0.001 [-0.143, 0.161] 0.990614	-0.140 [-0.286, 0.024] 0.070643	-0.026 [-0.172, 0.122 0.7354373	-0.017 [-0.213, 0.197] 0.8706311	-0.071 [-0.276, 0.118] 0.489944	-0.016 [-0.211, 0.189] 0.873866

Supplementary Table S4. Association between cytokines and Knee Osteoarthritis (**KOA**) outcomes in the **KOIP-1 group**. Table cells show non-parametric correlation-like measures and 95% Confidence Intervals (95%CI) betwen cytokines and clinical, radiographic and ultrasound severity at baseline and radiographic progression. Correlation-like measures are: Spearman correlation (continuous or ordinal outcomes) and Glass rank biserial correlation (binary outcomes). Radiographic progression according to Kellgren-Lawrence staging and joint space narrowing were treated as ordinal in these analyses. Red color indicates positive correlation, blue represents negative correlation, and color intensity expresses more extreme values of the correlation coefficients. Color intensities were saturated to 0.5 and -0.5 for positive and negative correlation, respectively. **IL-6:** Interleukin 6; **IL-8:** Interleukin 8; **TNF-alpha:** Tumor Necrosis Factor alpha; **NGF:** Nerve Growth Factor; **CRP:** C-Reactive Protein; **KOA:** Knee Osteoarthritis. **KOIP:** Knee Osteoarthritis Inflammatory Phenotype.

	KOOS pain	KOOS symptoms	KOOS functional disability	Joint effusion	Synovial tissue thickness	Kellgren-Lawrence Baseline	Osteophytes Baseline	Joint space narrowing Baseline	Kellgren-Lawrence Progression	Osteophytes Progression	Joint space narrowing Progression
**CRP - synovial fluid**	0.057<br>[-0.212, 0.338]<br>0.6808475	0.169<br>[-0.099, 0.453]<br>0.217488	0.037<br>[-0.265, 0.324]<br>0.7880936	0.088<br>[-0.192, 0.374]<br>0.524307	0.008<br>[-0.287, 0.289]<br>0.953269	0.056<br>[-0.201, 0.316]<br>0.685440	0.245<br>[-0.037, 0.482]<br>0.071720	-0.057<br>[-0.312, 0.225]<br>0.6782933	0.322<br>[-0.024, 0.620]<br>0.0623661	-0.033<br>[-0.519, 0.439]<br>0.8768724	0.013<br>[-0.318, 0.364]<br>0.936124
**CRP - plasma**	0.018<br>[-0.254, 0.283]<br>0.8941563	0.084<br>[-0.203, 0.368]<br>0.541826	0.092<br>[-0.198, 0.374]<br>0.5044741	-0.152<br>[-0.440, 0.135]<br>0.268017	-0.096<br>[-0.350, 0.169]<br>0.491954	-0.014<br>[-0.271, 0.234]<br>0.919239	0.079<br>[-0.193, 0.325]<br>0.565797	-0.169<br>[-0.399, 0.071]<br>0.2181278	0.025<br>[-0.318, 0.360]<br>0.8860196	0.089<br>[-0.311, 0.508]<br>0.6795200	-0.110<br>[-0.449, 0.226]<br>0.508546
**IL-6 - synovial fluid**	-0.122<br>[-0.364, 0.153]<br>0.3763797	0.060<br>[-0.194, 0.328]<br>0.661141	-0.041<br>[-0.304, 0.222]<br>0.7636312	0.031<br>[-0.232, 0.289]<br>0.821858	-0.139<br>[-0.378, 0.135]<br>0.316026	0.117<br>[-0.153, 0.368]<br>0.393270	-0.165<br>[-0.429, 0.095]<br>0.228933	0.027<br>[-0.263, 0.297]<br>0.8453379	0.029<br>[-0.319, 0.373]<br>0.8686328	0.033<br>[-0.378, 0.467]<br>0.8768849	0.304<br>[0.020, 0.603]<br>0.068297
**IL-6 - plasma**	0.007<br>[-0.293, 0.277]<br>0.9621484	0.088<br>[-0.190, 0.339]<br>0.521256	0.113<br>[-0.164, 0.376]<br>0.4132549	0.078<br>[-0.215, 0.333]<br>0.573820	0.072<br>[-0.225, 0.328]<br>0.604131	0.108<br>[-0.167, 0.369]<br>0.434161	-0.083<br>[-0.340, 0.206]<br>0.547407	-0.052<br>[-0.335, 0.225]<br>0.7068630	0.055<br>[-0.292, 0.436]<br>0.7491395	-0.058<br>[-0.436, 0.333]<br>0.7863020	0.130<br>[-0.229, 0.433]<br>0.434612
**IL-8 - synovial fluid**	0.030<br>[-0.266, 0.345]<br>0.8320551	0.314<br>[0.058, 0.556]<br>0.020651	0.199<br>[-0.110, 0.466]<br>0.1488220	0.082<br>[-0.234, 0.372]<br>0.553326	0.145<br>[-0.142, 0.404]<br>0.301267	0.103<br>[-0.166, 0.345]<br>0.459411	-0.081<br>[-0.353, 0.179]<br>0.558649	0.093<br>[-0.167, 0.344]<br>0.5039524	-0.101<br>[-0.429, 0.255]<br>0.5616879	-0.006<br>[-0.442, 0.469]<br>0.9789212	0.049<br>[-0.270, 0.389]<br>0.771999
**IL-8 - plasma**	0.038<br>[-0.235, 0.309]<br>0.7821161	0.192<br>[-0.064, 0.444]<br>0.160820	0.164<br>[-0.102, 0.424]<br>0.2326517	0.058<br>[-0.215, 0.346]<br>0.675558	-0.121<br>[-0.434, 0.170]<br>0.383941	0.165<br>[-0.109, 0.434]<br>0.228820	0.028<br>[-0.242, 0.296]<br>0.840704	0.065<br>[-0.200, 0.303]<br>0.6360916	-0.112<br>[-0.459, 0.268]<br>0.5153208	0.225<br>[-0.186, 0.544]<br>0.2956847	0.164<br>[-0.167, 0.465]<br>0.326266
**TNF-alpha - synovial fluid**	0.028<br>[-0.272, 0.286]<br>0.8418532	0.124<br>[-0.164, 0.388]<br>0.370218	0.155<br>[-0.139, 0.421]<br>0.2646216	0.107<br>[-0.151, 0.356]<br>0.439213	-0.067<br>[-0.339, 0.213]<br>0.634660	0.103<br>[-0.173, 0.350]<br>0.460664	-0.053<br>[-0.340, 0.229]<br>0.702761	0.009<br>[-0.273, 0.303]<br>0.9470219	-0.236<br>[-0.560, 0.119]<br>0.1777694	0.009<br>[-0.342, 0.400]<br>0.9683949	0.154<br>[-0.180, 0.485]<br>0.362595
**TNF-alpha - plasma**	0.019<br>[-0.251, 0.305]<br>0.8908295	0.070<br>[-0.216, 0.339]<br>0.609631	0.028<br>[-0.285, 0.291]<br>0.8368486	0.208<br>[-0.072, 0.480]<br>0.126986	0.032<br>[-0.257,<br>0.309]0.819534	0.038<br>[-0.226, 0.288]<br>0.780635	-0.019<br>[-0.321, 0.269]<br>0.888887	0.002<br>[-0.293, 0.279]<br>0.9884097	0.086<br>[-0.260, 0.435]<br>0.6197631	0.178<br>[-0.300, 0.615]<br>0.4086700	0.254<br>[-0.044, 0.575]<br>0.127874
**NGF- synovial fluid**	0.028<br>[-0.274, 0.309]<br>0.8436937	0.012<br>[-0.287, 0.304]<br>0.929738	0.072<br>[-0.227, 0.369]<br>0.6101472	0.033<br>[-0.201, 0.295]<br>0.814862	0.033<br>[-0.248, 0.306]<br>0.816196	-0.001<br>[-0.303, 0.279]<br>0.994785	0.060<br>[-0.194, 0.323]<br>0.670694	-0.121<br>[-0.385, 0.170<br>0.3861629	-0.139<br>[-0.471, 0.219]<br>0.4262136	0.462<br>[0.036, 0.843]<br>0.0311171	0.040<br>[-0.304, 0.375]<br>0.812906
**NGF - plasma**	-0.211<br>[-0.445, 0.022]<br>0.1257097	-0.158<br>[-0.417, 0.145]<br>0.253666	-0.181<br>[-0.469, 0.099]<br>0.1898111	0.188<br>[-0.087, 0.414]<br>0.173928	0.129<br>[-0.136, 0.403]<br>0.357335	0.148<br>[-0.128, 0.413]<br>0.286865	-0.125<br>[-0.376, 0.150]<br>0.366560	0.141<br>[-0.135, 0.376]<br>0.3107590	-0.250<br>[-0.567, 0.084]<br>0.1492430	-0.262<br>[-0.683, 0.197]<br>0.2218059	-0.033<br>[-0.370, 0.310]<br>0.843722
**Calprotectin - synovial fluid**	0.167<br>[-0.104, 0.427]<br>0.2374352	0.222<br>[-0.061, 0.463]<br>0.113881	0.136<br>[-0.160, 0.404]<br>0.3347281	-0.033<br>[-0.298, 0.222]<br>0.814926	0.196<br>[-0.090, 0.462]<br>0.166977	-0.009<br>[-0.286, 0.268]<br>0.951929	-0.047<br>[-0.310, 0.204]<br>0.741342	-0.021<br>[-0.295, 0.242]<br>0.8831276	-0.039<br>[-0.430, 0.339]<br>0.8265385	-0.147<br>[-0.569, 0.297]<br>0.4975183	-0.002<br>[-0.365, 0.325]<br>0.991202
**Calprotectin - plasma**	-0.013<br>[-0.293, 0.283]<br>0.9252163	0.197<br>[-0.083, 0.469]<br>0.148790	0.133<br>[-0.160, 0.428]<br>0.3312908	-0.048<br>[-0.317, 0.215]<br>0.725346	-0.075<br>[-0.336, 0.213]<br>0.588316	0.070<br>[-0.177, 0.300]<br>0.609572	0.090<br>[-0.182, 0.334]<br>0.515135	0.005<br>[-0.244, 0.278]<br>0.9729602	0.181<br>[-0.204, 0.529]<br>0.2948672	0.150<br>[-0.211, 0.512]<br>0.4857171	0.130<br>[-0.190, 0.452]<br>0.434624
**Leptin - synovial fluid**	-0.076<br>[-0.386, 0.223]<br>0.5800336	-0.102<br>[-0.393, 0.231]<br>0.457513	-0.024<br>[-0.325, 0.263]<br>0.8604284	-0.159<br>[-0.426, 0.116]<br>0.245836	-0.152<br>[-0.422, 0.119]<br>0.272638	0.154<br>[-0.120, 0.419]<br>0.261320	-0.018<br>[-0.318, 0.236]<br>0.894325	0.165<br>[-0.106, 0.426]<br>0.2289894	0.017<br>[-0.313, 0.347]<br>0.9209499	0.483<br>[0.077, 0.841]<br>0.0246829	-0.043<br>[-0.396, 0.297]<br>0.794529
**Leptin - plasma**	-0.110<br>[-0.404, 0.180]<br>0.4294761	-0.077<br>[-0.374, 0.219]<br>0.579981	0.015<br>[-0.252, 0.288]<br>0.9156144	-0.158<br>[-0.413, 0.125]<br>0.253539	-0.258<br>[-0.462, -0.022]<br>0.061894	0.072<br>[-0.186, 0.326]<br>0.604795	-0.025<br>[-0.303, 0.244<br>]0.859740	0.100<br>[-0.166, 0.362]<br>0.4703454	-0.044<br>[-0.364, 0.341]<br>0.8023873	0.402<br>[-0.031, 0.762<br>]0.0625738	-0.061<br>[-0.385, 0.294]<br>0.717982
**Irisin - synovial fluid**	-0.086<br>[-0.335, 0.188]<br>0.5414083	-0.032<br>[-0.318, 0.259]<br>0.822072	-0.011<br>[-0.263, 0.236]<br>0.9387161	-0.077<br>[-0.365, 0.207]<br>0.582651	0.103<br>[-0.178, 0.387]<br>0.468699	0.268<br>[-0.007, 0.532]<br>0.052470	0.182<br>[-0.124, 0.435]<br>0.192753	0.231<br>[-0.074, 0.515]<br>0.0953657	-0.433<br>[-0.717, -0.112]<br>0.0141964	0.158<br>[-0.328, 0.578]<br>0.4652117	-0.364<br>[-0.676, -0.011]<br>0.032935
**Irisin - plasma**	-0.164<br>[-0.440, 0.115]<br>0.2365206	-0.090<br>[-0.354, 0.182]<br>0.517385	-0.098<br>[-0.375, 0.191]<br>0.4823262	-0.016<br>[-0.299, 0.275]<br>0.907568	0.055<br>[-0.194, 0.307]<br>0.697348	0.198<br>[-0.078, 0.462]<br>0.151702	0.094<br>[-0.189, 0.355]<br>0.500657	0.242<br>[-0.030, 0.515]<br>0.0777908	-0.038<br>[-0.424, 0.320]<br>0.8287400	0.356<br>[-0.205, 0.743]<br>0.1145426	-0.238<br>[-0.546, 0.112]<br>0.157131
**Adiponectin - synovial fluid**	-0.115<br>[-0.390, 0.148]<br>0.4031430	-0.007<br>[-0.295, 0.287]<br>0.961767	-0.055<br>[-0.320, 0.223]<br>0.6902647	-0.022<br>[-0.293, 0.272]<br>0.871413	0.009<br>[-0.267, 0.267]<br>0.947381	0.080<br>[-0.196, 0.349]<br>0.560681	-0.024<br>[-0.331, 0.250]<br>0.860097	0.059<br>[-0.217, 0.325]<br>0.6667851	-0.139<br>[-0.484, 0.188]<br>0.4208492	0.608<br>[0.326, 0.844]<br>0.0046935	0.070<br>[-0.268, 0.405]<br>0.673965
**Adiponectin - plasma**	-0.251<br>[-0.482, -0.004]<br>0.0641210	-0.163<br>[-0.430, 0.110]<br>0.234760	-0.197<br>[-0.439, 0.074]<br>0.1496734	-0.163<br>[-0.424, 0.111]<br>0.234479	-0.173<br>[-0.430, 0.116]<br>0.211267	0.022<br>[-0.249, 0.287]<br>0.874643	0.021<br>[-0.243, 0.280]<br>0.879067	0.140<br>[-0.132, 0.404]<br>0.3070675	-0.116<br>[-0.427, 0.231]<br>0.5011969	0.311<br>[-0.101, 0.705]<br>0.1482014	0.167<br>[-0.158, 0.498<br>0.316503
**Omentin - synovial fluid**	-0.265<br>[-0.501, -0.001]<br>0.0503973	-0.064<br>[-0.341, 0.218]<br>0.643342	-0.218<br>[-0.462, 0.037]<br>0.1100286	0.145<br>[-0.145, 0.406]<br>0.289727	-0.030<br>[-0.325, 0.242]<br>0.827211	0.034<br>[-0.257, 0.296]<br>0.805986	-0.003<br>[-0.279, 0.280]<br>0.984379	0.049<br>[-0.267, 0.317]<br>0.7237695	0.208<br>[-0.149, 0.531]<br>0.2294128	0.433<br>[0.089, 0.735]<br>0.0440146	0.435<br>[0.144, 0.723]<br>0.009205
**Omentin - plasma**	-0.233<br>[-0.484, 0.053]<br>0.0866440	-0.060<br>[-0.327, 0.224]<br>0.665615	-0.106<br>[-0.371, 0.172]<br>0.4405165	-0.072<br>[-0.337, 0.169]<br>0.601943	-0.095<br>[-0.353, 0.165]<br>0.492641	0.327<br>[0.061, 0.555]<br>0.014846	0.228<br>[-0.051, 0.455]<br>0.093683	0.365<br>[0.097, 0.598]<br>0.0061057	-0.105<br>[-0.460, 0.216]<br>0.5442162	0.506<br>[NA, NA]<br>0.0187931	-0.043<br>[-0.400, 0.310]<br>0.794529
**Osteopontin - synovial fluid**	0.097<br>[-0.183, 0.352]<br>0.4815616	0.116<br>[-0.149, 0.364]<br>0.398835	0.109<br>[-0.146, 0.350]<br>0.4303205	-0.013<br>[-0.309, 0.266]<br>0.922385	-0.092<br>[-0.348, 0.166]<br>0.506222	-0.149<br>[-0.411, 0.118]<br>0.278336	-0.120<br>[-0.379, 0.161]<br>0.381101	-0.168<br>[-0.435, 0.133]<br>0.2193841	0.042<br>[-0.314, 0.377]<br>0.8082729	0.211<br>[-0.209, 0.599]<br>0.3262690	0.244<br>[-0.092, 0.582]<br>0.143416
**Osteopontin - plasma**	0.134<br>[-0.176, 0.392]<br>0.3281943	-0.067<br>[-0.318, 0.212]<br>0.628722	0.078<br>[-0.216, 0.367]<br>0.5728687	-0.181<br>[-0.424, 0.085]<br>0.187217	-0.130<br>[-0.377, 0.130]<br>0.348827	-0.047<br>[-0.341, 0.226]<br>0.735328	-0.060<br>[-0.311, 0.174]<br>0.662141	0.000<br>[-0.272, 0.264]<br>0.9978536	-0.322<br>[-0.637, -0.004]<br>0.0623984	-0.117<br>[-0.574, 0.380]<br>0.5876686	0.047<br>[-0.289, 0.364]<br>0.779119
**Visfatin - synovial fluid**	-0.073<br>[-0.343, 0.215]<br>0.5950288	-0.009<br>[-0.292, 0.271]<br>0.949144	-0.009<br>[-0.277, 0.292]<br>0.9484762	-0.075<br>[-0.324, 0.214]<br>0.584413	0.147<br>[-0.102, 0.420]<br>0.287949	0.158<br>[-0.121, 0.431]<br>0.248905	0.036<br>[-0.219, 0.294]<br>0.794522	0.088<br>[-0.146, 0.322]<br>0.5216160	-0.149<br>[-0.489, 0.189]<br>0.3897290	-0.058<br>[-0.456, 0.378]<br>0.7862967	0.065<br>[-0.303, 0.407]<br>0.696040
**Visfatin - plasma**	0.084<br>[-0.197, 0.379]<br>0.5431717	0.017<br>[-0.283, 0.318]<br>0.900667	-0.038<br>[-0.312, 0.284]<br>0.7831750	-0.150<br>[-0.375, 0.126]<br>0.274789	0.036<br>[-0.256, 0.309]<br>0.797978	0.060<br>[-0.219, 0.334]<br>0.662137	0.018<br>[-0.221, 0.278]<br>0.894325	-0.020<br>[-0.299, 0.231]<br>0.8820434	-0.055<br>[-0.396, 0.294]<br>0.7491395	-0.117<br>[-0.523, 0.274]<br>0.5876590	-0.013<br>[-0.333, 0.309]<br>0.936129
**Resistin - synovial fluid**	-0.035<br>[-0.283, 0.225]<br>0.7977216	-0.069<br>[-0.326, 0.188]<br>0.615252	0.066<br>[-0.220, 0.316]<br>0.6297848	-0.060<br>[-0.341, 0.243]<br>0.662958	0.066<br>[-0.225, 0.328]<br>0.637103	-0.036<br>[-0.323, 0.249]<br>0.791860	0.023<br>[-0.243, 0.295]<br>0.869784	-0.066<br>[-0.353, 0.229]<br>0.6337830	-0.455<br>[-0.750, -0.142]<br>0.0084063	0.092<br>[-0.364, 0.521]<br>0.6700794	-0.038<br>[-0.368, 0.308]<br>0.817789
**Resistin - plasma**	-0.079<br>[-0.314, 0.175]<br>0.5646775	-0.124<br>[-0.379, 0.133]<br>0.366273	-0.220<br>[-0.464, 0.055]<br>0.1071002	0.012<br>[-0.273, 0.302]<br>0.930413	-0.121<br>[-0.419, 0.191]<br>0.382289	0.071<br>[-0.173, 0.317]<br>0.605001	-0.095<br>[-0.365, 0.148]<br>0.488683	-0.015<br>[-0.277, 0.233]<br>0.9136636	-0.063<br>[-0.421, 0.262]<br>0.7159553	-0.172<br>[-0.554, 0.250]<br>0.4234695	-0.013<br>[-0.337, 0.338]<br>0.936131

Supplementary Table S5. Association between cytokines and Knee Osteoarthritis (**KOA**) outcomes in the **KOIP-2 group**. Table cells show non-parametric correlation-like measures and 95% Confidence Intervals (95%CI) betwen cytokines and clinical, radiographic and ultrasound severity at baseline and radiographic progression. Correlation-like measures are: Spearman correlation (continuous or ordinal outcomes) and Glass rank biserial correlation (binary outcomes). Radiographic progression according to Kellgren-Lawrence staging and joint space narrowing were treated as ordinal in these analyses. Red color indicates positive correlation, blue represents negative correlation, and color intensity expresses more extreme values of the correlation coefficients. Color intensities were saturated to 0.5 and -0.5 for positive and negative correlation, respectively. **IL-6:** Interleukin 6; **IL-8:** Interleukin 8; **TNF-alpha:** Tumor Necrosis Factor alpha; **NGF:** Nerve Growth Factor; **CRP:** C-Reactive Protein; **KOA:** Knee Osteoarthritis. **KOIP:** Knee Osteoarthritis Inflammatory Phenotype.

	KOOS pain	KOOS symptoms	KOOS functional disability	Joint effusion	Synovial tissue thickness	Kellgren-Lawrence Baseline	Osteophytes Baseline	Joint space narrowing Baseline	Kellgren-Lawrence Progression	Osteophytes Progression	Joint space narrowing Progression
**CRP - synovial fluid**	-0.192<br>[-0.472, 0.122<br>0.181226	0.006<br>[-0.320, 0.331]<br>0.968923	-0.145<br>[-0.422, 0.148]<br>0.315037	0.022<br>[-0.286, 0.329]<br>0.878007	0.175<br>[-0.123, 0.454]<br>0.233185	0.068<br>[-0.261, 0.370]<br>0.636945	-0.065<br>[-0.337, 0.217]<br>0.651815	0.109<br>[-0.219, 0.412]<br>0.449363	0.046<br>[-0.457, 0.503]<br>0.834243	0.025<br>[-0.326, 0.405]<br>0.8967512	0.268<br>[-0.243, 0.731]<br>0.2203176
**CRP - plasma**	-0.005<br>[-0.268, 0.258]<br>0.972142	0.028<br>[-0.273, 0.308]<br>0.845896	0.005<br>[-0.273, 0.274]<br>0.973821	-0.107<br>[-0.399, 0.172]<br>0.456404	0.011<br>[-0.291, 0.300]<br>0.941143	-0.083<br>[-0.347, 0.198]<br>0.563137	0.020<br>[-0.304, 0.322]<br>0.891247	-0.020<br>[-0.347, 0.283]<br>0.886972	0.187<br>[-0.346, 0.641]<br>0.390714	-0.195<br>[-0.543, 0.150]<br>0.3015557	0.082<br>[-0.404, 0.523]<br>0.6950003
**IL-6 - synovial fluid**	0.068<br>[-0.230, 0.373]<br>0.637689	0.176<br>[-0.108, 0.467]<br>0.222709	0.139<br>[-0.188, 0.437]<br>0.335177	0.101<br>[-0.198, 0.394]<br>0.484166	0.190<br>[-0.083, 0.437]<br>0.194681	-0.080<br>[-0.367, 0.216]<br>0.581477	-0.089<br>[-0.394, 0.218]<br>0.540704	-0.102<br>[-0.390, 0.211]<br>0.480385	-0.248<br>[-0.683, 0.171]<br>0.256604	0.138<br>[-0.333, 0.538]<br>0.4752297	-0.061<br>[-0.468, 0.364]<br>0.7737450
**IL-6 - plasma**	0.033<br>[-0.288, 0.330]<br>0.820779	0.153<br>[-0.171, 0.426]<br>0.283983	0.083<br>[-0.196, 0.360]<br>0.564384	0.108<br>[-0.193, 0.377]<br>0.448654	-0.118<br>[-0.396, 0.148]<br>0.418774	-0.103<br>[-0.398, 0.198]<br>0.471828	-0.076<br>[-0.347, 0.197]<br>0.597763	-0.116<br>[-0.414, 0.182]<br>0.417632	0.098<br>[-0.335, 0.528]<br>0.651887	-0.505<br>[-0.790, -0.150]<br>0.0075425	-0.138<br>[-0.590, 0.333]<br>0.5103045
**IL-8 - synovial fluid**	0.084<br>[-0.213, 0.360]<br>0.564403	0.068<br>[-0.235, 0.348]<br>0.640122	0.096<br>[-0.170, 0.361]<br>0.512996	-0.292<br>[-0.549, -0.020]<br>0.042035	-0.114<br>[-0.374, 0.142]<br>0.445407	0.035<br>[-0.250, 0.339]<br>0.812890	0.160<br>[-0.153, 0.454]<br>0.273189	0.069<br>[-0.212, 0.330]<br>0.637051	0.347<br>[-0.150, 0.743]<br>0.114358	-0.327<br>[-0.655, 0.017]<br>0.0876540	0.306<br>[-0.126, 0.683]<br>0.1478229
**IL-8 - plasma**	0.062<br>[-0.183, 0.319]<br>0.667046	0.084<br>[-0.212, 0.353]<br>0.558917	0.001<br>[-0.286, 0.280]<br>0.993202	0.157<br>[-0.129, 0.431]<br>0.270712	0.105<br>[-0.164, 0.377]<br>0.473627	-0.109<br>[-0.356, 0.140]<br>0.447476	-0.176<br>[-0.439, 0.117]<br>0.217639	-0.145<br>[-0.403, 0.143]<br>0.310341	-0.308<br>[-0.619, 0.014]<br>0.158203	0.362<br>[-0.018, 0.710]<br>0.0554900	-0.106<br>[-0.488, 0.304]<br>0.6141905
**TNF-alpha - synovial fluid**	0.186<br>[-0.110, 0.426]<br>0.216900	0.149<br>[-0.140, 0.396]<br>0.322893	0.234<br>[-0.057, 0.504]<br>0.116843	-0.095<br>[-0.393, 0.243]<br>0.528795	0.053<br>[-0.225, 0.316]<br>0.732315	0.019<br>[-0.297, 0.312]<br>0.902523	-0.003<br>[-0.305, 0.301]<br>0.983231	-0.186<br>[-0.429, 0.108]<br>0.215943	0.153<br>[NA, NA]<br>0.508723	-0.159<br>[-0.548, 0.252]<br>0.4594471	0.062<br>[-0.419, 0.518]<br>0.7722214
**TNF-alpha - plasma**	-0.207<br>[-0.480, 0.070]<br>0.144965	-0.140<br>[-0.413, 0.151]<br>0.327216	-0.167<br>[-0.451, 0.111]<br>0.241859	-0.141<br>[-0.373, 0.118]<br>0.323836	-0.068<br>[-0.369, 0.234]<br>0.642354	-0.082<br>[-0.371, 0.214]<br>0.567212	-0.108<br>[-0.369, 0.150]<br>0.451310	-0.093<br>[-0.412, 0.229]<br>0.516493	-0.130<br>[-0.598, 0.347]<br>0.550866	-0.190<br>[-0.560, 0.181]<br>0.3134999	-0.271<br>[-0.724, 0.219]<br>0.1976563
**NGF- synovial fluid**	0.113<br>[-0.174, 0.409]<br>0.446092	0.127<br>[-0.162, 0.396]<br>0.390305	0.057<br>[-0.248, 0.328]<br>0.698342	-0.029<br>[-0.377, 0.330]<br>0.844729	-0.329<br>[-0.566, -0.033]<br>0.025810	-0.038<br>[-0.316, 0.256]<br>0.798773	-0.167<br>[-0.433, 0.136]<br>0.256092	-0.024<br>[-0.301, 0.231]<br>0.871516	-0.201<br>[-0.562, 0.185]<br>0.359345	-0.241<br>[-0.613, 0.141]<br>0.2093673	-0.083<br>[-0.467, 0.283]<br>0.7044738
**NGF - plasma**	0.036<br>[-0.238, 0.304]<br>0.800945	0.095<br>[-0.190, 0.364]<br>0.507137	-0.043<br>[-0.322, 0.275]<br>0.763257	-0.063<br>[-0.362, 0.240]<br>0.661225	0.042<br>[-0.253, 0.327]<br>0.772301	-0.244<br>[-0.499, 0.020]<br>0.084875	0.035<br>[-0.250, 0.319]<br>0.806272	-0.197<br>[-0.439, 0.085]<br>0.165288	0.102<br>[-0.305, 0.494]<br>0.640619	-0.100<br>[-0.455, 0.282]<br>0.5956756	0.124<br>[-0.295, 0.533]<br>0.5553505
**Calprotectin - synovial fluid**	0.089<br>[-0.198, 0.372]<br>0.537463	0.132<br>[-0.175, 0.446]<br>0.360214	0.083<br>[-0.227, 0.376]<br>0.568887	0.009<br>[-0.266, 0.309]<br>0.952726	0.103<br>[-0.210, 0.403]<br>0.486637	-0.206<br>[-0.472, 0.090]<br>0.150516	-0.028<br>[-0.332, 0.282]<br>0.844953	-0.141<br>[-0.431, 0.134]<br>0.329142	0.359<br>[-0.145, 0.767]<br>0.100583	0.113<br>[-0.242, 0.469]<br>0.5510842	0.321<br>[-0.118, 0.707]<br>0.1275836
**Calprotectin - plasma**	-0.054<br>[-0.359, 0.232]<br>0.704307	-0.048<br>[-0.333, 0.230]<br>0.736922	-0.134<br>[-0.376, 0.165]<br>0.348779	0.101<br>[-0.164, 0.359]<br>0.479215	-0.112<br>[-0.397, 0.182]<br>0.443180	-0.255<br>[-0.544, 0.050]<br>0.070478	-0.092<br>[-0.357, 0.212]<br>0.522538	-0.279<br>[-0.541, 0.005]<br>0.047015	-0.073<br>[-0.623, 0.511]<br>0.737926	-0.314<br>[-0.638, 0.037]<br>0.0963037	-0.341<br>[-0.790, 0.193]<br>0.1043065
**Leptin - synovial fluid**	-0.112<br>[-0.377, 0.161]<br>0.432544	-0.068<br>[-0.326, 0.214]<br>0.636232	-0.074<br>[-0.350, 0.187]<br>0.607623	-0.184<br>[-0.443, 0.084]<br>0.196678	-0.054<br>[-0.363, 0.234]<br>0.711029	0.042<br>[-0.243, 0.322]<br>0.768883	-0.125<br>[-0.379, 0.138]<br>0.383268	-0.010<br>[-0.280, 0.239]<br>0.944671	-0.333<br>[-0.727, 0.125]<br>0.126617	-0.181<br>[-0.534, 0.174]<br>0.3382929	-0.571<br>[-0.861, -0.200]<br>0.0065948
**Leptin - plasma**	-0.076<br>[-0.322, 0.227]<br>0.595469	0.024<br>[-0.270, 0.318]<br>0.868906	0.023<br>[-0.270, 0.288]<br>0.874493	-0.201<br>[-0.472, 0.078]<br>0.157256	-0.103<br>[-0.348, 0.181]<br>0.480895	-0.047<br>[-0.333, 0.246]<br>0.743424	-0.088<br>[-0.380, 0.216]<br>0.540099	-0.073<br>[-0.358, 0.199]<br>0.611502	-0.187<br>[-0.660, 0.313]<br>0.390680	-0.145<br>[-0.536, 0.220]<br>0.4421415	-0.429<br>[-0.835, 0.059]<br>0.0408989
**Irisin - synovial fluid**	0.105<br>[-0.158, 0.374]<br>0.469843	0.196<br>[-0.095, 0.454]<br>0.172175	0.142<br>[-0.146, 0.406]<br>0.324799	-0.110<br>[-0.417, 0.200]<br>0.446436	0.030<br>[-0.210, 0.267]<br>0.841649	-0.098<br>[-0.364, 0.162]<br>0.496944	-0.130<br>[-0.412, 0.167]<br>0.367196	-0.183<br>[-0.452, 0.129]<br>0.204229	-0.196<br>[-0.627, 0.248]<br>0.370455	-0.177<br>[-0.519, 0.222]<br>0.3507954	-0.224<br>[-0.651, 0.229]<br>0.2874759
**Irisin - plasma**	-0.089<br>[-0.369, 0.196]<br>0.533790	0.021<br>[-0.281, 0.290]<br>0.883898	0.067<br>[-0.261, 0.369]<br>0.638315	-0.238<br>[-0.532, 0.054]<br>0.092753	-0.091<br>[-0.369, 0.210]<br>0.534022	-0.147<br>[-0.413, 0.144]<br>0.302119	-0.186<br>[-0.458, 0.096]<br>0.190628	-0.109<br>[-0.378, 0.155]<br>0.444781	-0.137<br>[-0.544, 0.300]<br>0.531605	-0.062<br>[-0.432, 0.301]<br>0.7432374	-0.435<br>[-0.829, 0.016]<br>0.0382268
**Adiponectin - synovial fluid**	0.113<br>[-0.131, 0.374]<br>0.429112	0.210<br>[-0.095, 0.450]<br>0.138468	0.132<br>[-0.128, 0.390]<br>0.355233	0.231<br>[-0.082, 0.508]<br>0.102952	0.314<br>[0.018, 0.573]<br>0.028268	0.011<br>[-0.258, 0.289]<br>0.941083	0.086<br>[-0.216, 0.369]<br>0.546396	-0.046<br>[-0.321, 0.247]<br>0.749713	-0.003<br>[-0.494, 0.456]<br>0.988393	-0.305<br>[-0.629, 0.028]<br>0.1068209	0.188<br>[-0.265, 0.600]<br>0.3701570
**Adiponectin - plasma**	0.111<br>[-0.156, 0.378]<br>0.439477	0.000<br>[-0.315, 0.294]<br>0.998736	0.079<br>[-0.185, 0.344]<br>0.582468	0.149<br>[-0.126, 0.433]<br>0.296014	-0.026<br>[-0.319, 0.291]<br>0.859540	-0.055<br>[-0.310, 0.240]<br>0.700631	0.186<br>[-0.096, 0.453]<br>0.190198	-0.099<br>[-0.384, 0.210]<br>0.488163	0.333<br>[-0.014, 0.645]<br>0.126604	-0.093<br>[-0.506, 0.307]<br>0.6231517	0.424<br>[0.124, 0.701]<br>0.0437430
**Omentin - synovial fluid**	0.277<br>[-0.013, 0.550]<br>0.048755	0.265<br>[-0.033, 0.515]<br>0.059832	0.228<br>[-0.093, 0.527]<br>0.107389	0.152<br>[-0.166, 0.461]<br>0.287251	0.106<br>[-0.177, 0.414]<br>0.470166	-0.095<br>[-0.383, 0.178]<br>0.506556	-0.022<br>[-0.325, 0.268]<br>0.880286	-0.114<br>[-0.388, 0.195]<br>0.426190	0.162<br>[-0.371, 0.682]<br>0.458123	-0.357<br>[-0.687, 0.024]<br>0.0587817	0.147<br>[-0.340, 0.583]<br>0.4838399
**Omentin - plasma**	0.171<br>[-0.113, 0.462]<br>0.230413	0.150<br>[-0.141, 0.416]<br>0.293784	0.136<br>[-0.145, 0.436]<br>0.340476	0.096<br>[-0.168, 0.363]<br>0.501140	0.083<br>[-0.228, 0.395]<br>0.570988	-0.086<br>[-0.348, 0.198]<br>0.546971	0.196<br>[-0.089, 0.468]<br>0.168473	-0.134<br>[-0.412, 0.163]<br>0.348884	0.133<br>[-0.260, 0.528]<br>0.541179	-0.112<br>[-0.535, 0.319]<br>0.5537400	0.103<br>[-0.283, 0.482]<br>0.6240523
**Osteopontin - synovial fluid**	0.183<br>[-0.102, 0.471]<br>0.198656	0.064<br>[-0.199, 0.346]<br>0.654993	0.228<br>[-0.041, 0.469]<br>0.107057	0.014<br>[-0.256, 0.302]<br>0.923357	-0.236<br>[-0.476, 0.041]<br>0.103114	-0.210<br>[-0.475, 0.076]<br>0.138604	-0.014<br>[-0.305, 0.293]<br>0.922460	-0.104<br>[-0.417, 0.198]<br>0.465656	0.121<br>[-0.344, 0.613]<br>0.580254	-0.371<br>[-0.691, -0.021]<br>0.0492861	0.029<br>[-0.403, 0.509]<br>0.8885989
**Osteopontin - plasma**	0.098<br>[-0.207, 0.379]<br>0.494813	0.167<br>[-0.110, 0.416]<br>0.240444	0.169<br>[-0.097, 0.410]<br>0.237094	0.171<br>[-0.083, 0.439]<br>0.229920	-0.085<br>[-0.382, 0.228<br>]0.561076	0.178<br>[-0.114, 0.451]<br>0.210418	0.249<br>[-0.048, 0.501]<br>0.078544	0.197<br>[-0.091, 0.438]<br>0.166936	-0.137<br>[-0.518, 0.289]<br>0.531605	-0.395<br>[-0.738, -0.019]<br>0.0364919	-0.224<br>[-0.590, 0.143]<br>0.2872323
**Visfatin - synovial fluid**	0.089<br>[-0.179, 0.359]<br>0.534199	0.135<br>[-0.139, 0.395]<br>0.345943	0.124<br>[-0.152, 0.386]<br>0.385418	0.197<br>[-0.104, 0.513]<br>0.166031	0.137<br>[-0.143, 0.424]<br>0.346373	0.126<br>[-0.151, 0.392]<br>0.376532	0.172<br>[-0.129, 0.445]<br>0.227533	0.196<br>[-0.077, 0.454]<br>0.167084	0.162<br>[-0.245, 0.537]<br>0.458107	0.021<br>[-0.338, 0.374]<br>0.9097187	-0.035<br>[-0.510, 0.460]<br>0.8665522
**Visfatin - plasma**	-0.252<br>[-0.511, -0.004]<br>0.074890	-0.253<br>[-0.492, 0.017]<br>0.073414	-0.156<br>[-0.425, 0.121]<br>0.275054	0.154<br>[-0.140, 0.405]<br>0.280186	0.208<br>[-0.085, 0.464]<br>0.152450	0.078<br>[-0.232, 0.347]<br>0.588722	0.007<br>[-0.302, 0.289]<br>0.961830	-0.065<br>[-0.329, 0.212]<br>0.651418	-0.537<br>[-0.829, -0.177]<br>0.013948	-0.143<br>[-0.561, 0.302]<br>0.4496918	-0.582<br>[-0.873, -0.211]<br>0.0055618
**Resistin - synovial fluid**	0.152<br>[-0.149, 0.425]<br>0.285498	-0.037<br>[-0.313, 0.210]<br>0.795390	0.127<br>[-0.143, 0.403]<br>0.375626	-0.077<br>[-0.371, 0.222]<br>0.590927	-0.249<br>[-0.528, 0.057]<br>0.084067	-0.175<br>[-0.438, 0.118]<br>0.218957	-0.034<br>[-0.299, 0.236]<br>0.811473	-0.095<br>[-0.383, 0.174]<br>0.506133	0.162<br>[-0.292, 0.579]<br>0.457790	0.169<br>[-0.197, 0.522]<br>0.3706916	0.241<br>[-0.225, 0.660]<br>0.2505229
**Resistin - plasma**	-0.064<br>[-0.320, 0.222]<br>0.653606	-0.181<br>[-0.461, 0.145]<br>0.202636	-0.100<br>[-0.355, 0.171]<br>0.486370	0.113<br>[-0.160, 0.350]<br>0.429731	0.099<br>[-0.174, 0.363]<br>0.496551	-0.112<br>[-0.392, 0.191]<br>0.435977	-0.199<br>[-0.482, 0.068]<br>0.161904	-0.194<br>[-0.455, 0.087]<br>0.172896	-0.295<br>[-0.715, 0.123]<br>0.176056	-0.236<br>[-0.557, 0.115]<br>0.2122777	-0.185<br>[-0.596, 0.342]<br>0.3776646

Supplementary Table S6. Association between cytokines and Knee Osteoarthritis (**KOA**) outcomes in the **KOIP-3 group**. Table cells show non-parametric correlation-like measures and 95% Confidence Intervals (95%CI) betwen cytokines and clinical, radiographic and ultrasound severity at baseline and radiographic progression. Correlation-like measures are: Spearman correlation (continuous or ordinal outcomes) and Glass rank biserial correlation (binary outcomes). Radiographic progression according to Kellgren-Lawrence staging and joint space narrowing were treated as ordinal in these analyses. Red color indicates positive correlation, blue represents negative correlation, and color intensity expresses more extreme values of the correlation coefficients. Color intensities were saturated to 0.5 and -0.5 for positive and negative correlation, respectively. **IL-6:** Interleukin 6; **IL-8:** Interleukin 8; **TNF-alpha:** Tumor Necrosis Factor alpha; **NGF:** Nerve Growth Factor; **CRP:** C-Reactive Protein; **KOA:** Knee Osteoarthritis. **KOIP:** Knee Osteoarthritis Inflammatory Phenotype.

	KOOS pain	KOOS symptoms	KOOS functional disability	Joint effusion	Synovial tissue thickness	Kellgren-Lawrence Baseline	Osteophytes Baseline	Joint space narrowing Baseline	Kellgren-Lawrence Progression	Osteophytes Progression	Joint space narrowing Progression
**CRP - synovial fluid**	0.230<br>[-0.194, 0.591]<br>0.248541	0.012<br>[-0.402, 0.396]<br>0.952875	0.117<br>[-0.285, 0.504]<br>0.56014662	0.213<br>[-0.194, 0.534]<br>0.285651	0.078<br>[-0.345, 0.456]<br>0.698219	0.197<br>[-0.194, 0.531]<br>0.324519	0.006<br>[-0.410, 0.480]<br>0.9743342	-0.015<br>[-0.415, 0.392]<br>0.940895	-0.582<br>[NA, NA]<br>0.035734	0.182<br>[-0.400, 0.722]<br>0.4813214	0.061<br>[-0.618, 0.706]<br>0.8229019
**CRP - plasma**	0.261<br>[-0.229, 0.596]<br>0.188318	0.157<br>[-0.266, 0.552]<br>0.435394	0.196<br>[-0.271, 0.595]<br>0.32842050	0.321<br>[-0.071, 0.599]<br>0.102142	0.354<br>[-0.002, 0.618]<br>0.069830	0.229<br>[-0.145, 0.565]<br>0.249803	0.095<br>[-0.320, 0.511]<br>0.6378534	-0.058<br>[-0.487, 0.365]<br>0.773816	-0.429<br>[-0.919, 0.131]<br>0.122289	-0.109<br>[-0.636, 0.461]<br>0.6726548	0.204<br>[NA, NA]<br>0.4556390
**IL-6 - synovial fluid**	0.070<br>[-0.374, 0.513]<br>0.730124	0.099<br>[-0.294, 0.473]<br>0.621531	-0.138<br>[-0.529, 0.286]<br>0.49322508	0.541<br>[0.255, 0.731]<br>0.003598	0.125<br>[-0.318, 0.522]<br>0.533631	0.250<br>[-0.162, 0.562]<br>0.208900	0.002<br>[-0.309, 0.375]<br>0.9938882	0.102<br>[-0.294, 0.483]<br>0.612776	-0.231<br>[-0.750, 0.333]<br>0.405381	0.273<br>[-0.245, 0.769]<br>0.2908457	0.184<br>[-0.408, 0.778]<br>0.5019387
**IL-6 - plasma**	0.098<br>[-0.367, 0.544]<br>0.634323	0.108<br>[-0.330, 0.487]<br>0.599393	-0.213<br>[-0.565, 0.202]<br>0.29583152	0.067<br>[-0.345, 0.424]<br>0.746133	-0.237<br>[-0.615, 0.168]<br>0.243464	0.070<br>[-0.300, 0.393]<br>0.735083	-0.069<br>[-0.483, 0.376]<br>0.7374701	0.001<br>[-0.400, 0.397]<br>0.995840	-0.024<br>[-0.514, 0.595]<br>0.932647	-0.273<br>[-0.758, 0.309]<br>0.3050589	0.381<br>[NA, NA]<br>0.1869493
**IL-8 - synovial fluid**	0.222<br>[-0.229, 0.596]<br>0.265699	0.329<br>[-0.110, 0.657]<br>0.093377	0.062<br>[-0.429, 0.464]<br>0.75747769	0.373<br>[0.039, 0.637]<br>0.055120	0.273<br>[-0.143, 0.613]<br>0.169035	0.215<br>[-0.203, 0.569]<br>0.281185	0.286<br>[-0.049, 0.584]<br>0.1483444	0.146<br>[-0.254, 0.518]<br>0.468515	0.363<br>[-0.187, 0.820]<br>0.191040	0.109<br>[-0.418, 0.655]<br>0.6726548	0.551<br>[0.067, 0.918]<br>0.0439752
**IL-8 - plasma**	0.104<br>[-0.329, 0.494]<br>0.604240	0.045<br>[-0.314, 0.401]<br>0.821852	-0.049<br>[-0.415, 0.353]<br>0.80987602	-0.287<br>[-0.584, 0.068]<br>0.146647	-0.144<br>[-0.618, 0.308]<br>0.474399	-0.158<br>[-0.520, 0.229]<br>0.432082	0.200<br>[-0.192, 0.584]<br>0.3173169	0.084<br>[-0.285, 0.462]<br>0.675329	0.538<br>[0.000, 0.938]<br>0.052204	0.055<br>[-0.469, 0.578]<br>0.8326894	0.531<br>[-0.022, 1.000]<br>0.0524134
**TNF-alpha - synovial fluid**	-0.030<br>[-0.407, 0.374]<br>0.881729	-0.018<br>[-0.415, 0.350]<br>0.929964	-0.163<br>[-0.566, 0.286]<br>0.41540770	-0.051<br>[-0.459, 0.376]<br>0.800525	-0.093<br>[-0.481, 0.368]<br>0.646096	-0.251<br>[-0.591, 0.093]<br>0.206947	0.020<br>[-0.329, 0.346]<br>0.9218854	-0.295<br>[-0.619, 0.057]<br>0.135553	0.275<br>[-0.238, 0.750]<br>0.321912	0.291<br>[-0.204, 0.763]<br>0.2598752	0.531<br>[NA, NA]<br>0.0524134
**TNF-alpha - plasma**	-0.123<br>[-0.491, 0.267]<br>0.549624	0.095<br>[-0.291, 0.490]<br>0.643169	-0.224<br>[-0.580, 0.213]<br>0.27036970	0.124<br>[-0.301, 0.492<br>0.545621	-0.019<br>[-0.364, 0.335]<br>0.925976	-0.264<br>[-0.595, 0.105]<br>0.192837	-0.408<br>[-0.694, -0.057]<br>0.0386679	-0.274<br>[-0.660, 0.121]<br>0.175237	-0.190<br>[-0.762, 0.428]<br>0.498962	-0.434<br>[-0.858, 0.125]<br>0.1023776	0.071<br>[-0.524, 0.667]<br>0.8045709
**NGF- synovial fluid**	-0.148<br>[-0.520, 0.257]<br>0.461117	0.010<br>[-0.420, 0.461]<br>0.960321	0.172<br>[-0.272, 0.576]<br>0.39219769	-0.014<br>[-0.434, 0.359]<br>0.946414	0.033<br>[-0.317, 0.370]<br>0.869517	0.144<br>[-0.295, 0.539]<br>0.475105	0.194<br>[-0.175, 0.539]<br>0.3310559	0.137<br>[-0.255, 0.486]<br>0.496702	-0.088<br>[-0.625, 0.475]<br>0.747662	-0.227<br>[-0.718, 0.275]<br>0.3726152	-0.204<br>[-0.704, 0.275]<br>0.4498428
**NGF - plasma**	-0.339<br>[-0.631, 0.037]<br>0.084025	-0.478<br>[-0.784, -0.062]<br>0.011621	-0.621<br>[-0.800, -0.352]<br>0.00054013	0.284<br>[-0.139, 0.676]<br>0.150438	0.194<br>[-0.148, 0.513]<br>0.332852	0.111<br>[-0.273, 0.504]<br>0.582444	0.100<br>[-0.311, 0.463]<br>0.6207338	0.141<br>[-0.256, 0.463]<br>0.482475	-0.132<br>[-0.677, 0.437]<br>0.631365	-0.145<br>[-0.654, 0.364]<br>0.5701812	-0.276<br>[-0.731, 0.231]<br>0.3100560
**Calprotectin - synovial fluid**	-0.070<br>[-0.502, 0.371]<br>0.727834	0.104<br>[-0.327, 0.509]<br>0.604377	0.047<br>[-0.419, 0.479]<br>0.81577987	0.255<br>[-0.119, 0.560]<br>0.199743	0.150<br>[-0.271, 0.518]<br>0.456147	0.069<br>[-0.331, 0.435]<br>0.733513	0.008<br>[-0.381, 0.376]<br>0.9682261	-0.159<br>[-0.542, 0.261]<br>0.427295	0.011<br>[-0.531, 0.548]<br>0.968395	0.091<br>[-0.491, 0.618]<br>0.7247712	0.245<br>[NA, NA]<br>0.3706500
**Calprotectin - plasma**	0.014<br>[-0.370, 0.406]<br>0.944335	-0.109<br>[-0.497, 0.267]<br>0.589533	-0.143<br>[-0.533, 0.294]<br>0.47691063	-0.032<br>[-0.435, 0.386]<br>0.875019	0.096<br>[-0.309, 0.488]<br>0.634117	0.278<br>[-0.207, 0.665]<br>0.159925	0.450<br>[0.096, 0.738]<br>0.0184255	0.364<br>[-0.001, 0.711]<br>0.061790	0.143<br>[-0.455, 0.750]<br>0.606498	0.473<br>[0.000, 0.852]<br>0.0671207	0.265<br>[-0.293, 0.782]<br>0.3321157
**Leptin - synovial fluid**	-0.013<br>[-0.409, 0.409]<br>0.946752	0.008<br>[-0.374, 0.397]<br>0.969782	0.114<br>[-0.312, 0.527]<br>0.57047679	0.057<br>[-0.280, 0.400]<br>0.778359	-0.034<br>[-0.434, 0.354]<br>0.865467	0.070<br>[-0.334, 0.449]<br>0.727033	-0.437<br>[-0.714, -0.036]<br>0.0226561	-0.071<br>[-0.487, 0.322]<br>0.723484	-0.209<br>[-0.714, 0.319]<br>0.451566	-0.236<br>[-0.740, 0.259]<br>0.3599647	0.082<br>[NA, NA]<br>0.7653835
**Leptin - plasma**	-0.007<br>[-0.387, 0.379]<br>0.973362	0.068<br>[-0.303, 0.413]<br>0.735142	0.062<br>[-0.358, 0.451]<br>0.75866749	-0.049<br>[-0.452, 0.367]<br>0.808731	0.126<br>[-0.258, 0.506]<br>0.532626	0.122<br>[-0.287, 0.565]<br>0.543971	-0.448<br>[-0.694, -0.081]<br>0.0191592	-0.105<br>[-0.497, 0.302<br>0.602765	-0.407<br>[NA, NA]<br>0.142650	-0.491<br>[-0.904, 0.000]<br>0.0572650	-0.143<br>[-0.788, 0.509]<br>0.6015081
**Irisin - synovial fluid**	-0.013<br>[-0.410, 0.381]<br>0.947960	0.079<br>[-0.322, 0.472]<br>0.694270	-0.097<br>[-0.450, 0.294]<br>0.63091845	0.224<br>[-0.170, 0.550]<br>0.262268	0.116<br>[-0.341, 0.496]<br>0.563177	0.144<br>[-0.325, 0.583]<br>0.473691	-0.353<br>[-0.700, 0.069]<br>0.0713304	-0.013<br>[-0.401, 0.409]<br>0.948429	-0.319<br>[NA, NA]<br>0.250547	-0.582<br>[-0.927, -0.150]<br>0.0242357	0.122<br>[-0.425, 0.615]<br>0.6544215
**Irisin - plasma**	-0.112<br>[-0.491, 0.331]<br>0.579430	0.067<br>[-0.341, 0.463]<br>0.740877	0.049<br>[-0.337, 0.432]<br>0.80990452	0.124<br>[-0.277, 0.502]<br>0.537725	-0.026<br>[-0.445, 0.425]<br>0.898903	0.206<br>[-0.212, 0.588]<br>0.302407	-0.306<br>[-0.651, 0.076]<br>0.1204924	0.062<br>[-0.352, 0.460]<br>0.756950	-0.429<br>[-0.868, 0.151]<br>0.122289	-0.436<br>[-0.855, 0.133]<br>0.0910224	-0.388<br>[-0.857, 0.206]<br>0.1563399
**Adiponectin - synovial fluid**	0.024<br>[-0.363, 0.358]<br>0.906950	0.055<br>[-0.290, 0.424]<br>0.783724	-0.060<br>[-0.483, 0.346]<br>0.76791642	0.265<br>[-0.145, 0.608]<br>0.181916	-0.129<br>[-0.492, 0.302]<br>0.522620	0.234<br>[-0.193, 0.641]<br>0.239710	-0.078<br>[-0.480, 0.297]<br>0.6978681	0.267<br>[-0.183, 0.598]<br>0.178132	-0.407<br>[-0.875, 0.140]<br>0.142650	0.182<br>[-0.353, 0.666]<br>0.4813214	-0.020<br>[NA, NA]<br>0.9405292
**Adiponectin - plasma**	-0.033<br>[-0.417, 0.362]<br>0.869757	0.112<br>[-0.243, 0.437]<br>0.579028	0.156<br>[-0.297, 0.559]<br>0.43782418	-0.096<br>[-0.481, 0.278]<br>0.635255	-0.355<br>[-0.652, 0.021]<br>0.069233	0.280<br>[-0.201, 0.699]<br>0.157229	-0.161<br>[-0.527, 0.256]<br>0.4217065	0.240<br>[-0.218, 0.604]<br>0.227355	-0.495<br>[-1.000, 0.142]<br>0.074592	-0.127<br>[-0.596, 0.407]<br>0.6220655	-0.551<br>[-1.000, 0.050]<br>0.0439752
**Omentin - synovial fluid**	0.165<br>[-0.198, 0.486]<br>0.410943	0.266<br>[-0.115, 0.553]<br>0.179903	0.237<br>[-0.172, 0.579]<br>0.23390323	0.093<br>[-0.305, 0.462]<br>0.643962	-0.226<br>[-0.574, 0.206]<br>0.256878	0.160<br>[-0.217, 0.537]<br>0.426532	-0.219<br>[-0.578, 0.198]<br>0.2721665	0.169<br>[-0.176, 0.520]<br>0.398809	-0.209<br>[NA, NA]<br>0.451566	0.182<br>[-0.363, 0.694]<br>0.4813214	-0.020<br>[-0.667, 0.646]<br>0.9405292
**Omentin - plasma**	-0.190<br>[-0.550, 0.214]<br>0.341452	0.099<br>[-0.268, 0.449]<br>0.622610	0.066<br>[-0.337, 0.474]<br>0.74256026	-0.041<br>[-0.422, 0.342]<br>0.840559	-0.009<br>[-0.396, 0.392]<br>0.962609	0.235<br>[-0.178, 0.581]<br>0.238994	-0.283<br>[-0.638, 0.137]<br>0.1523455	0.052<br>[-0.364, 0.439]<br>0.795726	-0.604<br>[-1.000, 0.020]<br>0.029318	-0.291<br>[-0.799, 0.241]<br>0.2598752	-0.408<br>[NA, NA]<br>0.1356745
**Osteopontin - synovial fluid**	-0.078<br>[-0.478, 0.342]<br>0.699415	-0.140<br>[-0.532, 0.332]<br>0.487456	-0.129<br>[-0.524, 0.308]<br>0.52268434	0.184<br>[-0.220, 0.564]<br>0.357763	0.168<br>[-0.264, 0.545]<br>0.401329	0.143<br>[-0.342, 0.543]<br>0.476928	0.035<br>[-0.387, 0.431]<br>0.8625164	-0.045<br>[-0.485, 0.376]<br>0.822667	0.033<br>[NA, NA]<br>0.905383	-0.236<br>[-0.714, 0.327]<br>0.3599647	0.714<br>[0.345, 0.978]<br>0.0090234
**Osteopontin - plasma**	0.253<br>[-0.195, 0.619]<br>0.202447	0.079<br>[-0.344, 0.522]<br>0.694270	0.205<br>[-0.166, 0.516]<br>0.30587029	0.012<br>[-0.368, 0.412]<br>0.954181	0.119<br>[-0.291, 0.468]<br>0.553929	0.484<br>[0.088, 0.759]<br>0.010467	0.294<br>[-0.111, 0.609]<br>0.1366053	0.450<br>[0.071, 0.745]<br>0.018620	-0.363<br>[-0.824, 0.187]<br>0.191040	-0.091<br>[-0.625, 0.442]<br>0.7247712	0.306<br>[-0.300, 0.827]<br>0.2631100
**Visfatin - synovial fluid**	-0.112<br>[-0.467, 0.263]<br>0.577334	-0.204<br>[-0.556, 0.251]<br>0.308513	-0.156<br>[-0.552, 0.312]<br>0.43691518	0.010<br>[-0.353, 0.390]<br>0.961409	-0.108<br>[-0.433, 0.267]<br>0.592386	0.236<br>[-0.127, 0.568]<br>0.236855	0.144<br>[-0.245, 0.540]<br>0.4748702	0.233<br>[-0.132, 0.585]<br>0.242943	-0.231<br>[NA, NA]<br>0.405381	0.309<br>[-0.218, 0.745]<br>0.2312660	0.204<br>[-0.375, 0.789]<br>0.4556390
**Visfatin - plasma**	0.110<br>[-0.281, 0.463]<br>0.584682	0.107<br>[-0.275, 0.470]<br>0.594816	0.207<br>[-0.164, 0.511]<br>0.30000485	-0.057<br>[-0.481, 0.396]<br>0.778359	-0.043<br>[-0.460, 0.357]<br>0.831075	-0.024<br>[-0.395, 0.335]<br>0.905655	0.043<br>[-0.374, 0.443]<br>0.8312879	0.117<br>[-0.251, 0.489]<br>0.562360	0.165<br>[-0.438, 0.736]<br>0.552297	0.218<br>[-0.350, 0.709]<br>0.3981027	-0.082<br>[NA, NA]<br>0.7653835
**Resistin - synovial fluid**	-0.130<br>[-0.482, 0.261]<br>0.517024	0.132<br>[-0.294, 0.513]<br>0.511300	0.241<br>[-0.172, 0.593]<br>0.22615963	-0.146<br>[-0.549, 0.295]<br>0.466435	-0.018<br>[-0.392, 0.429]<br>0.930092	-0.085<br>[-0.411, 0.246]<br>0.672054	0.070<br>[-0.425, 0.554]<br>0.7276493	-0.201<br>[-0.559, 0.246]<br>0.313698	0.033<br>[NA, NA]<br>0.905347	-0.209<br>[NA, NA]<br>0.4179007	0.184<br>[-0.367, 0.725]<br>0.5017999
**Resistin - plasma**	0.068<br>[-0.357, 0.478]<br>0.734709	0.188<br>[-0.203, 0.549]<br>0.347853	0.206<br>[-0.167, 0.537]<br>0.30292871	-0.265<br>[-0.604, 0.135]<br>0.180873	-0.347<br>[-0.649, 0.051]<br>0.076426	-0.128<br>[-0.502, 0.297]<br>0.525673	0.269<br>[-0.126, 0.596]<br>0.1749467	-0.033<br>[-0.399, 0.343]<br>0.872137	0.319<br>[NA, NA]<br>0.250547	0.182<br>[-0.333, 0.709]<br>0.4813214	-0.082<br>[-0.684, 0.537]<br>0.7653835

Supplementary Table S7. Association between cytokines and Knee Osteoarthritis (**KOA**) outcomes in the **KOIP-4 group**. Table cells show non-parametric correlation-like measures and 95% Confidence Intervals (95%CI) betwen cytokines and clinical, radiographic and ultrasound severity at baseline and radiographic progression. Correlation-like measures are: Spearman correlation (continuous or ordinal outcomes) and Glass rank biserial correlation (binary outcomes). Radiographic progression according to Kellgren-Lawrence staging and joint space narrowing were treated as ordinal in these analyses. Red color indicates positive correlation, blue represents negative correlation, and color intensity expresses more extreme values of the correlation coefficients. Color intensities were saturated to 0.5 and -0.5 for positive and negative correlation, respectively. **IL-6:** Interleukin 6; **IL-8:** Interleukin 8; **TNF-alpha:** Tumor Necrosis Factor alpha; **NGF:** Nerve Growth Factor; **CRP:** C-Reactive Protein; **KOA:** Knee Osteoarthritis. **KOIP:** Knee Osteoarthritis Inflammatory Phenotype.

	**KOOS pain**	**KOOS symptoms**	**KOOS functional disability**	**Joint effusion**	**Synovial tissue thickness**	**Kellgren-Lawrence Baseline**	**Osteophytes Baseline**	**Joint space narrowing Baseline**	**Kellgren-Lawrence Progression**	**Osteophytes Progression**	**Joint space narrowing Progression**
**CRP - synovial fluid**	-0.051<br>[-0.370, 0.265]<br>0.773244	-0.032<br>[-0.377, 0.313]<br>0.8549996	0.001<br>[-0.328, 0.301]<br>0.994261	-0.590<br>[-0.771, -0.327]<br>0.00019264	-0.339<br>[-0.645, 0.018]<br>0.0539382	-0.272<br>[-0.593, 0.087]<br>0.1145569	-0.354<br>[-0.649, 0.009]<br>0.03995225	-0.180<br>[-0.496, 0.179]<br>0.2996134	-0.006<br>[-0.523, 0.523]<br>0.9812838	-0.024<br>[-0.469, 0.412]<br>0.913073	0.026<br>[-0.458, 0.565]<br>0.908632
**CRP - plasma**	-0.178<br>[-0.455, 0.152]<br>0.306910	-0.226<br>[-0.518, 0.133]<br>0.1918653	-0.074<br>[-0.393, 0.263]<br>0.673283	-0.512<br>[-0.738, -0.209]<br>0.00167225	-0.327<br>[-0.663, 0.030]<br>0.0630728	-0.140<br>[-0.464, 0.215]<br>0.4217320	-0.271<br>[-0.554, 0.063]<br>0.12169892	-0.072<br>[-0.384, 0.256]<br>0.6809147	-0.011<br>[-0.528, 0.456]<br>0.9625946	0.114<br>[-0.288, 0.567]<br>0.600471	-0.284<br>[-0.731, 0.210]<br>0.215403
**IL-6 - synovial fluid**	0.165<br>[-0.126, 0.427]<br>0.343170	0.299<br>[-0.021, 0.591]<br>0.0807111	0.275<br>[-0.030, 0.559]<br>0.109906	0.218<br>[-0.134, 0.511]<br>0.20763705	0.037<br>[-0.342, 0.376]<br>0.8359898	-0.272<br>[-0.578, 0.087]<br>0.1139323	-0.157<br>[-0.503, 0.172]<br>0.37428950	-0.409<br>[-0.658, -0.124]<br>0.0147857	0.000<br>[NA, NA]<br>1.0000000	0.314<br>[-0.121, 0.692]<br>0.149800	0.232<br>[-0.278, 0.700]<br>0.312769
**IL-6 - plasma**	-0.405<br>[-0.695, -0.061]<br>0.015816	-0.475<br>[-0.734, -0.142]<br>0.0039018	-0.305<br>[-0.617, 0.032]<br>0.074699	-0.368<br>[-0.638, -0.039]<br>0.02979765	-0.405<br>[-0.663, -0.062]<br>0.0192431	-0.353<br>[-0.652, -0.006]<br>0.0377034	-0.376<br>[-0.668, 0.015]<br>0.02824512	-0.172<br>[-0.530, 0.203]<br>0.3236348	-0.216<br>[-0.659, 0.250]<br>0.3728437	-0.186<br>[-0.635, 0.327]<br>0.394683	-0.442<br>[-0.768, -0.010]<br>0.053939
**IL-8 - synovial fluid**	-0.018<br>[-0.357, 0.318]<br>0.920388	0.204<br>[-0.133, 0.523]<br>0.2402526	-0.025<br>[-0.362, 0.317]<br>0.888666	-0.163<br>[-0.488, 0.180]<br>0.35017725	-0.094<br>[-0.418, 0.278]<br>0.6013952	0.243<br>[-0.068, 0.496]<br>0.1600033	0.157<br>[-0.193, 0.458]<br>0.37622660	0.089<br>[-0.270, 0.452]<br>0.6100978	-0.153<br>[-0.560, 0.268]<br>0.5260091	0.233<br>[-0.202, 0.625]<br>0.284296	0.242<br>[-0.194, 0.667]<br>0.290449
**IL-8 - plasma**	-0.092<br>[-0.416, 0.239]<br>0.598623	-0.112<br>[-0.426, 0.218]<br>0.5220963	-0.025<br>[-0.390, 0.317]<br>0.888143	0.084<br>[-0.282, 0.436]<br>0.63166748	0.482<br>[0.162, 0.687]<br>0.0045403	0.125<br>[-0.192, 0.475]<br>0.4726442	0.125<br>[-0.189, 0.458]<br>0.48140979	0.266<br>[-0.071, 0.559]<br>0.1220068	0.159<br>[-0.321, 0.602]<br>0.5114100	-0.500<br>[-0.829, -0.091]<br>0.021930	0.000<br>[-0.478, 0.442]<br>1.000000
**TNF-alpha - synovial fluid**	-0.012<br>[-0.408, 0.374]<br>0.952326	-0.054<br>[-0.442, 0.369]<br>0.7865293	-0.190<br>[-0.558, 0.210]<br>0.332823	-0.156<br>[-0.483, 0.245]<br>0.42877646	0.139<br>[-0.262, 0.542]<br>0.4972278	-0.511<br>[-0.742, -0.180]<br>0.0054114	-0.619<br>[-0.772, -0.304]<br>0.00057890	-0.415<br>[-0.703, -0.061]<br>0.0280098	0.426<br>[NA, NA]<br>0.1251937	0.091<br>[-0.392, 0.587]<br>0.711923	-0.067<br>[-0.622, 0.447]<br>0.796253
**TNF-alpha - plasma**	0.131<br>[-0.280, 0.493]<br>0.453390	0.009<br>[-0.385, 0.361]<br>0.9597489	0.007<br>[-0.348, 0.358]<br>0.969407	-0.076<br>[-0.437, 0.286]<br>0.66257103	0.090<br>[-0.294, 0.462]<br>0.6184347	-0.473<br>[-0.718, -0.170]<br>0.0040762	-0.370<br>[-0.671, -0.017]<br>0.03113721	-0.420<br>[-0.660, -0.093]<br>0.0119588	0.114<br>[-0.323, 0.534]<br>0.6390846	0.152<br>[-0.300, 0.562]<br>0.484991	0.084<br>[-0.369, 0.518]<br>0.713570
**NGF- synovial fluid**	-0.052<br>[-0.343, 0.269]<br>0.767597	0.111<br>[-0.235, 0.420]<br>0.5246115	0.047<br>[-0.257, 0.359]<br>0.787501	-0.206<br>[-0.511, 0.108]<br>0.23442602	0.068<br>[-0.243, 0.364]<br>0.7079094	-0.031<br>[-0.303, 0.320]<br>0.8587336	-0.036<br>[-0.394, 0.311]<br>0.83824586	-0.017<br>[-0.342, 0.335]<br>0.9212038	0.119<br>[-0.361, 0.575]<br>0.6213281	0.243<br>[-0.212, 0.619]<br>0.264448	0.274<br>[-0.183, 0.695]<br>0.231269
**NGF - plasma**	-0.012<br>[-0.322, 0.308]<br>0.945470	0.034<br>[-0.294, 0.348]<br>0.8479228	0.109<br>[-0.211, 0.433]<br>0.534738	-0.139<br>[-0.463, 0.203]<br>0.42455463	-0.064<br>[-0.436, 0.310]<br>0.7225189	-0.067<br>[-0.410, 0.253]<br>0.7036402	-0.094<br>[-0.457, 0.254]<br>0.59867690	0.175<br>[-0.167, 0.496]<br>0.3142315	0.341<br>[NA, NA]<br>0.1579582	0.048<br>[-0.394, 0.490]<br>0.826712	0.316<br>[-0.188, 0.756]<br>0.166987
**Calprotectin - synovial fluid**	-0.122<br>[-0.411, 0.203]<br>0.484876	0.158<br>[-0.168, 0.464]<br>0.3660033	0.061<br>[-0.249, 0.354]<br>0.727834	0.195<br>[-0.138, 0.548]<br>0.26129604	0.006<br>[-0.350, 0.366]<br>0.9719816	-0.099<br>[-0.467, 0.220]<br>0.5709765	0.015<br>[-0.381, 0.391]<br>0.93120018	-0.259<br>[-0.567, 0.083]<br>0.1328285	-0.250<br>[-0.655, 0.227]<br>0.3020798	0.529<br>[0.132, 0.856]<br>0.015401	0.000<br>[-0.478, 0.467]<br>1.000000
**Calprotectin - plasma**	-0.141<br>[-0.472, 0.216]<br>0.418902	-0.242<br>[-0.538, 0.094]<br>0.1609044	-0.210<br>[-0.534, 0.118]<br>0.226111	-0.336<br>[-0.551, -0.038]<br>0.04875032	-0.304<br>[-0.620, 0.044]<br>0.0853108	-0.381<br>[-0.681, -0.029]<br>0.0240987	-0.334<br>[-0.619, 0.006]<br>0.05371741	-0.244<br>[-0.570, 0.080]<br>0.1569812	-0.136<br>[-0.627, 0.333]<br>0.5735882	-0.238<br>[-0.615, 0.190]<br>0.275234	-0.211<br>[-0.590, 0.217]<br>0.358795
**Leptin - synovial fluid**	0.209<br>[-0.147, 0.559]<br>0.227269	0.325<br>[-0.004, 0.637]<br>0.0570118	0.295<br>[-0.049, 0.585]<br>0.085203	0.405<br>[0.090, 0.659]<br>0.01568681	0.410<br>[0.059, 0.689]<br>0.0179554	-0.079<br>[-0.403, 0.277]<br>0.6516151	0.063<br>[-0.329, 0.437]<br>0.72252699	-0.055<br>[-0.401, 0.304]<br>0.7541151	0.284<br>[-0.250, 0.769]<br>0.2410174	-0.143<br>[-0.558, 0.276]<br>0.512691	0.295<br>[-0.155, 0.733]<br>0.198887
**Leptin - plasma**	0.008<br>[-0.321, 0.318]<br>0.964180	-0.030<br>[-0.369, 0.320]<br>0.8638458	0.031<br>[-0.260, 0.339]<br>0.860739	0.344<br>[0.013, 0.584]<br>0.04297202	0.192<br>[-0.183, 0.520]<br>0.2853605	0.079<br>[-0.274, 0.430]<br>0.6515922	0.276<br>[-0.065, 0.602]<br>0.11400339	0.116<br>[-0.241, 0.462]<br>0.5068793	0.205<br>[-0.273, 0.660]<br>0.3985263	0.000<br>[-0.442, 0.444]<br>1.000000	-0.005<br>[-0.529, 0.522]<br>0.981695
**Irisin - synovial fluid**	0.048<br>[-0.310, 0.396]<br>0.786235	0.183<br>[-0.177, 0.523]<br>0.2924295	0.170<br>[-0.156, 0.514]<br>0.328270	0.405<br>[0.060, 0.669]<br>0.01589698	0.263<br>[-0.154, 0.616]<br>0.1397877	0.160<br>[-0.204, 0.517]<br>0.3593482	0.547<br>[0.238, 0.783]<br>0.00081581	0.201<br>[-0.113, 0.513]<br>0.2477904	0.767<br>[0.471, 0.977]<br>0.0015457	0.138<br>[-0.318, 0.571]<br>0.526793	0.553<br>[0.083, 0.900]<br>0.015989
**Irisin - plasma**	-0.069<br>[-0.420, 0.280]<br>0.699734	-0.095<br>[-0.427, 0.259]<br>0.5948377	0.022<br>[-0.298, 0.352]<br>0.901609	0.119<br>[-0.242, 0.450]<br>0.50237127	-0.050<br>[-0.452, 0.390]<br>0.7864792	0.190<br>[-0.187, 0.521]<br>0.2826495	0.309<br>[-0.063, 0.615]<br>0.08008344	0.185<br>[-0.146, 0.508]<br>0.2960624	-0.083<br>[-0.584, 0.479]<br>0.7325198	0.087<br>[-0.372, 0.562]<br>0.695928	-0.244<br>[-0.661, 0.273]<br>0.291178
**Adiponectin - synovial fluid**	0.014<br>[-0.399, 0.396]<br>0.938297	0.150<br>[-0.222, 0.508]<br>0.3902796	-0.143<br>[-0.481, 0.241]<br>0.413901	0.252<br>[-0.136, 0.598]<br>0.14473156	0.244<br>[-0.133, 0.569]<br>0.1716438	0.081<br>[-0.262, 0.400]<br>0.6428179	0.056<br>[-0.288, 0.391]<br>0.75393000	-0.057<br>[-0.400, 0.337]<br>0.7428617	0.057<br>[-0.412, 0.510]<br>0.8145036	0.081<br>[-0.351, 0.524]<br>0.710489	0.211<br>[-0.245, 0.623]<br>0.358499
**Adiponectin - plasma**	0.174<br>[-0.217, 0.512]<br>0.318717	0.130<br>[-0.221, 0.495]<br>0.4572707	0.065<br>[-0.285, 0.397]<br>0.712335	0.100<br>[-0.295, 0.451]<br>0.56767561	0.122<br>[-0.266, 0.489]<br>0.4972296	0.329<br>[-0.031, 0.630]<br>0.0536746	0.430<br>[0.103, 0.718]<br>0.01107059	0.420<br>[0.068, 0.703]<br>0.0120269	-0.250<br>[NA, NA]<br>0.3021874	0.000<br>[-0.457, 0.452]<br>1.000000	-0.347<br>[-0.705, 0.066]<br>0.129989
**Omentin - synovial fluid**	0.151<br>[-0.240, 0.529]<br>0.410803	0.351<br>[-0.099, 0.723]<br>0.0486118	0.099<br>[-0.341, 0.494]<br>0.588345	0.363<br>[-0.022, 0.642]<br>0.04116644	0.232<br>[-0.166, 0.550]<br>0.2182067	0.004<br>[-0.324, 0.344]<br>0.9811311	0.203<br>[-0.179, 0.520]<br>0.27392591	-0.124<br>[-0.446, 0.220]<br>0.4989268	0.099<br>[-0.444, 0.630]<br>0.6904043	0.161<br>[-0.321, 0.612]<br>0.487379	0.071<br>[-0.400, 0.565]<br>0.763183
**Omentin - plasma**	0.097<br>[-0.272, 0.473]<br>0.580470	-0.201<br>[-0.560, 0.187]<br>0.2471811	-0.054<br>[-0.370, 0.271]<br>0.759071	-0.150<br>[-0.502, 0.222]<br>0.38859955	0.175<br>[-0.235, 0.555]<br>0.3297701	0.227<br>[-0.165, 0.577]<br>0.1892307	0.293<br>[-0.121, 0.668]<br>0.09249030	0.242<br>[-0.141, 0.599]<br>0.1618564	-0.006<br>[-0.602, 0.540]<br>0.9812901	0.200<br>[-0.260, 0.642]<br>0.359338	-0.068<br>[-0.534, 0.467]<br>0.765492
**Osteopontin - synovial fluid**	0.411<br>[0.108, 0.668]<br>0.014083	0.386<br>[0.042, 0.678]<br>0.0219505	0.210<br>[-0.097, 0.494]<br>0.225970	0.094<br>[-0.263, 0.412<br>0.59196781	-0.118<br>[-0.417, 0.239]<br>0.5127265	-0.107<br>[-0.433, 0.266]<br>0.5423825	-0.077<br>[-0.398, 0.316]<br>0.66543353	-0.181<br>[-0.473, 0.160]<br>0.2971746	-0.244<br>[-0.688, 0.344]<br>0.3130388	0.005<br>[-0.409, 0.441]<br>0.982579	-0.205<br>[-0.654, 0.309]<br>0.370641
**Osteopontin - plasma**	0.020<br>[-0.315, 0.376]<br>0.911244	0.051<br>[-0.264, 0.369]<br>0.7698393	0.134<br>[-0.197, 0.439]<br>0.444344	-0.044<br>[-0.372, 0.319]<br>0.80106804	-0.023<br>[-0.350, 0.306]<br>0.8985330	-0.003<br>[-0.313, 0.343]<br>0.9856129	0.235<br>[-0.070, 0.495]<br>0.18089512	0.052[<br>-0.285, 0.381]<br>0.7653307	0.091<br>[-0.363, 0.534]<br>0.7075242	0.124<br>[-0.305, 0.538]<br>0.570465	0.295<br>[NA, NA]<br>0.198887
**Visfatin - synovial fluid**	0.236<br>[-0.134, 0.553]<br>0.173074	0.260<br>[-0.103, 0.559]<br>0.1321914	0.183<br>[-0.137, 0.481]<br>0.291861	0.013<br>[-0.320, 0.340]<br>0.94263559	0.061<br>[-0.335, 0.432]<br>0.7357113	0.046<br>[-0.288, 0.402]<br>0.7913034	0.034<br>[-0.375, 0.397]<br>0.84999387	-0.059<br>[-0.427, 0.294]<br>0.7377116	0.068<br>[-0.379, 0.524]<br>0.7784133	0.133<br>[-0.294, 0.558]<br>0.541193	0.105<br>[-0.343, 0.522]<br>0.646355
**Visfatin - plasma**	0.267<br>[-0.049, 0.565]<br>0.120680	0.102<br>[-0.222, 0.393]<br>0.5614540	0.266<br>[-0.042, 0.528]<br>0.122476	0.061<br>[-0.318, 0.421]<br>0.72655629	-0.008<br>[-0.396, 0.363]<br>0.9646272	-0.247<br>[-0.525, 0.116]<br>0.1530834	-0.226<br>[-0.543, 0.099]<br>0.19795756	-0.184<br>[-0.466, 0.159]<br>0.2893346	-0.057<br>[-0.534, 0.432]<br>0.8146050	-0.505<br>[-0.857, -0.107]<br>0.020717	-0.147<br>[-0.538, 0.304]<br>0.520637
**Resistin - synovial fluid**	0.195<br>[-0.161, 0.509]<br>0.260592	0.167<br>[-0.177, 0.496]<br>0.3364753	0.247<br>[-0.114, 0.557]<br>0.151854	-0.047<br>[-0.352, 0.283]<br>0.78927343	-0.247<br>[-0.526, 0.093]<br>0.1655403	0.089<br>[-0.262, 0.413]<br>0.6128236	-0.062<br>[-0.388, 0.261]<br>0.72701323	0.078<br>[-0.280, 0.445]<br>0.6555455	-0.364<br>[-0.773, 0.079]<br>0.1332518	0.257<br>[-0.186, 0.644]<br>0.238415	-0.211<br>[-0.643, 0.261]<br>0.358558
**Resistin - plasma**	-0.027<br>[-0.355, 0.328]<br>0.875723	-0.046<br>[-0.394, 0.290]<br>0.7913969	-0.022<br>[-0.411, 0.353]<br>0.900794	-0.169<br>[-0.496, 0.184]<br>0.33144085	-0.066<br>[-0.456, 0.320]<br>0.7155499	-0.242<br>[-0.534, 0.078]<br>0.1604565	-0.519<br>[-0.725, -0.231]<br>0.00165920	-0.230<br>[-0.535, 0.078]<br>0.1839812	-0.420<br>[-0.792, 0.022]<br>0.0827014	-0.248<br>[-0.676, 0.226]<br>0.256487	-0.168<br>[-0.619, 0.317<br>0.462869

Supplementary Table S8. Association of synovial and plasma cytokines with clinical severity in Knee Osteoarthritis Inflammatory Phenotypes (KOIP), adjusted for age, time of disease's evolution and Body Mass Index (BMI). The table presents Spearman correlation coefficients, 95% confidence intervals (95% CI) and p-values, illustrating the association between Knee Osteoarthritis (KOA) severity measures (KOOS-pain, KOOS-functional disability, and ultrasound joint effusion) and selected cytokines analyzed in the study. The analyses are adjusted for age, disease duration, and BMI. Results are presented for the entire patient cohort as well as for individual KOIP groups. **KOA:** Knee Osteoarthritis; **KOIP:** Knee Osteoarthritis Inflammatory Phenotypes (KOIP); **KOOS:** Knee injury and Osteoarthritis Outcome Scores (reversed scores); **95%CI:** 95% confidence interval.

KOA Severity	Cytokine	All		KOIP-1		KOIP-2		KOIP-3		KOIP-4	
		Spearman correlation [95%CI]	P-value	Spearman correlation [95%CI]	P-value	Spearman correlation [95%CI]	P-value	Spearman correlation [95%CI]	P-value	Spearman correlation [95%CI]	P-value
KOOS - Pain (reversed, 0-100)	Omentin - synovial fluid (pg/mL)	0.099 [-0.057, 0.249]	0.21221	-0.241 [-0.487, 0.040]	0.092329	0.300 [-0.025, 0.567]	0.070156	0.146 [-0.209, 0.467]	0.42272	0.231 [-0.199, 0.586]	0.29104
	Osteopontin - synovial fluid (ng/mL)	0.206 [0.055, 0.348]	0.007798	0.118 [-0.176, 0.393]	0.43198	0.114 [-0.207, 0.413]	0.48995	0.098 [-0.349, 0.508]	0.67805	0.461 [0.153, 0.687]	0.0044899
	Interleukin 6 - plasma (pg/mL)	0.007 [-0.147, 0.160]	0.93076	0.047 [-0.240, 0.327]	0.75032	0.027 [-0.261, 0.310]	0.85728	0.130 [-0.366, 0.568]	0.61906	-0.328 [-0.634, 0.067]	0.10145
KOOS - Functional Disability (reversed, 0-100)	Omentin - synovial fluid (pg/mL)	0.060 [-0.095, 0.213]	0.44518	-0.202 [-0.446, 0.069]	0.14207	0.247 [-0.079, 0.524]	0.13584	0.194 [-0.207, 0.539]	0.34369	0.206 [-0.241, 0.581]	0.36769
	Interleukin 6 - plasma (pg/mL)	0.029 [-0.124, 0.182]	0.70824	0.164 [-0.122, 0.425]	0.26152	0.067 [-0.211, 0.335]	0.64094	-0.215 [-0.587, 0.233]	0.34812	-0.299 [-0.565, 0.023]	0.068168
Ultrasound Joint Effusion (mm)	C-reactive protein - synovial fluid (mg/L)	0.007 [-0.146, 0.160]	0.926	0.059 [-0.238, 0.346]	0.70114	0.041 [-0.251, 0.326]	0.78781	0.324 [-0.062, 0.626]	0.097677	-0.570 [-0.763, -0.284]	0.00035628
	C-reactive protein - plasma (mg/L)	-0.029 [-0.181, 0.124]	0.70813	-0.146 [-0.403, 0.133]	0.30445	-0.041 [-0.329, 0.254]	0.78949	0.412 [0.089, 0.657]	0.013912	-0.538 [-0.748, -0.231]	0.0012901
	Leptin - synovial fluid (pg/mL)	0.058 [-0.095, 0.209]	0.45711	-0.123 [-0.410, 0.187]	0.43981	-0.184 [-0.440, 0.099]	0.2007	0.030 [-0.410, 0.458]	0.89965	0.429 [0.091, 0.678]	0.014337
	Leptin - plasma (pg/mL)	-0.062 [-0.213, 0.093]	0.4338	-0.166 [-0.418, 0.110]	0.23865	-0.194 [-0.458, 0.101]	0.19584	-0.034 [-0.420, 0.363]	0.87195	0.253 [-0.099, 0.548]	0.15632
	Irisin - synovial fluid (ng/mL)	0.019 [-0.136, 0.173]	0.81176	-0.183 [-0.434, 0.095]	0.19624	-0.107 [-0.420, 0.228]	0.53419	0.259 [-0.151, 0.593]	0.2124	0.326 [-0.021, 0.603]	0.064766
	Interleukin 6 - synovial fluid (pg/mL)	0.236 [0.086, 0.376]	0.0023444	0.103 [-0.169, 0.361]	0.45915	0.157 [-0.144, 0.430]	0.30618	0.634 [0.390, 0.795]	0.000012813	0.355 [0.035, 0.608]	0.030222
	Interleukin 6 - plasma (pg/mL)	0.055 [-0.099, 0.206]	0.48514	0.118 [-0.169, 0.387]	0.42041	0.142 [-0.149, 0.409]	0.33893	0.051 [-0.351, 0.437]	0.80907	-0.321 [-0.598, 0.025]	0.068546
	Calprotectin - plasma (ng/mL)	-0.003 [-0.156, 0.150]	0.96899	-0.044 [-0.306, 0.224]	0.75206	0.141 [-0.132, 0.394]	0.3115	-0.052 [-0.438, 0.349]	0.8052	-0.308 [-0.554, -0.013]	0.040914
	Omentin - synovial fluid (pg/mL)	0.162 [0.008, 0.308]	0.040004	0.098 [-0.190, 0.370]	0.50697	0.129 [-0.184, 0.418]	0.42145	0.123 [-0.270, 0.480]	0.54479	0.362 [0.021, 0.628]	0.038155

Supplementary Table S9. Association of synovial and plasma cytokines with radiographic progression in Knee Osteoarthritis Inflammatory Phenotypes (KOIP), adjusted for age, time of disease's evolution and Body Mass Index (BMI). The table presents fold-changes, 95% confidence intervals (95% CI) and p-values, illustrating the association between Knee Osteoarthritis (KOA) progression according to different radiographic criteria (Kellgren-Lawrence, Osteophytes and Joint Space Narrowing) and selected cytokines analyzed in the study. Positive fold-changes indicate a higher level of the cytokine in progressors, while negative fold-changes represents a higher level of the cytokine in non-progressors. The analyses are adjusted for age, disease duration, and BMI. Results are presented for the entire patient cohort as well as for individual KOIP groups. **KOA:** Knee Osteoarthritis; **KOIP:** Knee Osteoarthritis Inflammatory Phenotypes (KOIP); **95%CI:** 95% confidence interval.

KOA Radiographic Progression	Cytokines	All		KOIP-1		KOIP-2		KOIP-3		KOIP-4	
		Fold-Change [95%CI]	P-value	Fold-Change [95%CI]	P-value	Fold-Change [95%CI]	P-value	Fold-Change [95%CI]	P-value	Fold-Change [95%CI]	P-value
Kellgren-Lawrence Radiographic Progression	Resistin - synovial fluid (pg/mL)	-1.24 [-1.74, 1.18]	0.24462	-2.08 [-3.65, -1.20]	0.012175	1.51 [-1.41, 2.89]	0.27554	1.31 [-2.27, 3.79]	0.58863	-1.24 [-2.91, 1.88]	0.63494
	C-reactive protein - synovial fluid (mg/L)	1.12 [-1.16, 1.49]	0.39035	1.51 [1.05, 2.19]	0.037626	1.06 [-1.70, 1.81]	0.85564	-1.57 [-2.92, 1.13]	0.11665	1.11 [-1.82, 2.06]	0.75714
	Omentin - plasma (pg/mL)	-1.26 [-1.64, 1.04]	0.076489	-1.40 [-2.20, 1.07]	0.12058	1.06 [-1.45, 1.56]	0.81773	-2.70 [-7.00, -1.31]	0.009627	1.05 [-1.66, 1.70]	0.86185
	Irisin - synovial fluid (ng/mL)	-1.04 [-1.18, 1.10]	0.60394	-1.25 [-1.47, -1.06]	0.008212	-1.07 [-1.49, 1.30]	0.68488	-1.16 [-1.74, 1.31]	0.47166	1.39 [1.14, 1.66]	0.0012974
Osteophytes Radiographic Progression	Osteopontin - synovial fluid (ng/mL)	-1.21 [-1.63, 1.10]	0.19176	1.37 [-1.36, 2.54]	0.33122	-1.45 [-2.30, 1.09]	0.12429	-1.10 [-2.64, 2.15]	0.78894	-1.10 [-1.78, 1.41]	0.64559
	Osteopontin - plasma (ng/mL)	-1.14 [-1.37, 1.04]	0.14962	-1.05 [-1.58, 1.40]	0.78026	-1.42 [-1.98, -1.04]	0.031466	-1.38 [-2.66, 1.42]	0.33039	1.11 [-1.27, 1.60]	0.55478
	Omentin - synovial fluid (pg/mL)	1.02 [-1.32, 1.42]	0.88754	1.85 [1.08, 3.37]	0.028051	-1.72 [-2.92, -1.06]	0.03584	1.05 [-3.20, 3.59]	0.90198	1.41 [-1.36, 2.79]	0.28672
	Omentin - plasma (pg/mL)	1.02 [-1.25, 1.31]	0.86418	1.86 [1.10, 3.64]	0.030952	-1.15 [-1.64, 1.26]	0.46302	-1.76 [-5.02, 1.61]	0.24345	1.36 [-1.17, 2.15]	0.19517
	Leptin - synovial fluid (pg/mL)	1.05 [-1.19, 1.30]	0.68843	1.74 [1.03, 2.94]	0.034066	-1.15 [-1.53, 1.19]	0.38946	-1.08 [-2.10, 1.75]	0.80688	-1.41 [-2.35, 1.16]	0.19797
	Leptin - plasma (pg/mL)	1.05 [-1.14, 1.26]	0.61333	1.43 [1.02, 2.01]	0.044663	-1.05 [-1.50, 1.39]	0.84667	-1.69 [-3.47, 1.37]	0.1892	1.02 [-1.37, 1.43]	0.92611
	Irisin - synovial fluid (ng/mL)	-1.08 [-1.22, 1.05]	0.27223	1.09 [-1.13, 1.34]	0.40431	-1.19 [-1.61, 1.12]	0.23591	-1.68 [-2.64, -1.09]	0.031111	1.19 [-1.00, 1.43]	0.066103
	Irisin - plasma (ng/mL)	-1.03 [-1.17, 1.11]	0.66774	1.17 [-1.02, 1.41]	0.087542	-1.15 [-1.59, 1.25]	0.41349	-1.34 [-2.34, 1.24]	0.22328	1.02 [-1.23, 1.28]	0.84938
	Interleukin 8 - plasma (pg/mL)	1.21 [-1.09, 1.55]	0.1495	1.25 [-1.40, 2.05]	0.35809	1.53 [1.06, 2.23]	0.026868	1.49 [-2.98, 6.09]	0.57305	-1.55 [-2.44, 1.01]	0.045448
	Calprotectin - synovial fluid (ng/mL)	1.33 [-1.06, 1.91]	0.12387	-1.30 [-2.31, 1.46]	0.41521	1.45 [-1.50, 3.34]	0.3442	2.30 [-1.03, 5.71]	0.066658	2.09 [1.05, 4.29]	0.041223
	Adiponectin - synovial fluid (ng/mL)	1.14 [-1.16, 1.48]	0.36748	1.66 [1.07, 2.51]	0.032595	-1.47 [-2.35, 1.06]	0.099879	-1.05 [-2.86, 2.52]	0.90321	1.16 [-1.47, 2.06]	0.54318
Joint Space Narrowing Radiographic Progression	Osteopontin - synovial fluid (ng/mL)	1.26 [-1.04, 1.68]	0.108	1.31 [-1.28, 2.14]	0.25957	1.02 [-1.62, 1.79]	0.87745	2.59 [1.68, 3.99]	0.00034539	-1.32 [-2.04, 1.17]	0.20825
	JoOmentin - synovial fluid (pg/mL)	1.24 [-1.08, 1.65]	0.16296	1.67 [1.12, 2.50]	0.010921	1.22 [-1.57, 2.07]	0.50537	-1.04 [-2.67, 2.33]	0.94272	1.05 [-1.90, 1.98]	0.89549
	Leptin - synovial fluid (pg/mL)	-1.04 [-1.30, 1.22]	0.74315	-1.06 [-1.56, 1.34]	0.73046	-1.67 [-2.30, -1.20]	0.0013274	1.15 [-1.43, 1.84]	0.59876	1.16 [-1.59, 1.97]	0.54063
	Leptin - plasma (pg/mL)	-1.14 [-1.42, 1.05]	0.15722	-1.07 [-1.44, 1.22]	0.55993	-1.60 [-2.32, -1.09]	0.016944	-1.26 [-2.31, 1.41]	0.46167	-1.07 [-1.54, 1.32]	0.66752
	Irisin - synovial fluid (ng/mL)	1.01 [-1.11, 1.14]	0.84859	-1.12 [-1.30, 1.06]	0.15696	-1.14 [-1.55, 1.21]	0.40734	1.20 [-1.23, 1.76]	0.34398	1.26 [1.05, 1.49]	0.012932
	Irisin - plasma (ng/mL)	-1.16 [-1.33, -1.02]	0.021346	-1.10 [-1.24, 1.03]	0.14487	-1.54 [-2.67, -1.01]	0.045653	-1.22 [-1.92, 1.20]	0.28199	-1.15 [-1.51, 1.11]	0.26456
